# Solubility and Selectivity Effects of the Anion on the Adsorption of Different Heavy Metal Ions onto Chitosan

**DOI:** 10.3390/molecules25112482

**Published:** 2020-05-27

**Authors:** Janek Weißpflog, Alexander Gündel, David Vehlow, Christine Steinbach, Martin Müller, Regine Boldt, Simona Schwarz, Dana Schwarz

**Affiliations:** Leibniz-Institut für Polymerforschung Dresden e.V., Hohe Straße 6, 01069 Dresden, Germany; weisspflog@ipfdd.de (J.W.); guendel@ipfdd.de (A.G.); vehlow@ipfdd.de (D.V.); steinbach@ipfdd.de (C.S.); mamuller@ipfdd.de (M.M.); boldt@ipfdd.de (R.B.); simsch@ipfdd.de (S.S.)

**Keywords:** chitosan, heavy metal ions, adsorption, water treatment, anions

## Abstract

The biopolymer chitosan is a very efficient adsorber material for the removal of heavy metal ions from aqueous solutions. Due to the solubility properties of chitosan it can be used as both a liquid adsorber and a solid flocculant for water treatment reaching outstanding adsorption capacities for a number of heavy metal ions. However, the type of anion corresponding to the investigated heavy metal ions has a strong influence on the adsorption capacity and sorption mechanism on chitosan. In this work, the adsorption capacity of the heavy metal ions manganese, iron, cobalt, nickel, copper, and zinc were investigated in dependence on their corresponding anions sulfate, chloride, and nitrate by batch experiments. The selectivity of the different heavy metal ions was analyzed by column experiments.

## 1. Introduction

Pollution caused by humankind is becoming an ever-increasing problem on a daily basis [1,2,3]. Much of the pollution occurs at surface waters like rivers, lakes, and, of course, in oceans [4,5]. Particularly difficult is the separation of toxic substances, which have an essential impact on the environment even at low concentrations [6]. Such substances can be heavy metal cations in surface waters due to a lack of safety precautions or accidents from industrial waste [7]. Therefore, it is important to remove these substances from surface waters, and especially from drinking water. In recent years, intensive research has been conducted on how to remove these inorganic ions from aqueous solutions [8,9,10]. Known and applied methods include precipitation, ion exchange, and various membrane processes [11,12,13]. As a result to the increased demand of economic processes to remove metal ions from industrial wastewater, biomaterials, in particular chitosan, have been intensively studied in recent years [14,15,16,17]. Thus, chitosan is very suitable for the adsorption of heavy metal cations mainly due to its primary amino groups. Chitosan is a biodegradable polysaccharide derived from chitin, which is found in the shells of crustaceans and thus a waste product from food industry. Chitin is the most abundant biopolymer after cellulose and mainly obtained from crab and shrimp tanks [18,19,20]. Chitin consists of linear chains of *N*-acetyl-d-glucosamine. Chitosan is produced by treating chitin with sodium hydroxide or enzymes (deacetylase). In recent years, the use of chitosan as an adsorbent has gained increasing importance. The adsorption capacity depends on several factors such as degree of deacetylation (DD), particle size and crystallinity [21,22,23,24]. The molar mass plays a subordinate role for the adsorption capacity. The DD indicates the ratio of free amino groups present for interaction with the metal ions. In addition, amino groups have a higher reactivity than acetamide groups [25,26,27,28,29]. Hence, it can be concluded that a higher DD has a positive effect on the adsorption capacity. Since the associated higher costs in the production are problematic, chitosan with a DD of <95% is very rarely used in industry. The particle size depends on the application and can be easily modified. Of course, nanoparticles offer themselves for adsorption processes because they have the well-known large surface/volume ratio offering high surface areas for respective adsorbates. However, these can add small holes in column tests and, thus, clog the apparatus. Other forms are sponges, flakes, fibers, or hollow fibers. In addition, chitosan can also be chemically modified by crosslinking. Bi- and tri-functional chemicals such as glutaraldehyde or tripolyphosphate are commonly used to create a three-dimensional network to increase the acid resistance of chitosan and to improve the adsorption performance, for example of alkali metal ions [30].

In this study, we investigated the adsorption behavior of chitosan with a DD of 85% for the heavy metal ions manganese, iron, cobalt, nickel, copper, and zinc ions in dependence on the corresponding anion sulfate, chloride, and nitrate. Previously, we studied the adsorption of CuSO_4_, FeSO_4_, and NiSO_4_ on chitosan and could show that besides the known adsorption of the heavy metal ion, the sulfate ions adsorb as well [31]. Subsequently, we used NiSO_4_ and Ni(NO_3_)_2_ to understand the influence of the oxyanion for the adsorption of heavy metal ions on chitosan on the example of nickel salts [32]. The adsorption isotherms showed huge differences in the adsorption capacities for nickel in dependence of the corresponding anion. Here, we extended the number of examined heavy metal ions and anions to comprehend the overall adsorption mechanism and the influence of the anion. Besides the oxyanions sulfate and nitrate, we also analyzed chloride salts motivated by the fact, that chitosan dissolves well in acids such as HCl and acetic acid but not in sulfuric acid [33]. Hence, the solubility of chitosan in the tested metal salt solutions was considered as well.

## 2. Results and Discussions

### 2.1. Batch Experiments

**Figure 1:**
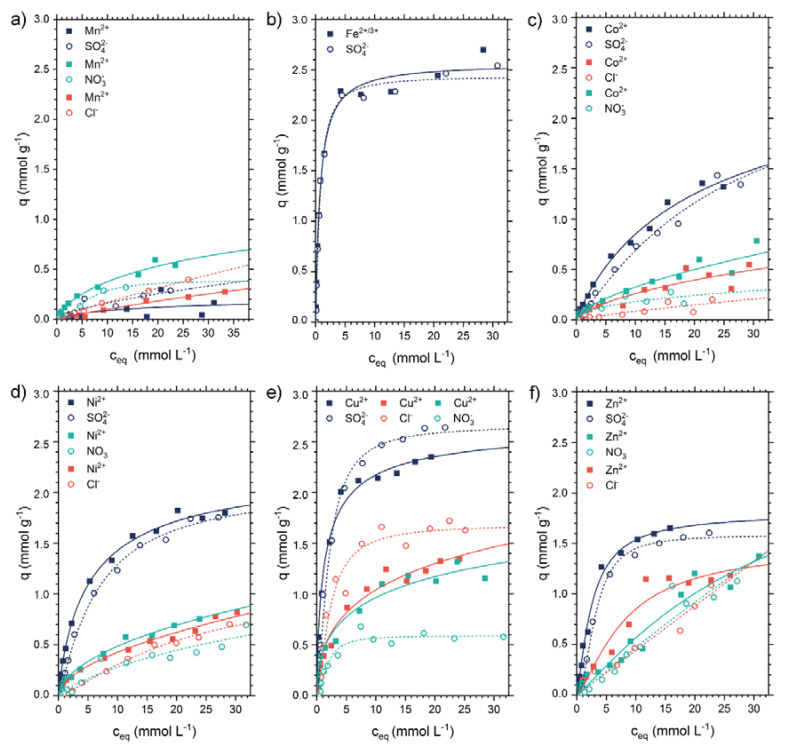
Adsorption isotherms of heavy metal ions (full symbols) and the corresponding anions (open symbols). Batch adsorption experiments with the heavy metal salts: (**a**) MnSO_4_ (blue), MnCl_2_ (red), and Mn(NO_3_)_2_ (green), (**b**) FeSO_4_ (blue), (**c**) CoSO_4_ (blue), CoCl_2_ (red), and Co(NO_3_)_2_ (green), (**d**) NiSO_4_ (blue), NiCl_2_ (red), and Ni(NO_3_)_2_ (green), (**e**) CuSO_4_ (blue), CuCl_2_ (red), and Cu(NO_3_)_2_ (green), and (**f**) ZnSO_4_ (blue), ZnCl_2_ (red), and Zn(NO_3_)_2_ (green) onto chitosan at 298 K dependent on the equilibrium concentration after adsorption process. Langmuir isotherms were fitted for metal ions (solid lines) and Langmuir isotherms for anions (dashed lines). The pH of the solutions was not adjusted (see Figure S1).

The adsorption properties of the six heavy metal ions manganese, iron, cobalt, nickel, copper and zinc were investigated in dependence on their corresponding anion by batch experiments. The adsorption isotherms of all six heavy metal ions and three different anions supported by the Langmuir fits are shown in Figure 1. Thus, for every investigated heavy metal ion the adsorption capacity is highly dependent on the corresponding anion of the metal salt. As previously described, chitosan features the ability to adsorb simultaneously heavy metal ions and oxyanion like sulfate [31,32]. This observation is valid for most heavy metal salts. Except for manganese salts, all heavy metal ions with sulfate exhibit the highest adsorption capacity in comparison to the nitrate and chloride salts. This might be due to the different valence of sulfate (SO_4_^2−^) in comparison to chloride (Cl^−^) and nitrate (NO_3_^−^). Nickel salts are a perfect example for an adsorption of nickel ions and anions with a ratio of 1:1 independent on the type of anion (see Figure 1d). The adsorption capacities obtained for the cobalt salts are slightly lower in comparison to nickel, but show a similar trend. NiSO_4_, ZnSO_4_, and CoSO_4_ featured an adsorption ratio of metal ions to sulfate ions of 1:1 and adsorption capacities between 1.5 mmol·g^−1^ and 2.0 mmol·g^−1^. NiSO_4_ and ZnSO_4_ exhibited higher adsorption values at low concentrations in comparison to CoSO_4_. The adsorption isotherms of the NiSO_4_ and NiCl_2_ exhibited similar adsorption rates for the nickel ions. Similar trends can be observed for the heavy metal ions cobalt, nickel, copper, and zinc as nitrate and chloride salts. Within the series of heavy metal sulfates, iron and copper featured the highest adsorption capacities with around 2.3 mmol·g^−1^ for both, heavy metal ions and around 2.6 mmol·g^−1^ for sulfate ions. However, the curves for the nitrate and chloride ions do not feature a 1:1 adsorption ratio with the copper ions. For copper, the adsorption rate of chloride ions is higher and the adsorption rate of nitrate ions is a lot lower in comparison to copper ions. Although, it should be noted that the adsorption of Cu^2+^ at low concentrations hardly depends on the anion with an adsorption efficiency of 94% for CuCl_2_, 96% for Cu(NO_3_)_2_, and 97% for CuSO_4_. As the concentration increases, the adsorption of the ions CuCl_2_ and Cu(NO_3_)_2_ decreases almost equally and more rapidly than for CuSO_4_.

The adsorption of iron salts on chitosan was unique in comparison to the other heavy metal salts, since chitosan dissolved in FeCl_2(aq)_ and Fe(NO_3_)_3(aq)_. Thus, the turbidity of the solutions increased by the addition of FeCl_2(aq)_ for concentrations above c_0_ = 500 mg L^−1^. However, at a similar concentration range, chitosan dissolved in Fe(NO_3_)_3(aq)_ without visible turbidity leading to the assumption that chitosan is better soluble in Fe(NO_3_)_3(aq)_. Thus, the adsorption of FeCl_2_ and Fe(NO_3_)_3_ was not analyzable. Furthermore, iron has a strong tendency to oxidize from Fe^2+^ and Fe^3+^ being present side by side in solution. The chitosan flakes showed a reddish to reddish-brown color during the adsorption process. The adsorption of Fe^2+/3+^ was up to 95% at low c_0_.

Manganese salts show in general a relatively low adsorption capacity in comparison to the other heavy metal salts. Within the investigated series of divalent cationic heavy metal ions, manganese has the lowest complex stability constant (see Irving-Williams Series). The complex stability constant can only be compared for the same type of anion. Within the Irving-Williams Series copper has the highest complex stability constant and thus the highest adsorption capacities. The maximum adsorption efficiency of Mn^2+^ was 20%, which is considerably lower than to the other metal salts. Especially the very low MnSO_4_ isotherm in comparison to the MnCl_2_ and Mn(NO_3_)_2_ isotherms is contrary to the trend of the other metal salts. The pH values for the heavy metal salt solutions were not adjusted and are shown in Figure S1. The obtained adsorption capacities were compared with other materials in the Supporting Information.

**Figure 2:**
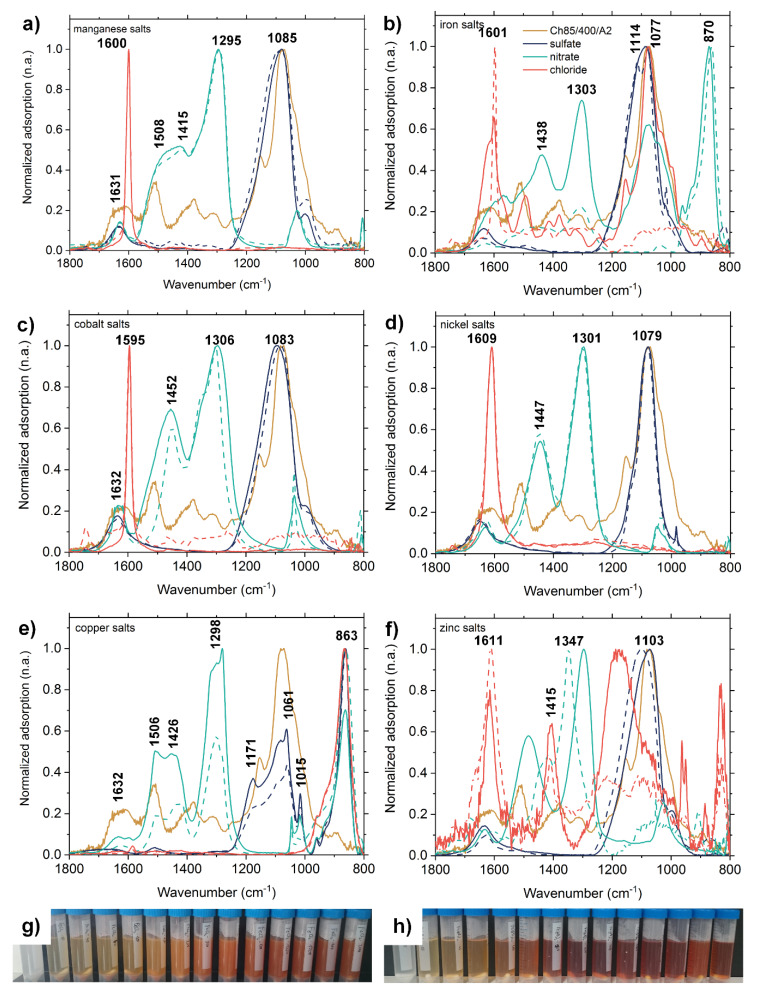
(**a**)–(**f**) show the FTIR spectra of the metal salt solutions before (dashed line) and from the supernatant after (full line) the adsorption. For comparison, the yellow spectrum shows the pure chitosan. (**a**) MnSO_4_ (blue), Mn(NO_3_)_2_ (red), and MnCl_2_ (green); (**b**) FeSO_4_ (blue), Fe(NO_3_)_3_ (red), and FeCl_2_ (green); (**c**) CoSO_4_ (blue), Co(NO_3_)_2_ (red), and CoCl_2_ (green); (**d**) NiSO_4_ (blue), Ni(NO_3_)_2_ (red), and NiCl_2_ (green); (**e**) CuSO_4_ (blue), Cu(NO_3_)_2_ (red), and CuCl_2_ (green); (**f**) ZnSO_4_ (blue), Zn(NO_3_)_2_ (red), and ZnCl_2_ (green), (**g**) adsorption experiments chitosan with FeCl_2_, and (**h**) adsorption experiments of chitosan with Fe(NO_3_)_3_. The concentration of salt solutions was 2 g·L^−1^ in relation to the metal ion.

In order to gain a better understanding of the dissolution and adsorption mechanisms with chitosan FTIR spectroscopy was applied. Figure 2 represents the FTIR spectra of the salt solutions casted onto an internal reflection element and dried (see experimental section in Supporting Information). The FTIR spectra are related to the pure salt solutions before and the supernatants after the adsorption onto Ch85/400/A2.

First of all, the dissolution tendency of the chitosan flakes can be analyzed from these spectra. The chitosan spectrum (Figure 2a–f, in yellow) has some prominent IR peaks at around 1630 cm^−1^ and 1510 cm^−1^ assigned to the Amide I and Amide II band and at around 1050 cm^−1^ due to the ν(C–O) stretching vibration of ether and hydroxyl linkages of polysaccharides. The absence of these bands, most significant for ν(C–O), clearly indicates that the chitosan flakes preserved upon the adsorption process, while presence indicates part dissolution. Applying this criterion to all supernatant spectra in Figure 2, it can be identified that the iron salts FeCl_2(aq)_ and FeNO_3(aq)_ dissolved the chitosan flakes, except for FeSO_4_. Arguments for dissolution only in FeCl_2(aq)_ and FeNO_3(aq)_ could be, that the pH value was significantly lower than for the other ones (see Supporting Information). The exception for FeSO_4_ might be explained by a bridging scenario of chitosan/SO_4_^2−^ stabilizing the sample. It is well known that chitosan is not soluble in sulfuric acid, which is in accordance with this observation. Interestingly, copper salts featured also values pH < 7, but did not dissolve chitosan. Presumably, also copper salts were able to bridge and thus stabilize chitosan against dissolution.

Secondly, in the FTIR spectra of the dried supernatant samples diagnostic bands were observed at around 1300 cm^−1^ assigned to ν(NO_2_) vibration due to nitrate ions and at around 1080 cm^−1^ from the ν(SO_2_) vibration due to sulfate ions, while chloride ions per se cause no IR bands. However, the peaks at around 1600 cm^−1^ for chloride salts are due to the δ(H_2_O) band of bound water in these salts and hence are indirectly diagnostic for chloride salts. From the presence of these salts in the supernatant spectra it can be qualitatively concluded, that the metal salts were not completely bound to chitosan flakes, but quantitative determination of the unbound fraction is challenging. Although shifts of both ν(NO_2_) and ν(SO_2_) were observed for different metal salts, the interpretation is challenging for several reasons. At first, diagnostic shifts of these bands should be only expected, if there was an interaction to chitosan (e.g., electrostatically via the ammonium group, or via the chelation of the metal cation with chitosan). Such an interaction to chitosan could be only observed, if there was dissolution of chitosan, onto which readily metal salts were bound. However, dissolution was only observed for iron salts, but just these salts did not show band shifts. Secondly, positions of ν(SO_2_) and ν(NO_2_) bands of respective sulfate and nitrate ions are crucially dependent on the amount of bound water still present in dry metal salt samples. Commonly, there is a shift to lower wavenumbers of these bands for higher amounts and a shift to higher wavenumbers for lower amounts of bound water. However, this amount is dependent on the drying process and on the hygroscopy of the metal salts. Hence, conclusively the observed shifts should not be related to any binding scenario of metal salts at chitosan at the moment. This issue will be further addressed on chitosan (pellet not supernatant) samples, onto which metal salts were bound.

**Figure 3:**
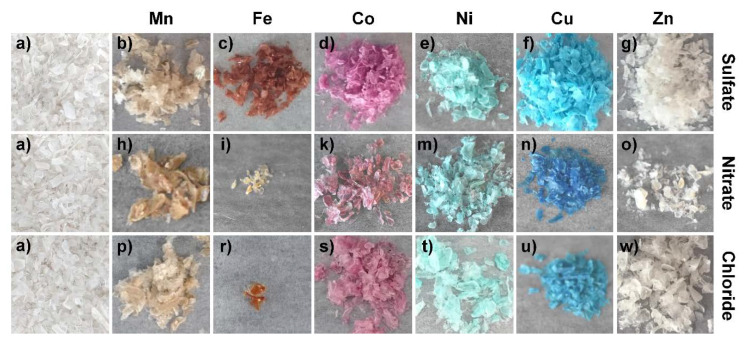
Images of: (**a**) pure chitosan flakes, (**b**) c_0_(MnSO_4_) = 5900 mg·L^−1^, (**h**) c_0_(Mn(NO_3_)_2_) = 5800 mg·L^−1^ and (**p**) c_0_(MnCl_2_) = 4100 mg·L^−1^, (**c**) c_0_(FeSO_4_) = 5900 mg·L^−1^, (**i**) c_0_(Fe(NO_3_)_3_) = 7900 mg·L^−1^, (**r**) c_0_(FeCl_2_) = 4600 mg·L^−1^, (**d**) c_0_(CoSO_4_) = 4800 mg·L^−1^, (**k**) c_0_(Co(NO_3_)_2_) = 6200 mg·L^−1^, and (**s**) c_0_(CoCl_2_) = 4000 mg·L^−1^, (**e**) c_0_(NiSO_4_) = 5600 mg·L^−1^, (**n**) c_0_(Ni(NO_3_)_2_) = 6200 mg·L^−1^, and (**t**) c_0_(NiCl_2_) = 4200 mg·L^−1^, (**f**) c_0_(CuSO_4_) = 4700 mg·L^−1^, (**m**) c_0_(Cu(NO_3_)_2_) = 5800 mg·L^−1^, and (**u**) c_0_(CuCl_2_) = 4000 mg·L^−1^, (**g**) c_0_(ZnSO_4_) = 5200 mg·L^−1^, (**o**) c_0_(Zn(NO_3_)_2_) = 3800 mg·L^−1^, and (**w**) c_0_(ZnCl_2_) = 4100 mg·L^−1^ after the adsorption process. All flakes were rinsed with water before drying after the adsorption process.

With increasing concentration, the adsorption of some heavy metal salts on the chitosan flakes was also visible by naked eye due to the color change of the flakes (see Figure 3). Although Mn(II) salts feature a pink color, which is characteristic for transition metal complexes with high spin d^5^ configurations, the chitosan flakes treated with manganese salts resulted in a brownish color, which might be due to the oxidation of Mn^2+^ to MnO_2_. A similar oxidation procedure can be noticed for the iron salts. After the adsorption of the iron salts, all chitosan flakes featured a brownish orange color, although the pure iron sulfate, nitrate, and chloride are colored pale blue, white grey, and blue green, respectively. The brown orange color might be caused by the formation of iron hydroxide. The amount of flakes treated with iron chloride and iron nitrate is very low as most of the flakes dissolved in the salt solution (see Figure 3i,r). For comparison, the chitosan flakes treated with cobalt, nickel, copper, and zinc salts featured the color, which is similar to the color of the pure salts. In general, the color intensity of the flakes increased for all samples with the concentration of metal salt solutions (see Figure S2).

In order to understand the adsorption mechanism and color changes on the flakes, especially for the manganese and iron salts, the flakes were analyzed by XRD measurements (see Supporting Information, Figures S10–S15). All chitosan samples treated with manganese salts featured a similar XRD pattern, which is mainly the pattern of pure chitosan. There are two small reflections at 31° and 51° 2θ, which could be assigned to the (104) and (116) reflections of MnCO_3_ [34]. However, since the reflections of MnCO_3_ show only low intensity due to the low amount of manganese salts on the chitosan flakes this is just one potential cause. The mineral MnCO_3_ exhibits a pink color, similar to all other Mn(II) species. However, partially oxidized MnCO_3_ features a light brown color, which might also explain the color change of the flakes from white to light brown after the adsorption process (see Figure 3). For the iron-chitosan compounds, compounds with lower salt concentrations were investigated, since the chitosan was dissolved at higher salt concentrations of FeCl_2_ and Fe(NO_3_)_3_. For iron nitrate–chitosan, the concentration may have been too low, so that no salt was detectable. In the case of iron chloride–chitosan and iron sulfate-chitosan, FeO(OH) could be detected. Iron (III) oxide-hydroxide is known for its orange color and used as Pigment Yellow 42. Thus, this explains also the observed color change on the chitosan flakes. The XRD pattern for nickel, cobalt, and zinc salts show mainly the reflections of chitosan due to the low intensities of the salt crystals on the chitosan surface. In comparison, CuCl_2_ and CuSO_4_ salts featured relatively high intensities of the adsorbed salts on the surface. This is in good agreement with high adsorption capacities obtained for copper salts. According to the XRD spectra, all three copper salts crystallized on the chitosan surface with the corresponding anion.

**Figure 4:**
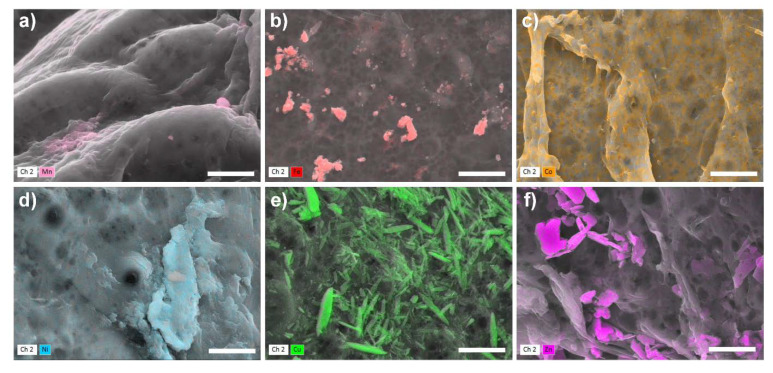
SEM-EDX images of: (**a**) Ch-MnSO_4_ c_0_ = 5800 mg·L^−1^, (**b**) Ch-FeSO_4_ c_0_ = 5900 mg·L^−1^ (**c**) Ch-CoSO_4_ c_0_ = 4800 mg·L^−1^, (**d**) Ch-NiSO_4_ c_0_ = 5600 mg·L^−1^, (**e**) Ch-CuSO_4_ c_0_ = 4700 mg·L^−1^, and (**f**) Ch-ZnSO_4_ c_0_ = 5200 mg·L^−1^ after the adsorption process, scale bar: 3 µm. The SEM-EDX images from all heavy metal salts with the three different anions can be found in the Supporting Information.

The formation of the salts could also be observed by SEM-EDX analysis. Figure 4 shows the surfaces of the chitosan flakes after the adsorption of the six heavy metal sulfate salts. The SEM-EDX images of the chloride and nitrate salts show a similar behavior as the sulfate salts (see Supporting Information). In general, cobalt and nickel salts always feature a comprehensive distribution of the heavy metal ions with the corresponding anions on the surface. The distribution of the nitrate anions cannot be detected by SEM-EDX as pure chitosan features amino groups. Manganese, copper, iron, and zinc are only partially detected as crystals on the surface. However, for all six heavy metal salts the heavy metal ions as well as the corresponding anions sulfate and chloride could be detected by SEM-EDX. For the manganese and copper salts, the anions sulfate and chloride showed a comprehensive distribution over the chitosan flakes. Hence, the adsorption process of the anion is independent on the adsorption process of the heavy metal ion. This result is in accordance with the adsorption isotherms in Figure 1 and the XRD patterns in the Supporting Information.

In general, chitosan features high adsorption capacities for the heavy metal salts of iron, cobalt, nickel, copper, and zinc when using sulfate as corresponding anion. The adsorption processes of heavy metal ions and anions occurs simultaneously. Anions can be adsorbed to chitosan by hydrogen bonding and/or electrostatic forces and/or by the adsorbed heavy metal ions as crystallization process. The adsorption of heavy metal ions onto chitosan is more likely to occur by chelation with the primary amino group (–NH_2_) and the hydroxyl group. Thus, a low pH at which chitosan is partially or fully positively charged is disadvantageous for the adsorption of heavy metal ions. The zeta-potential of chitosan is shown in the Supporting Information, Figure S43. Furthermore, chitosan has the tendency to dissolve at low pH values. The complex formation is based on the principle of Pearson’s “hard soft acid base” (HSAB) [35]. The focus is on the polarizability of Lewis acids and Lewis bases, “soft” acids and bases are easier polarized than “hard” ones. The combination of hard acids and hard bases or soft acids and soft bases results in comparatively more stable complexes than in a combination of hard and soft. The six transition metal ions are intermediate Lewis acids and the chitosan groups RNH_2_ and ROH represent weak ligand bases. Hence, Fe^3+^ and Co^3+^ are weak Lewis acids and should therefore form more stable complexes with chitosan than Fe^2+^ and Co^2+^. Within the series of the six investigated heavy metal ions, copper should feature the highest adsorption capacities as it forms the most stable complexes due to its small metal ion radius, and the larger binding energy and crystal field stabilization energy (CFSE) (Irving-Williams Series). However, as iron oxidizes from Fe^2+^ to Fe^3+^, Fe^3+^ shows a similar high adsorption capacity as copper (II) sulfate. Most studies on the adsorption mechanism of heavy metal ions at chitosan have been carried out with copper ions, due to the high adsorption capacity [24,36,37,38,39]. Two different coordination models, (a) the “bridge model” and (b) the “pendant model” may be applied [31]. On one hand, the bridge model it is assumed that copper ions have the coordination number 4 and are bound by several amino groups by means of intermolecular or intramolecular complex formation [40]. The “pendant model”, on the other hand, describes the binding to an amino group to which the metal ion is attached. The investigations can be performed via X-ray studies of chitosan complexes with different metal cations [41,42,43,44]. In literature, this thesis could be confirmed and it was found that chitosan forms a unique complex at a pH of less than 6.1 with the structure [CuNH_2_(OH)_2_] [43]. Considering the coordination of copper complexes, the fourth side can be occupied by a water molecule or the OH group in the C-3 position [45]. This hypothesis was confirmed using calorimetric measurements. Thus, the pH and the initial concentration have a great influence on the adsorption mechanism and the adsorption capacity. For example, the coordination number changes from 1 at pH 5.3 and to 2 at pH 5.8 [40]. In addition, it has been found that individual monomers, i.e., glucosamine units, are significantly more inefficient in complex formation with copper ions compared to oligomers and polymers. Most likely, the adjacent hydroxyl groups play an important role. It can be assumed that both models of complex formation are possible and it depends on the pH and initial concentration which model is preferred. The same applies to the mechanism of adsorption, which is based on electrostatic forces and complex formation. Hence, the obtained results showed that chitosan metal complexes produced from sulfate salts could achieve the highest adsorption efficiency. The presence of sulfate ions in the solution can therefore facilitate the chelation of the metal ions with the chitosan polymer. Nevertheless, most metal ions are affected by hydroxide precipitation, so that a deposition onto the adsorber as oxides or hydroxides could be possible.

### 2.2. Column Experiments

**Figure 5:**
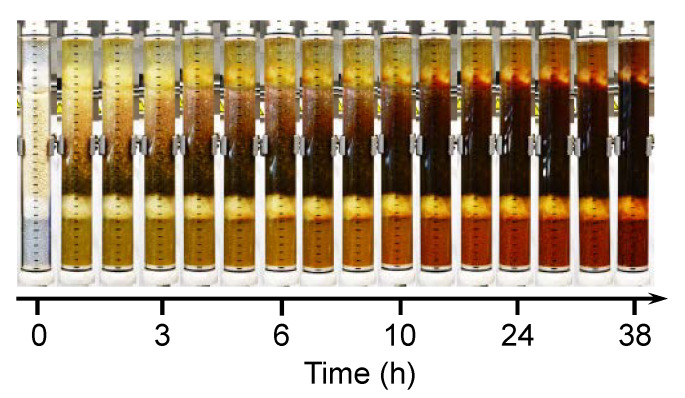
Images of the increasing color change and intensity of the packed bed column as a function of time from left to right; column was filled with chitosan and fixed with glass balls and glass wool at the ends of the column.

In addition to the batch studies, a column experiment was carried out, which should represent the simultaneous separation of the metal ions and sulfate ions used as a function of time. Chitosan flakes were packed in a chromatography column and fixed with glass wool and glass balls. The essential difference to the batch tests is not only the simultaneous dynamic analysis, but also that the separation takes place essentially in the absence of air. The metal salt solution was pumped from below into the column to ensure uniform adsorption. Similar to the batch experiments, an increasing color is recognizable, here as a function of time (see Figure 5).

**Figure 6:**
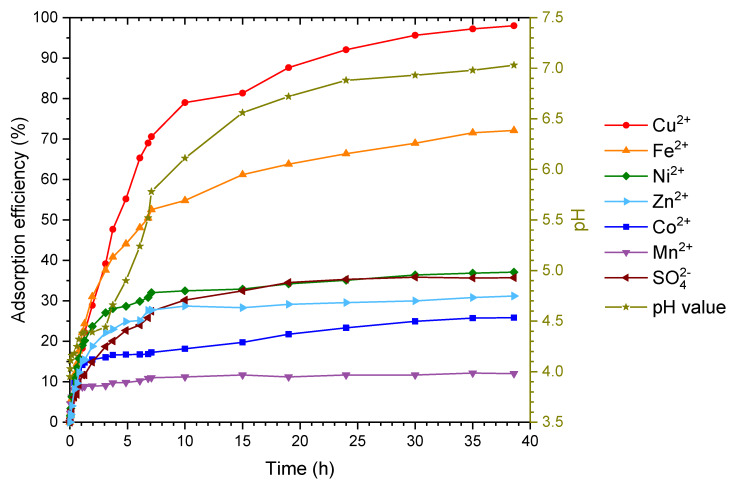
Column experiment with all six heavy metal sulfate salts. The column was filled with the chitosan flakes and the concentration of the heavy metal ions and sulfate was measured as a function of time, including the change in pH. The initial concentrations of each heavy metal ion in the solution was c_0_(Mn^2+^) = 4.06 mmol·L^−1^; c_0_(Fe^2+^) = 4.05 mmol·L^−1^; c_0_(Co^2+^) = 4.08 mmol·L^−1^; c_0_(Ni^2+^) = 3.91 mmol·L^−1^; c_0_(Cu^2+^) = 3.97 mmol·L^−1^; c_0_(Zn^2+^) = 3.93 mmol·L^−1^; and c_0_(SO_4_^2−^) = 28.64 mmol·L^−1^. Graph was also plotted as c(t)/c(0) vs. time (see Figure **S38**) and q(t) vs. time (see Figure **S39**).

In Figure 6, the adsorption of all six heavy metal sulfate salts on chitosan in a column is shown. The separation efficiency is very dependent on the speed of the pump. With a relatively slow flow rate of 0.29 mL·s^−1^ in continuous operation, a good separation of the individual ions over time could be observed. After 38.5 h, a separation efficiency of the metal ions Cu^2+^ > Fe^2+^ > Ni^2+^ = Zn^2+^ > Co^2+^ > Mn^2+^ was detected. As the experiment was performed under anaerobic conditions, it can be assumed that all ions are bivalent. This fact is different to the batch experiments and yields in slightly different results. The equilibrium for manganese ions reached relatively fast after a short time of 1 h with an adsorption rate of 13% (q_max_ = 16%). The separation of cobalt ions with q_max_ = 23% is slightly better under the given conditions. It seems that these depend more strongly on the adsorption of the other metal ions, since separation increases continuously, albeit slowly. The separation of the nickel ions (q_max_ = 37%) and zinc ions (q_max_ = 37%) is similar, whereby the maximum separation rate seems to be reached almost after 7 h. After 38.5 h, iron ions were separated to q_max_ = 89%. Copper ions reached the highest adsorption efficiency with q_max_ = 98%. Due to the considerable amount of adsorber used, the pH also increased during the course of the experiment from pH_0_ = 3.95 to pH_max_ = 7.03.

**Figure 7:**
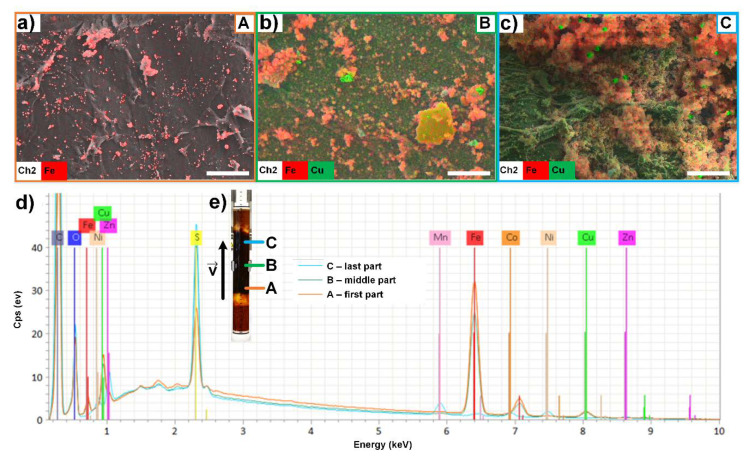
SEM-EDX results of selected samples from the column after the adsorption process. SEM-EDX images of (**a**) first part (**A**) of the column, (**b**) the middle part (**B**) of the column, and (**c**) last part (**C**) of the column (beginning, middle and end of the column in the direction of flow). Scale bar shows 20 µm. (**d**) EDX spectra of the SEM-EDX images in (**a**–**c,e**) Image of the column divided into the first part **A**, the middle part **B**, and the last part **C**.

After the adsorption process, the chitosan flakes were dried and analyzed by SEM-EDX. As an example, chitosan flakes were analyzed from the first part (A) of the column, from the middle as well as from the last part (C) (Figure 7). It can be stated by the SEM images that the composition of the samples differ (see Figure 7a–c). The surface of the chitosan flakes from the last part of the column features a reduced roughness in comparison to the chitosan flakes from the first part of the column. It can be concluded that the metal salt deposition increases from the first part to the last part of the column in direction of flow of the metal salt solution. The composition of the metal ions also differs due to this gradient. While in the first part of the column primarily iron ions and copper ions were adsorbed, manganese ions or zinc ions were mainly detected in the last part of the column (see Figure 7d,e).

## 3. Materials and Methods

### 3.1. Materials

#### 3.1.1. Chitosan

Chitosan in form of flakes with a deacetylation degree of 85% (product name Ch85/400/A2) was purchased from the company BioLog Heppe^®^ GmbH, Landsberg, Germany, and used as received.

#### 3.1.2. Heavy Metal Salts

Iron (II) sulfate heptahydrate (FeSO_4_·7H_2_O or FeSO_4_ (aq)) and iron (III) nitrate nonahydrate (Fe(NO_3_)_3_·9H_2_O) were purchased from Carl Roth GmbH + Co. KG, Karlsruhe, Germany. Nickel(II) sulfate hexahydrate (NiSO_4_·6H_2_O), copper (II) sulfate (CuSO_4_), cobalt (II) sulfate heptahydrate (CoSO_4_·7H_2_O), iron (II)-chloride (FeCl_2_), copper (II) chloride dihydrate (CuCl_2_·2H_2_O), copper (II) nitrate hemi(pentahydrate) (Cu(NO_3_)_2_·2.5H_2_O), zinc (II) nitrate hydrate (Zn(NO_3_)_2_·H_2_O), nickel (II) chloride hexahydrate (NiCl_2_·6H_2_O), nickel (II) nitrate hexahydrate (Ni(NO_3_)_2_·6H_2_O), cobalt (II) chloride hexahydrate (CoCl_2_·6H_2_O), cobalt (II) nitrate hexahydrate (Co(NO_3_)_2_·6H_2_O), manganese (II) chloride (MnCl_2_), manganese (II) nitrate tetrahydrate (Mn(NO_3_)_2_·4H_2_O), and zinc(II) sulfate monohydrate (ZnSO_4_·H_2_O) were purchased from Sigma-Aldrich Chemie GmbH, Steinheim, Germany. Manganese (II) sulfate monohydrate (MnSO_4_·H_2_O) and zinc (II) chloride (ZnCl_2_) were purchased from Merck KGaA, Darmstadt, Germany. By default, all solutions and any flushing processes were made with deionized water from a Milli-Q-Advantage A10 from Merck Millipore (Darmstadt, Germany). The produced deionized water is ultrapure water from purity degree of type 1, which was added with pretreated water. It is characterized by a constant conductivity of 0.055 µS·cm^−1^, respectively 18.2 MΩ·cm, at a temperature of 298 K.

### 3.2. Adsorption Experiments

#### 3.2.1. Batch

As we have shown previously, it is known that the adsorption equilibrium is reached after 24 h [31]. Adsorption experiments were performed in 50 mL centrifuge tubes at room temperature and 24 h stirring with a magnetic stirrer at 600 U·min^−1^. The heavy metal ion solutions were prepared from a stock solution with a concentration of 2 g·L^−1^. 30 mL of the heavy metal ion solutions were added to 0.1 g chitosan flakes. The heavy metal ion solutions were prepared with Milli-Q water without any additional adjustment of the pH. The pH value was measured of every initial solution (pH_0_) and after the adsorption process (pH_eq_). After 24 h, 20 mL of the supernatant were removed. Afterwards, the chitosan flakes were rinsed with distilled water three times to make sure that they are not surrounded by heavy metal ion solution anymore. The flakes were dried for further investigations. The concentration of all heavy metal ions and the amount of sulfate ions of the initial solutions as well as after the adsorption process were investigated by ICP-OES measurements. The concentrations of the anions chloride and nitrate were determined by UV/Vis measurements.

#### 3.2.2. Packed Bed Column Reactors (PBCR)

From a storage vessel, a certain volume with a selected concentration was pumped through a packed bed column filled with chitosan flakes. The chitosan flakes were filled loosely, distributed without pressure in the column. The chitosan flakes were fixed in the column with glass wool and glass beads (at the entrance and exit of the column). Before adsorption process, the column was rinsed with deionized water for 1 h at a flow velocity of 291 μL·s^−1^. Due to the fact that the chitosan flakes in the column will swell by contact with water, an anaerobic process is ensured. The used column had the following dimensions: total height_column_ = 38 cm; filled height_package_ = 13.5 cm; internal diameter = 3.6 cm; mass_Chitosan_ = 26.8 g. Then, 5.5 L of the heavy metal salt solution was pumped through the column. The concentration of each metal ion was approx. 4 mmol·L^−1^. The sulfate ion concentration c_0_ = 28.64 mmol·L^−1^ results from the mix of salts with a pH value of 4. The flow velocity of the solution during adsorption process was set at 291 μL·s^−1^ resulting in a column dead time of about 15:30 min.

## 4. Conclusions

In this study, the adsorption behavior of heavy metal ions, depending on the type of anion, onto chitosan was thoroughly investigated by batch and column experiments. In summary, the heavy metal cations of the sulfate salts and the sulfate ions themselves adsorb to a significantly higher extent compared to the analogous chloride and nitrate salts, respectively. For sulfate salts, the following order of adsorption capacities could be obtained: Cu^2+^ (2.35 mmol·g^−1^) > Fe^2+/3+^ (2.31 mmol·g^−1^) > Ni^2+^ (1.82 mmol·g^−1^) > Zn^2+^ (1.65 mmol·g^−1^) > Co^2+^ (1.41 mmol·g^−1^) > Mn^2+^. The adsorption capacities of the heavy metal ions in the presence of chloride or nitrate ions were the same except for manganese and iron. The coexistence of protonated and non-protonated amino groups makes it possible to bind cations via complex formation and anions by electrostatic forces, as proven by ICP-OES and SEM-EDX investigations. As a result, chitosan with a DD of 85% is well suited as adsorbent for heavy metal ions. The elemental distribution on the surface of chitosan was analyzed by EDX studies. The observed crystals on the chitosan surface could be correlated to the heavy metal ion but only in some cases to the anions. Sulfate or chloride ions were evenly distributed over the entire surface except for Ch-ZnCl_2_ and Ch-NiSO_4_. These two samples showed that nickel and sulfate or zinc and chloride ions are adsorbed separately. Thus, the adsorption of heavy metal ions is strongly dependent on the corresponding anion and both ions, cation and anion, are simultaneously adsorbed by chitosan.

## Supplementary Materials

The following are available online, Table S1: Instrument and method parameters for ICP-OES 7400. Figure S1: pH changes before (pH_0_) and after (pH_eq_) the adsorption process for (a) manganese salts, (b) iron salts, (c) cobalt salts, (d) nickel salts, (e) copper salts, (f) zinc salts in dependence of the anions (sulfate—blue, chloride—red, and nitrate—green) and concentration at room temperature. Figure S2: Images of (a) MnSO_4_, (b) Mn(NO_3_)_2_ and (c) MnCl_2_, (d) FeSO_4_, (e) Fe(NO_3_)_3_, (f) FeCl_2_, (g) CoSO_4_, (h) Co(NO_3_)_2_, and (i) CoCl_2_, (k) NiSO_4_, (m) Ni(NO_3_)_2_, and (n) NiCl_2_, (o) CuSO_4_, (p) Cu(NO_3_)_2_, and (r) CuCl_2_, (s) ZnSO_4_, (t) Zn(NO_3_)_2_, and (u) ZnCl_2_ after the adsorption process. Concentrations used from left-hand-side to right-hand-side: 0 mg·L^−1^, 20 mg·L^−1^, 40 mg·L^−1^, 60 mg·L^−1^, 100 mg·L^−1^, 150 mg·L^−1^, 250 mg·L^−1^, 500 mg·L^−1^, 750 mg·L^−1^, 1000 mg·L^−1^, 1250 mg·L^−1^, 1500 mg·L^−1^, 1750 mg·L^−1^, and 2000 mg·L^−1^ (initial metal ion concentration) and rinsed with water before drying after the adsorption process. Figure S3: X-ray diffractograms of Ch85/400/A2 used as received (green line) and after rinsing the flakes with deionized water (red line). Figure S4: X-ray diffraction patterns of the chitosan flakes after the adsorption process with manganese nitrate c_0_ = 5800 mg·L^−1^ (green), manganese chloride c_0_ = 4100 mg·L^−1^ (red), and manganese sulfate c_0_ = 5900 mg·L^−1^ (blue). The pattern in yellow show the pure MnCO_3_ for comparison. The samples were measured on a silicon wafer, which shows a reflection at 2θ 56.5° (labeled with a black star). Figure S5: X-ray diffraction patterns of the chitosan flakes after the adsorption process with iron nitrate c_0_ = 410 mg·L^−1^ (green), iron chloride c_0_ = 650 mg·L^−1^ (red), and iron sulfate c_0_ = 5900 mg·L^−1^ (blue). The pattern in yellow show the pure FeO(OH) for comparison. The samples were measured on a silicon wafer, which shows a reflection at 2θ 56.5° (labeled with a black star). Figure S6: X-ray diffraction patterns of the chitosan flakes after the adsorption process with cobalt nitrate c_0_ = 6200 mg·L^−1^ (green), cobalt chloride c_0_ = 4000 mg·L^−1^ (red), and cobalt sulfate c_0_ = 4800 mg·L^−1^ (blue). The pattern in yellow shows the pure CoCl_2_ for comparison. The samples were measured on a silicon wafer, which shows a reflection at 2θ 56.5° (labeled with a black star). Figure S7: X-ray diffraction patterns of the chitosan flakes after the adsorption process with nickel nitrate c_0_ = 6200 mg·L^−1^ (green), nickel chloride c_0_ = 4200 mg·L^−1^ (red), and nickel sulfate c_0_ = 5600 mg·L^−1^ (blue). The samples were measured on a silicon wafer, which shows a reflection at 2θ 56.5° (labeled with a black star). Figure S8: X-ray diffraction patterns of the chitosan flakes after the adsorption process with copper nitrate c_0_ = 5800 mg·L^−1^ (green), copper chloride c_0_ = 4000 mg·L^−1^ (red), and copper sulfate c_0_ = 4700 mg·L^−1^ (blue). The pattern in yellow shows the pure copper salts, which show a good match with the measured pattern. The samples were measured on a silicon wafer, which shows a reflection at 2θ 56.5° (labeled with a black star). Figure S9: X-ray diffraction patterns of the chitosan flakes after the adsorption process with zinc nitrate c_0_ = 3800 mg·L^−1^ (green), zinc chloride c_0_ = 4100 mg·L^−1^ (red), and zinc sulfate c_0_ = 5200 mg·L^−1^ (blue). The pattern in yellow shows the pure zinc salt Zn_4_SO_4_(OH)_6_·5H_2_O, which show a good match with the measured pattern. The samples were measured on a silicon wafer, which shows a reflection at 2θ 56.5° (labeled with a black star). Figure S10: SEM images of manganese chloride (a) c_0_ = 50 mg·L^−1^ and (b) c_0_ = 4000 mg·L^−1^; manganese nitrate (c) c_0_ = 60 mg·L^−1^ and (d) c_0_ = 440 mg·L^−1^; manganese sulfate (e) c_0_ = 70 mg·L^−1^ and (f) c_0_ = 6000 mg·L^−1^. Figure S11: SEM images of iron chloride Iron chloride (a) c_0_ = 50 mg·L^−1^ and (b) c_0_ = 4600 mg·L^−1^; iron nitrate (c) c_0_ = 90 mg·L^−1^ and (d) c_0_ = 600 mg·L^−1^; iron sulfate (e) c_0_ = 80 mg·L^−1^, and (f) c_0_ = 5900 mg·L^−1^. Figure S12: SEM images of cobalt chloride (a) c_0_ = 50 mg·L^−1^ and (b) c_0_ = 4000 mg·L^−1^; cobalt nitrate (c) c_0_ = 60 mg·L^−1^ and (d) c_0_ = 480 mg·L^−1^; cobalt sulfate (e) c_0_ = 50 mg·L^−1^ and (f) c_0_ = 4800 mg·L^−1^. Figure S13: SEM images of nickel chloride (a) c_0_ = 50 mg·L^−1^ and (b) c_0_ = 4200 mg·L^−1^; nickel nitrate (c) c_0_ = 60 mg·L^−1^ and (d) c_0_ = 470 mg·L^−1^; nickel sulfate (e) c_0_ = 60 mg·L^−1^ and (f) c_0_ = 5600 mg·L^−1^. Figure S14: SEM images of copper chloride (a) c_0_ = 40 mg·L^−1^ and (b) c_0_ = 4000 mg·L^−1^; copper nitrate (c) c_0_ = 50 mg·L^−1^ and (d) c_0_ = 440 mg·L^−1^; copper sulfate (e) c_0_ = 50 mg·L^−1^ and (f) c_0_ = 4700 mg·L^−1^. Figure S15: SEM images of zinc chloride (a) c_0_ = 40 mg·L^−1^ and (b) c_0_ = 4100 mg·L^−1^; zinc nitrate (c) c_0_ = 30 mg·L^−1^ and (d) c_0_ = 260 mg·L^−1^; zinc sulfate (e) c_0_ = 60 mg·L^−1^ and (f) c_0_ = 5200 mg·L^−1^. Figure S16: EDX pattern of Ch-Mn(NO_3_)_2_ (c_0_ = 5800 mg·L^−1^) after the adsorption process. Figure S17: EDX pattern of Ch-Fe(NO_3_)_2_ (c_0_ = 410 mg·L^−1^) after the adsorption process. Figure S18: EDX pattern of Ch-Co(NO_3_)_2_ (c_0_ = 6200 mg·L^−1^) after the adsorption process. Figure S19: EDX pattern of Ch-Ni(NO_3_)_2_ (c_0_ = 6200 mg·L^−1^) after the adsorption process. Figure S20: EDX pattern of Ch-Cu(NO_3_)_2_ (c_0_ = 5800 mg·L^−1^) after the adsorption process. Figure S21: EDX pattern of Ch-Zn(NO_3_)_2_ (c_0_ = 3800 mg·L^−1^) after the adsorption process. Figure S22: EDX pattern of Ch-MnCl_2_ (c_0_ = 4100 mg·L^−1^) after the adsorption process. Figure S23: EDX pattern of Ch-FeCl_2_ (c_0_ = 650 mg·L^−1^) after the adsorption process. Figure S24: EDX pattern of Ch-CoCl_2_ (c_0_ = 4000 mg·L^−1^) after the adsorption process. Figure S25: EDX pattern of Ch-NiCl_2_ (c_0_ = 4200 mg·L^−1^) after the adsorption process. Figure S26: EDX pattern of Ch-CuCl_2_ (c_0_ = 4000 mg·L^−1^) after the adsorption process. Figure S27: EDX pattern of Ch-ZnCl_2_ (c_0_ = 4100 mg·L^−1^) after the adsorption process. Figure S28: EDX pattern of Ch-MnSO_4_ (c_0_ = 5900 mg·L^−1^) after the adsorption process. Figure S29: EDX pattern of Ch-FeSO_4_ (c_0_ = 5900 mg·L^−1^) after the adsorption process. Figure S30: EDX pattern of Ch-CoSO_4_ (c_0_ = 4800 mg·L^−1^) after the adsorption process. Figure S31: EDX pattern of Ch-NiSO_4_ (c_0_ = 5600 mg·L^−1^) after the adsorption process. Figure S32: EDX pattern of Ch-CuSO_4_ (c_0_ = 4700 mg·L^−1^) after the adsorption process. Figure S33: EDX pattern of Ch-ZnSO_4_ (c_0_ = 5200 mg·L^−1^) after the adsorption process. Figure S34 SEM–EDX images of (a) Ch-MnCl_2_ c_0_ = 4100 mg·L^−1^ (distribution of Cl, top corner right-hand-side), (b) Ch-CoCl_2_ c_0_ = 4000 mg·L^−1^ (distribution of Cl, top corner right-hand-side), (c) Ch-Mn(NO_3_)_2_ c_0_ = 5800 mg·L^−1^, (d) Ch-Co(NO_3_)_2_ c_0_ = 6200 mg·L^−1^, (e) Ch-MnSO_4_ c_0_ = 5900 mg·L^−1^ (distribution of S, top corner right-hand-side), and (f) Ch-CoSO_4_ c_0_ = 4800 mg·L^−1^ (distribution of S, top corner right-hand-side) after the adsorption process, scale bar: 3 µm. Figure S35: SEM–EDX images of (a) Ch-NiCl_2_ c_0_ = 4200 mg·L^−1^ (distribution of Cl, top corner right-hand-side), (b) Ch-CuCl_2_ c_0_ = 4000 mg·L^−1^ (distribution of Cl, top corner right-hand-side), (c) Ch-Ni(NO_3_)_2_ c_0_ = 6200 mg·L^−1^, (d) Ch-Cu(NO_3_)_2_ c_0_ = 5800 mg·L^−1^, (e) Ch-NiSO_4_ c_0_ = 5600 mg·L^−1^ (distribution of S, top corner right-hand-side), and (f) Ch-CuSO_4_ c_0_ = 4700 mg·L^−1^ (distribution of S, top corner right-hand-side) after the adsorption process, scale bar: 3 µm. Figure S36: SEM–EDX images of (a) Ch-ZnCl_2_ c_0_ = 4100 mg·L^−1^ (distribution of Cl, top corner right-hand-side), (b) Ch-FeCl_2_ c_0_ = 650 mg·L^−1^, (c) Ch-Zn(NO_3_)_2_ c_0_ = 3800 mg·L^−1^, (d) Ch-Fe(NO_3_)_2_ c_0_ = 410 mg·L^−1^, (e) Ch-ZnSO_4_ c_0_ = 5200 mg·L^−1^ (distribution of S, top corner right-hand-side), and (f) Ch-FeSO_4_ c_0_ = 5900 mg·L^−1^ (distribution of S, top corner right-hand-side) after the adsorption process, scale bar: 3 µm. Figure S37: Zetapotential measurement as a function of pH for Ch85/400/A2. Figure S38 Column experiment as c**_t_**/c**_0_** vs. time plot with all six heavy metal sulfate salts. The column was filled with the chitosan flakes and the concentration of the heavy metal ions and sulfate was measured as a function of time, including the change in pH. The initial concentrations of each heavy metal ion in the solution was c_0_(Mn^2+^) = 4.06 mmol·L^−1^; c_0_(Fe^2+^) = 4.05 mmol·L^−1^; c_0_(Co^2+^) = 4.08 mmol·L^−1^; c_0_(Ni^2+^) = 3.91 mmol·L^−1^; c_0_(Cu^2+^) = 3.97 mmol·L^−1^; c_0_(Zn^2+^) = 3.93 mmol·L^−1^; and c_0_(SO_4_^2−^) = 28.64 mmol·L^−1^. Figure S39: Column experiment as q(t) vs. time plot with all six heavy metal sulfate salts. The column was filled with the chitosan flakes and the concentration of the heavy metal ions and sulfate was measured as a function of time, including the change in pH. The initial concentrations of each heavy metal ion in the solution was c_0_(Mn^2+^) = 4.06 mmol·L^−1^; c_0_(Fe^2+^) = 4.05 mmol·L^−1^; c_0_(Co^2+^) = 4.08 mmol·L^−1^; c_0_(Ni^2+^) = 3.91 mmol·L^−1^; c_0_(Cu^2+^) = 3.97 mmol·L^−1^; c_0_(Zn^2+^) = 3.93 mmol·L^−1^; and c_0_(SO_4_^2-^) = 28.64 mmol·L^−1^. Table S2: Adsorption capacities for removal of manganese compounds from aqueous solutions with different adsorber materials. Table S3: Adsorption capacities for removal of iron compounds from aqueous solutions with different adsorber materials. Table S4: Adsorption capacities for removal of cobalt compounds from aqueous solutions with different adsorber materials. Table S5: Adsorption capacities for removal of nickel compounds from aqueous solutions with different adsorber materials. Table S6: Adsorption capacities for removal of copper compounds from aqueous solutions with different adsorber materials. Table S7: Adsorption capacities for removal of zinc compounds from aqueous solutions with different adsorber materials.
