# Supplementary material for: Solubility and Selectivity Effects of the Anion on the Adsorption of Different Heavy Metal Ions onto Chitosan
Source: Molecules. 2020 May 27;25(11):2482. doi: 10.3390/molecules25112482 (PMC7321104; doi:10.3390/molecules25112482)
Supplement: Supplementary file 1 [file molecules-25-02482-s001.pdf]

ELECTRONIC SUPPLEMENTARY INFORMATION

for the manuscript

Solubility and Selectivity Effects of the Anion on the Adsorption of Different Heavy Metal Ions onto Chitosan

Janek Weißpflog [1], Alexander Gündel [1], David Vehlow [1], Christine Steinbach [1], Martin Müller [1], Regine Boldt [1], Simona Schwarz [1], Dana Schwarz [1,*]

[1] Affiliation 1; Leibniz-Institut für Polymerforschung Dresden e.V., Hohe Straße 6, 01069 Dresden, Germany; weisspflog@ipfdd.de (J.W.); guendel@ipfdd.de (A.G.); vehlow@ipfdd.de (D.V.); steinbach@ipfdd.de (C.S.); boldt@ipfdd.de (R.B.); mamuller@ipfdd.de (M.M.); simsch@ipfdd.de (S.S.)

* Correspondence: schwarz-dana@ipfdd.de; Tel.: +49-351-4658-542

Received: date; Accepted: date; Published: date

Abstract: The biopolymer chitosan is a very efficient adsorber material for the removal of heavy metal ions from aqueous solutions. Due to the solubility properties of chitosan it can be used as both liquid adsorber and solid flocculant for water treatment reaching outstanding adsorption capacities for a number of heavy metal ions. However, the type of anion corresponding to the investigated heavy metal ions has a strong influence on the adsorption capacity and sorption mechanism on chitosan. In this work, the adsorption capacity of the heavy metal ions manganese, iron, cobalt, nickel, copper, zinc were investigated in dependence on their corresponding anions sulfate, chloride, nitrate by batch experiments. The selectivity of the different heavy metal ions was analyzed by column experiments.

Table of Content

Table of Content 2
1 Methods 3
1.1 Analytical methods 3
1.1.1 ICP-OES 3
1.1.2 UV-Vis analysis 3
1.1.3 SEM-EDX analysis 4
1.1.4 SEM 4
1.1.5 IR-Spectroscopy 4
1.1.6 XRD-Analysis 4
1.1.7 Zetapotential measurement of Chitosan 4
1.2 Theoretical models 5
2 Results 6
2.1 PH values before and after the adsorption processes 6
2.2 XRD Analysis 9
2.3 SEM Images 16
2.4 EDX-Analysis 22
2.5 SEM-EDX 31
2.6 Zetapotential pH Measurement 34
2.7 Column Experiment 35
3 Classification of the obtained adsorption capacities with other materials 37
4 References 40

1 Methods

1.1 Analytical methods

1.1.1 ICP-OES

Before and after the adsorption processes the concentration of the relevant elements in aqueous solution was determined using an inductively coupled plasma optical emission spectrometer (ICP-OES) from Thermo Scientific™ iCAP 7400 instrument. The software was Qtegra™ Intelligent Scientific Data Solution™. Each investigated sample was measured three times. Fresh standards were prepared to construct the calibration curve. The standard curve was constructed using six calibration points: 0, 62.5 mg L^{-1}, 125 mg L^{-1}, 250 mg L^{-1}, 500 mg L^{-1}, and 1000 mg L^{-1} of each element. The calibration standard solutions were purchased from CPI international (Amsterdam/Netherlands). Many wavelengths were chosen for each element to determine the best one. The selection of the wavelength was based on the calibration curve and the calibration check readings with a low standard deviation and no interferences. From the values obtained, the best wavelengths were selected (see Table S1).

Table S1: Instrument and method parameters for ICP-OES 7400.

Parameter	Value
Nebulizer	Mira Mist
Spray Chamber	Glass cyclonic
Torch	Glass
Injector tube (mm)	2
Autosampler	ASX-280
Nebulizer gas flow (L min^{-1})	0.5
RF power (W)	1150
Flush time (s)	20
Pump speed (rpm)	50
Exposure time UV (low, 166-230 nm) (s)	15
Exposure time Vis (high, 230-847 nm) (s)	5
View mode UV (low, 166-230 nm)	Axial
View mode Vis (high, 230-847 nm)	Radial
Mn (nm)	257.610 {131}
Fe (nm)	261.187 {129}
Ni (nm)	352.454 {96}
Co (nm)	238.892 {141}
Zn (nm)	213.856 {457}
Cu (nm)	224.700 {450}
S (nm)	182.624 {484}

1.1.2 UV-Vis analysis

In addition to the possible detection of the elements by ICP-OES, UV-Vis analysis was used to detect nitrate ions and chloride ions. For this a DR6000 spectrophotometer from the company HACH Lange® GmbH (Duesseldorf, Germany) was used. By default, commercially available cuvette test systems were used for this device. These are based on a color reaction or precipitation for each characteristic ion. The nitrate ion detection was carried out with the a LCK 340 cuvette test (HACH Lange® GmbH) and the chloride ion detection with the a LCK 311 cuvette test (HACH Lange® GmbH).

1.1.3 SEM-EDX analysis

After the adsorption process, chitosan flakes were analyzed using a combination of scanning electron microscope (SEM) and energy-dispersive X-ray spectroscopy (EDX). First, the samples were fixed with double-sided adhesive carbon tape on an aluminum pin sample tray and steamed with C before the investigation starts. For this purpose, the sputter coater BalTec SCD 500 (Leica Microsystems GmbH, Wetzlar, Germany) was used. The measurements were carried out with a SEM UltraPlus (Carl Zeiss Microscopy GmbH, Jena, Germany). This is equipped with an EDX detector XFlash Quad 5060F (Bruker Nano GmbH, Germany) and was analyzed with an excitation voltage at 12 keV with different magnifications.

1.1.4 SEM

SEM investigations were performed with an Ultra plus (Carl Zeiss NTS). The excitation voltage was 3 kV with a SE2 detector. The samples were sputtered with a film of 3 nm Pt (Sputter Coater SCD 050 from Bel Tec).

1.1.5 IR-Spectroscopy

Fourier transform infrared spectroscopy (FTIR) was performed on a Vertex 70 spectrometer from Bruker Optics GmbH (Ettlingen, Germany) in attenuated total reflection (ATR) mode using a 4-mirror-ATR attachment from Perkin Elmer GmbH (Überlingen, Germany). 100 µL of each sample was solution casted at a germanium internal reflection element (IRE). Intensity spectra of the uncoated Ge IRE (I_R) and the sample coated one (I_S) were measured based on 100 scans in the infrared range from 4000 cm^{-1} to 400 cm^{-1} adopting a spectral resolution of 2 cm^{-1}. Absorbance spectra were calculated according to $A = -\log (I_S / I_R)$. Further, we used the atmospheric water vapor compensation tool of the OPUS software from Bruker Optics GmbH (Ettlingen, Germany) to obtain FTIR spectra without disturbing narrow signals from rotational transitions of ν(OH) and δ(OH) vibrations of water vapor. The FTIR spectra were baseline corrected and plotted in the range of 1800 cm^{-1} to 800 cm^{-1}. Generally, spectra of dried pure salt solutions (2 g L^{-1} in relation to the metal ion) were compared with the spectra of mixed chitosan/salt solutions aiming to detect a specific chitosan absorption band. Approximately equal pH values of the solutions are necessary for comparison and a proper spectral analysis. Occasionally, pH values of solutions had to be readjusted to be able to compare them.

1.1.6 XRD-Analysis

To characterize the adsorption efficiency onto the chitosan flakes, they were rinsed with Milli-Q water after the adsorption process, dried at room temperature and investigated by XRD analysis. The XRD profiles were recorded using X-ray 2-cycle diffractometer XRD 3003 T/T (GE Sensing & Inspection Technologies GmbH, Ahrensburg/Germany; former: Seifert GmbH) with multilayer monochromator. Asymmetric reflection measurements have been made with a small angle of incidence. The grazing incidence X-ray diffraction (GIXRD) measurement geometry combined with a long secondary Soller collimator (angular divergence 0.7°) for 2θ was used due to the heterogenic nature of the samples, and for a better intensity. For this purpose, the samples were densely packed on a Si wafer. A 2θ measurement was carried out from 3° to 65° with a constant $\omega = \alpha_i = 3°$. The measurements were conducted at 40 kV and 30 mA, Cu-K_α radiation (monochromatized with Goebel mirror, $\lambda = 1.5418$ Å), measuring time per measuring point: 5 s; increment: 0.05°, at room temperature. To identify the salts on the substrates, the most significant reflections were selected and compared from the ICDD PDF Library Database with salts saved therein.

1.1.7 Zetapotential measurement of Chitosan

Chitosan was characterized by pH titration from a pH range from 3 to 10 with the particle charge detector (MÜTEK, PCD-04) with respect to the state of charge. For this purpose, a 1% (w/v) solution of Ch85/400/A2 was prepared by dissolving 1 g chitosan in 100 mL of 1% (v/v) acetic acid. The solution

was stirred for 24 h at 296 K (rt) before analysis. The streaming potential vs. pH curve is shown in Figure S37.

1.2 Theoretical models

In order to investigate the relationship between the amount of heavy metal ions and sulfate ions adsorbed by chitosan the equilibrium conditions in aqueous solution as well as Langmuir isotherm model was used in this study.

The adsorption efficiency (percentage removal) of the ions was calculated according to the following equation:

$$\text{adsorption efficiency in \%} = \frac{(c_0 - c_e)}{c_0} \cdot 100 \qquad \textbf{(1-1)}$$

with c_0 as the initial concentration and c_{eq} the equilibrium concentration after the adsorption process (mg L^{-1}).

The adsorption capacity at equilibrium state was calculated by mass balance in a batch reactor:

$$q_e = \frac{(c_0 - c_e) \cdot V_L}{m_A} \qquad \textbf{(1-2)}$$

with q_e (mg g^{-1}) the adsorption capacity, m_A the mass (g) of chitosan flakes and V_L (L) the current solution volume loaded in the batch reactor.

The Langmuir isotherm model has been often used to correlate equilibrium adsorption data. The surface is composed of specific energetically equivalent and homogenous sites, which can bind only one molecule, i.e. the adsorption is localized and the adsorbed molecules are not mobile on the surface. Furthermore, there is no interaction between the adsorbed molecules; the heat of adsorption is constant and independent of the coverage. When the surface is saturated by a monomolecular layer of adsorbate the maximum adsorption is reached. The Langmuir isotherm model can be presented as:

$$q_e = \frac{q_{m,L}\ K_L \cdot c_e}{(1 + K_L \cdot c_e)} \qquad \textbf{(1-3)}$$

with q_e and $q_{m,L}$ (mmol g^{-1}), respectively, representing the equilibrium and maximum adsorption capacity and K_L is the Langmuir adsorption constant related to the affinity of the binding sites of the adsorber.

2 Results

2.1 PH values before and after the adsorption processes

The changes in the pH of the starting solution (c_0) and after adsorption (c_{eq}) are shown in Figure S1. The pH of the starting solution (c_0) was measured directly after the preparation of the solution. Figure S1 shows that the adsorption onto Ch85/400/A2 generally causes an increase in the pH of the supernatant. The pH_{eq} is between 8.3 and 8.7 for c_0 = 0 mg L^{-1}. The increase in pH is due to the slightly basic character of chitosan, which was treated with NaOH during the deacetylation process. The higher the concentration of the respective salt, the lower the pH_{eq} within a concentration series (usually pH_{max} < 6). This applies to all salts except for $NiCl_2$ (pH_{max} = 6.71), $ZnCl_2$ (pH_{max} = 6.34) and to a smaller extent $Ni(NO_3)_2$ (pH_{max} = 6.14). After adsorption, the salt solutions with manganese, cobalt and nickel ions indicate pH increases in the range of 7 - 8.

The pH_0 of **manganese** sulfate is lower than that of manganese nitrate and manganese chloride, both have very similar pH_0. After adsorption, pH_{eq} of the three salts are at a level of about 7.5. The pH_0 for **cobalt** salt solutions drops as already known from other salts with increasing salt concentration. The lowest pH_0 showed cobalt sulfate with 4.6 followed by cobalt chloride with 4.9 and cobalt nitrate with 5.2. The pH does not differ as much as the adsorption onto chitosan of the different cobalt salts. After adsorption, pH_{eq} of cobalt nitrate and cobalt chloride were both raised to 7.2, cobalt sulfate even to pH 7.5. For **nickel** chloride there is an exception, by increasing c_0 also the pH_0 increases. Nickel sulfate has the lowest pH_0 of 5.4, followed by nickel nitrate with 6.1 and nickel chloride with 6.71. After adsorption, the pH_{eq} of nickel sulfate increased to 7.6, the other two salts being between 7.1 and 7.2. The pH values differ more strongly among one another than with the iron or copper salt solutions. The pH_0 decreases slightly with increasing c_0 and is about 4.6 for the three **copper** salt solutions. For copper sulfate and copper chloride, at the highest c_0 $pH_0 = pH_{eq}$. For copper nitrate, the pH_{eq} is about 0.6 above pH_0. Only for the copper salt solutions the difference of before and after adsorption is lower. Even after adsorption, the pH_{eq} of the solutions is in the range of 5 - 5.5. The pH_0 values differ similarly to the **zinc** salt solutions. For the zinc salt solutions, pH_{eq} is slightly below of 7, but with a similar trend. The pH_0 curve decreases with increasing concentration, also for zinc sulfate and zinc nitrate with 5.1 and 4.7, respectively, at c_0 = 2 g L^{-1}. For zinc chloride, however, the pH_0 increases with increasing concentration and ends up at 6.3. The pH_{eq} decreases with increasing concentration, however, the zinc salts hardly differ and are ranged between 6.4 and 6.7. The curves of pH_{eq} are almost parallel to each other. An exception were also the **iron** salt solutions. But for these the change in pH is very similar for all three salts. The pH_0 decreases with increasing c_0 of the solution. The curves of the respective pH_{eq} approach the curve of the pH_0 and correspond to the pH_0 from 500 mg L^{-1}. The starting pH_0 for $FeCl_2$ and $FeSO_4$ in the range of 4.3 - 5.5 differed markedly from the other salt solutions. After adsorption, there are almost no pH differences in these salt solutions. The pH_0 and pH_{eq} values of 2.2 for $Fe(NO_3)_3$ are extremely low in comparison to all other investigated metal salt solutions. The reason for this is that the iron is present in this salt as a trivalent ion and thus acts as a Lewis acid. Here, only at lower salt concentration, the pH_{eq} after adsorption changes significantly. At higher salt concentrations there are no significant pH differences.

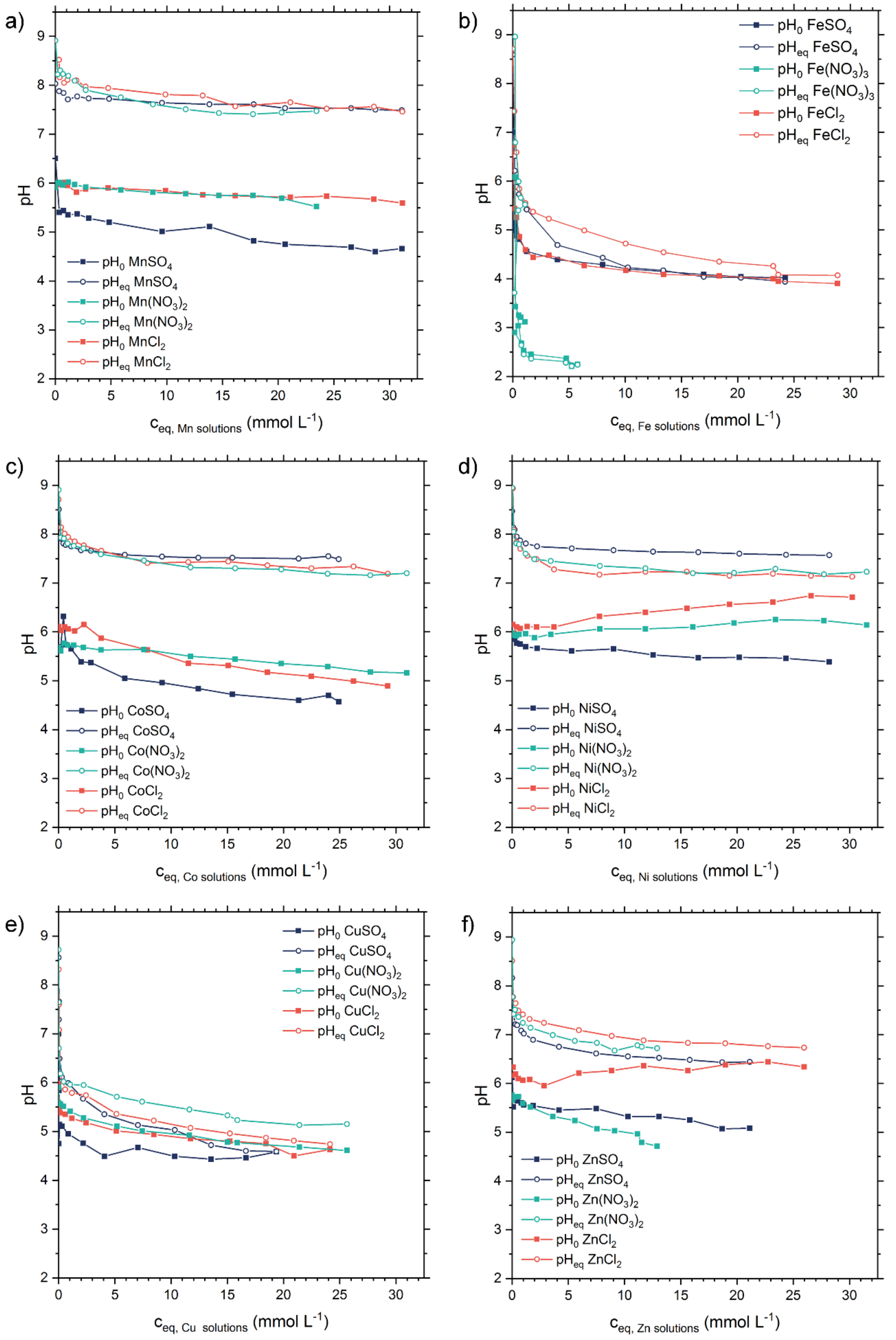

Figure S1: pH changes before (pH_0) and after (pH_{eq}) the adsorption process for a) manganese salts, b) iron salts, c) cobalt salts, d) nickel salts, e) copper salts, f) zinc salts in dependence of the anions (sulfate – blue, chloride – red, nitrate – green) and concentration at room temperature.

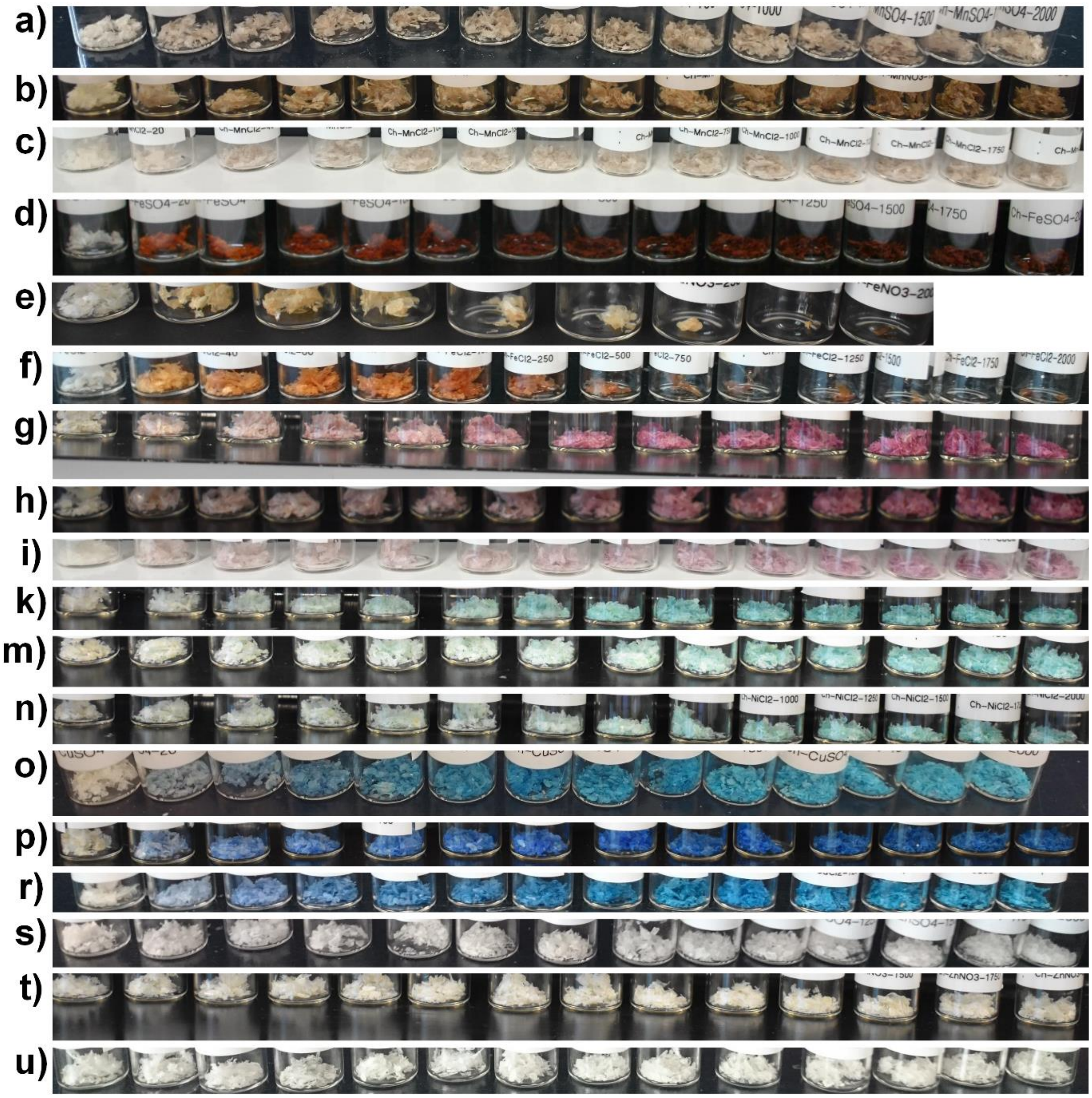

Figure S2: Images of a) $MnSO_4$, b) $Mn(NO_3)_2$ and c) $MnCl_2$, d) $FeSO_4$, e) $Fe(NO_3)_3$, f) $FeCl_2$, g) $CoSO_4$, h) $Co(NO_3)_2$, and i) $CoCl_2$, k) $NiSO_4$, m) $Ni(NO_3)_2$, and n) $NiCl_2$, o) $CuSO_4$, p) $Cu(NO_3)_2$, and r) $CuCl_2$, s) $ZnSO_4$, t) $Zn(NO_3)_2$, and u) $ZnCl_2$ after the adsorption process. Concentrations used from left-hand-side to right-hand-side: 0 mg L^{-1}, 20 mg L^{-1}, 40 mg L^{-1}, 60 mg L^{-1}, 100 mg L^{-1}, 150 mg L^{-1}, 250 mg L^{-1}, 500 mg L^{-1}, 750 mg L^{-1}, 1000 mg L^{-1}, 1250 mg L^{-1}, 1500 mg L^{-1}, 1750 mg L^{-1}, 2000 mg L^{-1} (initial metal ion concentration) and rinsed with water before drying after the adsorption process.

2.2 XRD Analysis

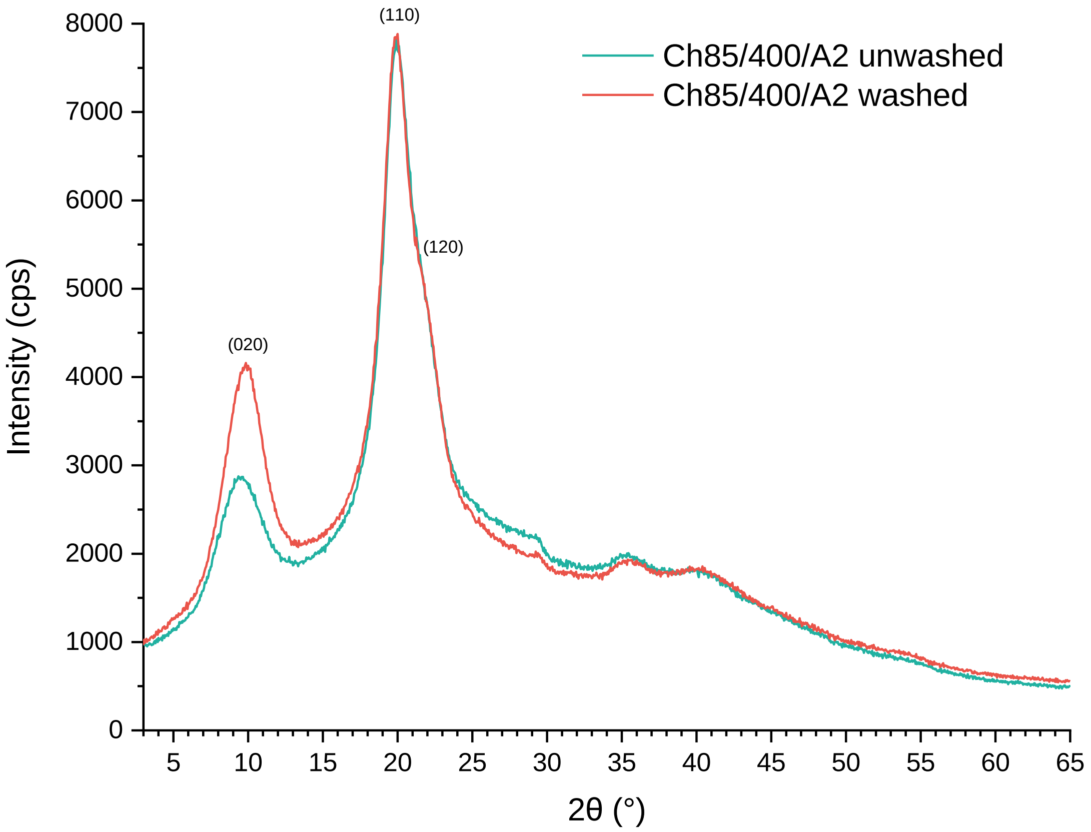

Figure S3: X-ray diffractograms of Ch85/400/A2 used as received (green line) and after rinsing the flakes with deionized water (red line).

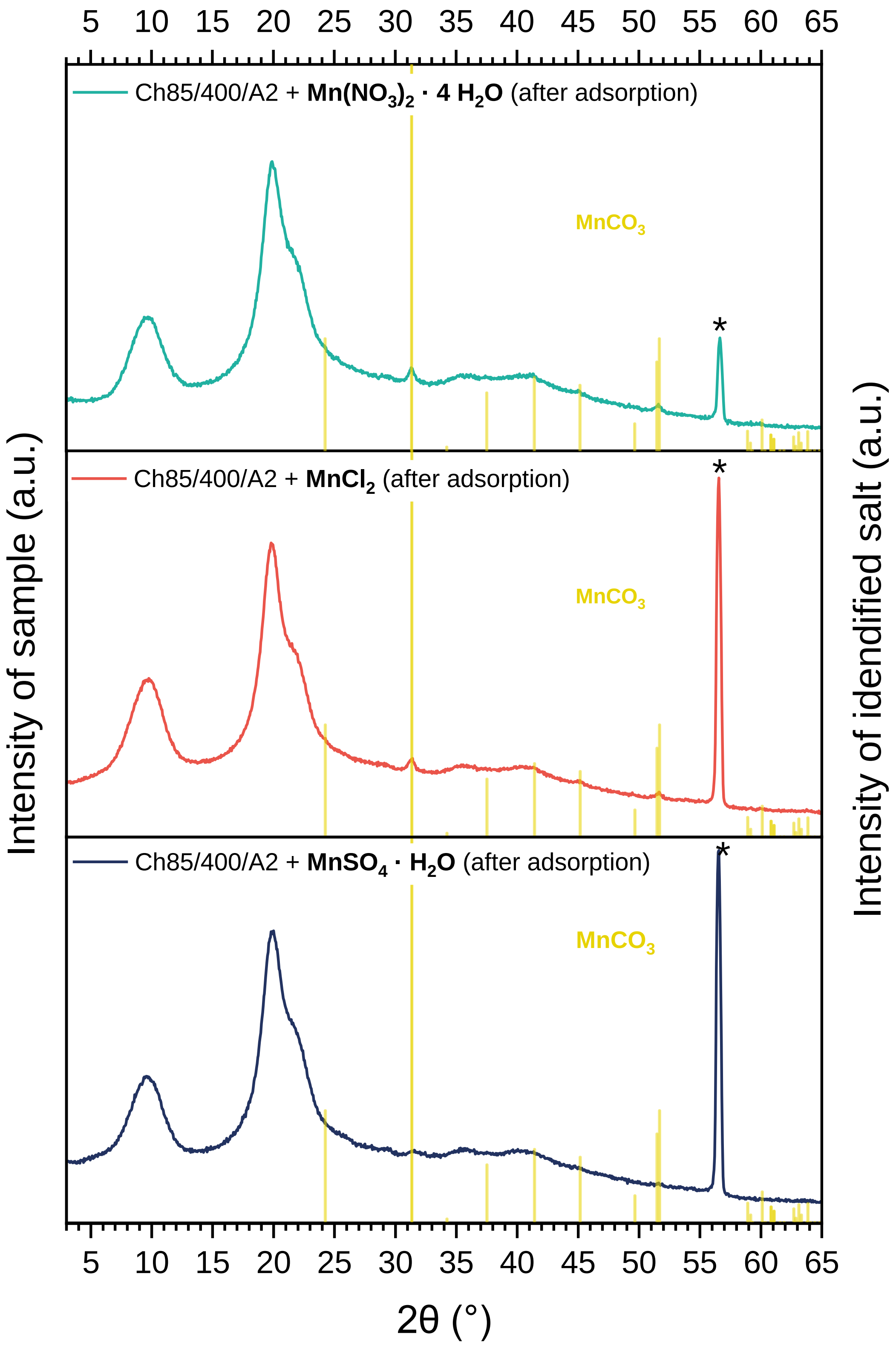

Figure S4: X-ray diffraction patterns of the chitosan flakes after the adsorption process with manganese nitrate c_0 = 5800 mg L^{-1} (green), manganese chloride c_0 = 4100 mg L^{-1} (red), and manganese sulfate c_0 = 5900 mg L^{-1} (blue). The pattern in yellow show the pure $MnCO_3$ for comparison. The samples were measured on a silicon wafer, which shows a reflection at 2θ 56.5° (labeled with a black star).

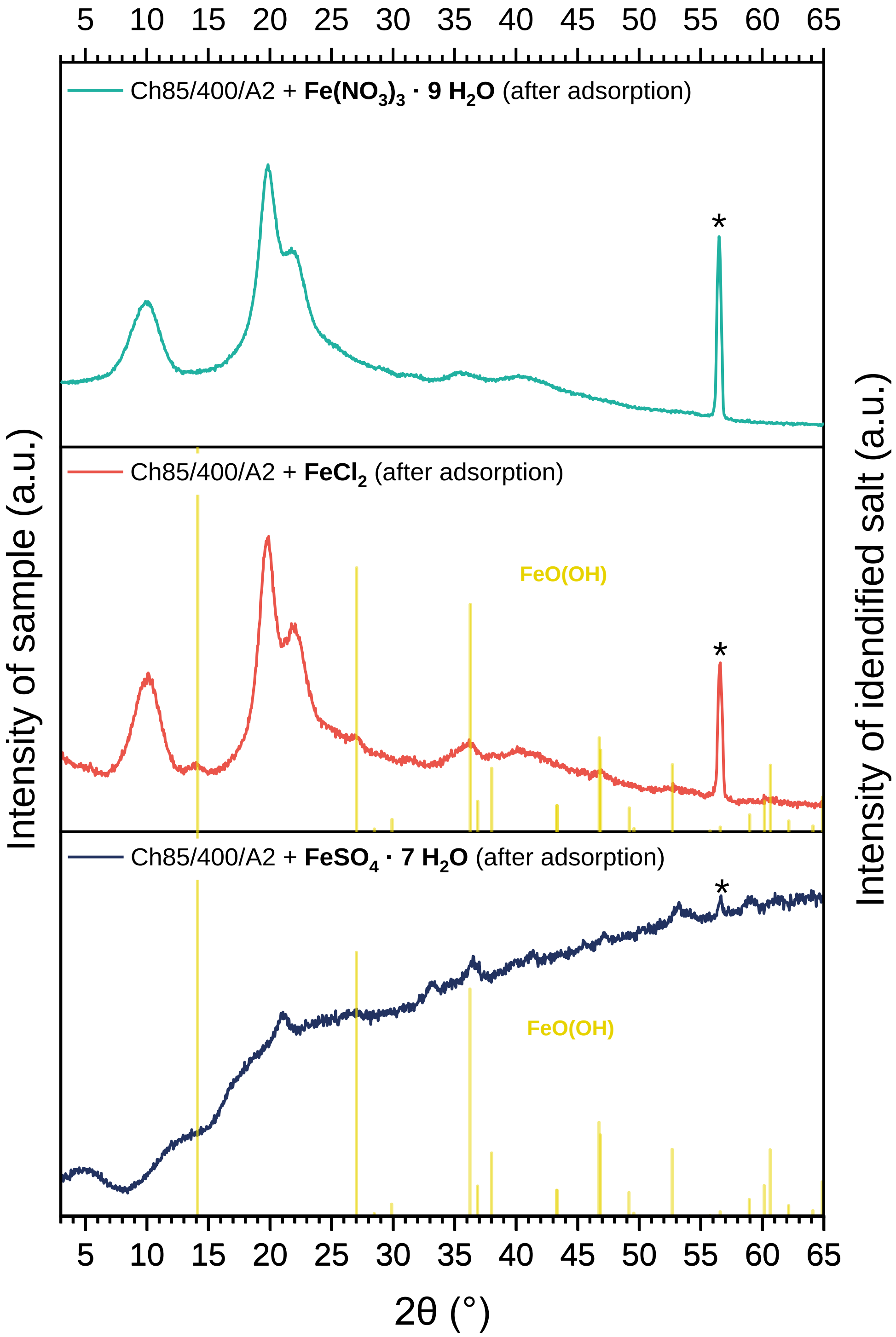

Figure S5: X-ray diffraction patterns of the chitosan flakes after the adsorption process with iron nitrate c_0 = 410 mg L^{-1} (green), iron chloride c_0 = 650 mg L^{-1} (red), and iron sulfate c_0 = 5900 mg L^{-1} (blue). The pattern in yellow show the pure FeO(OH) for comparison. The samples were measured on a silicon wafer, which shows a reflection at 2θ 56.5° (labeled with a black star).

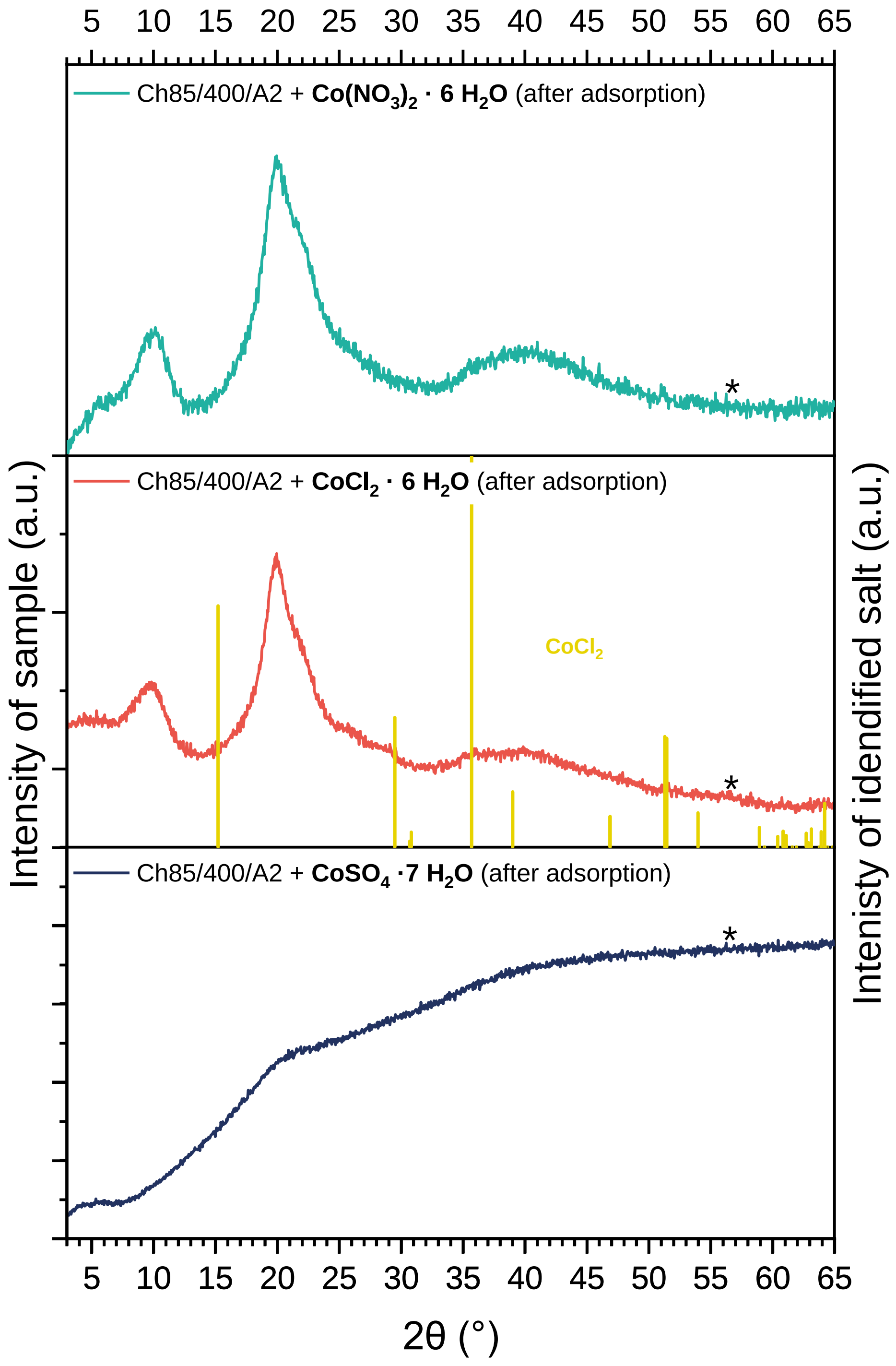

Figure S6: X-ray diffraction patterns of the chitosan flakes after the adsorption process with cobalt nitrate c_0 = 6200 mg L^{-1} (green), cobalt chloride c_0 = 4000 mg L^{-1} (red), and cobalt sulfate c_0 = 4800 mg L^{-1} (blue). The pattern in yellow shows the pure $CoCl_2$ for comparison. The samples were measured on a silicon wafer, which shows a reflection at 2θ 56.5° (labeled with a black star).

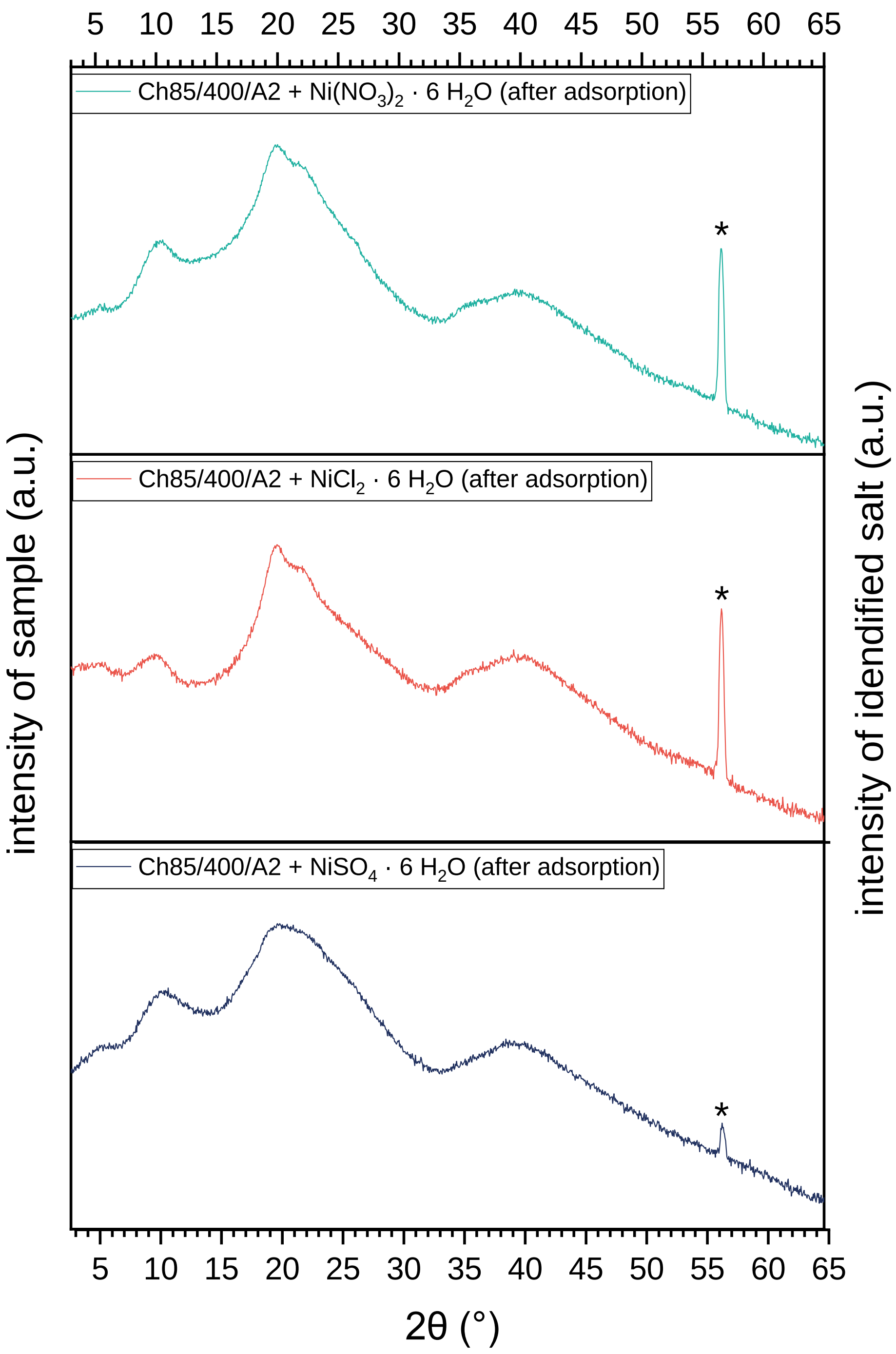

Figure S7: X-ray diffraction patterns of the chitosan flakes after the adsorption process with nickel nitrate c_0 = 6200 mg L^{-1} (green), nickel chloride c_0 = 4200 mg L^{-1} (red), and nickel sulfate c_0 = 5600 mg L^{-1} (blue). The samples were measured on a silicon wafer, which shows a reflection at 2θ 56.5° (labeled with a black star).

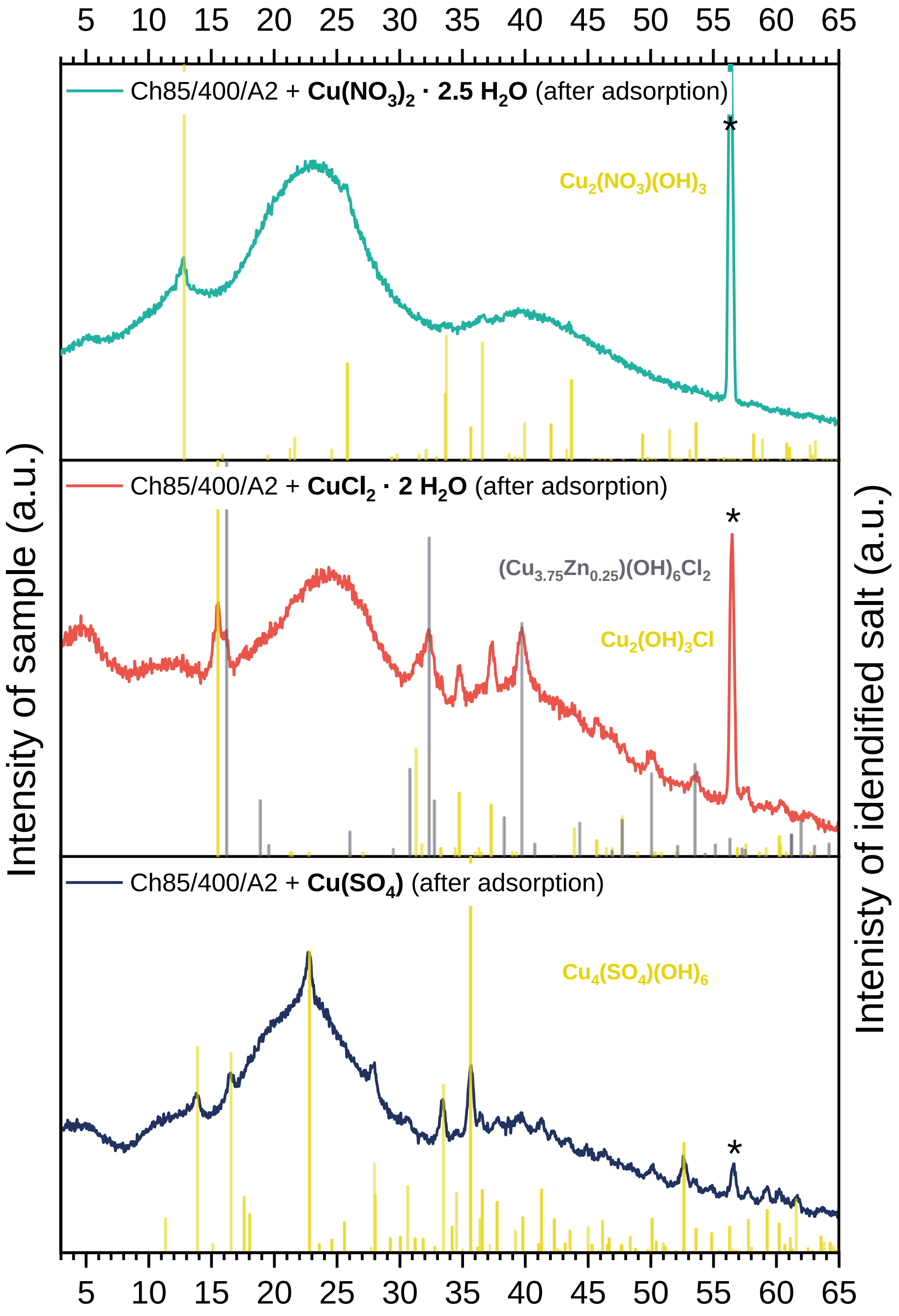

Figure S8: X-ray diffraction patterns of the chitosan flakes after the adsorption process with copper nitrate c_0 = 5800 mg L^{-1} (green), copper chloride c_0 = 4000 mg L^{-1} (red), and copper sulfate c_0 = 4700 mg L^{-1} (blue). The pattern in yellow shows the pure copper salts, which show a good match with the measured pattern. The samples were measured on a silicon wafer, which shows a reflection at 2θ 56.5° (labeled with a black star).

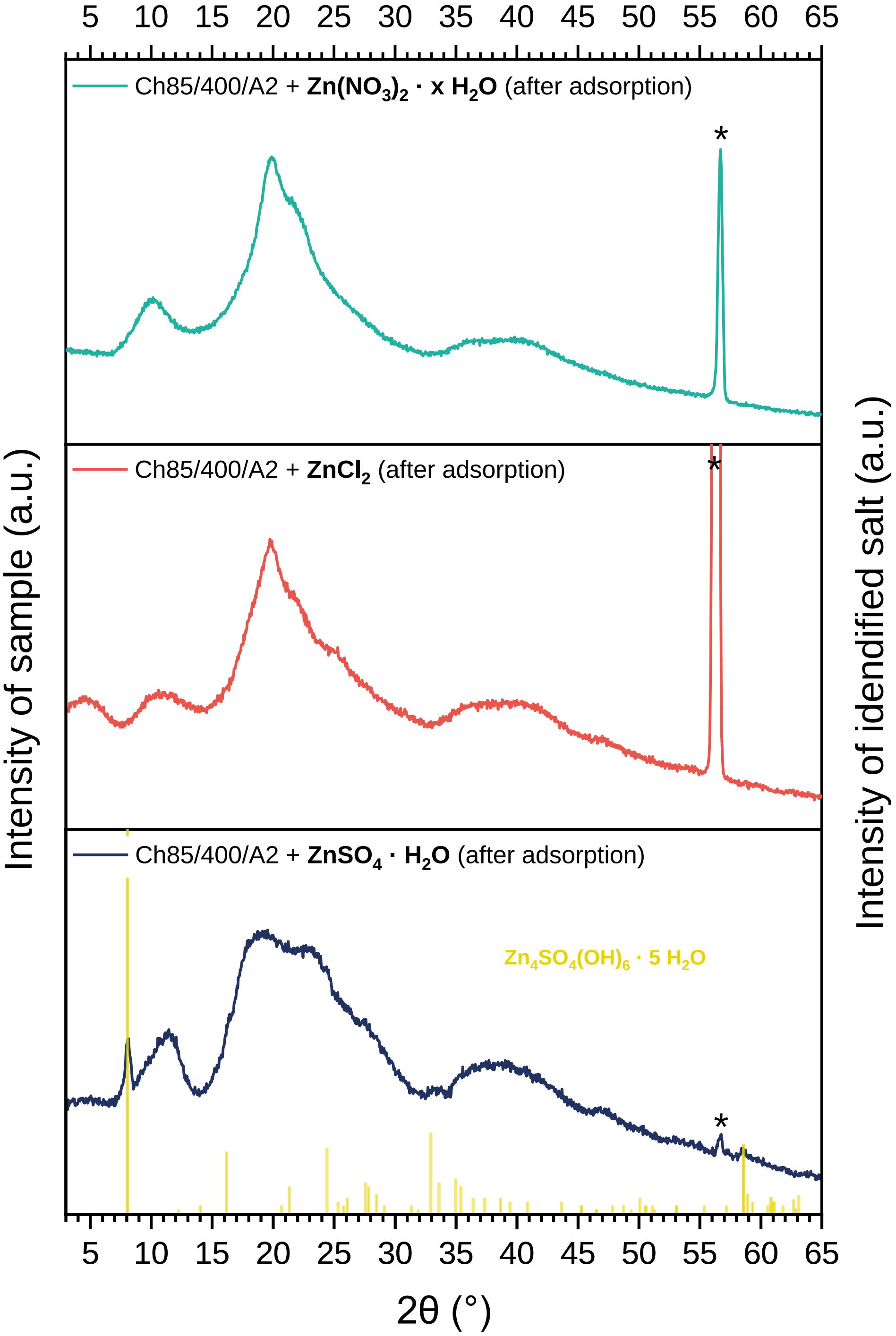

Figure S9: X-ray diffraction patterns of the chitosan flakes after the adsorption process with zinc nitrate c_0 = 3800 mg L^{-1} (green), zinc chloride c_0 = 4100 mg L^{-1} (red), and zinc sulfate c_0 = 5200 mg L^{-1} (blue). The pattern in yellow shows the pure zinc salt $Zn_4SO_4(OH)_6{\cdot}5H_2O$, which show a good match with the measured pattern. The samples were measured on a silicon wafer, which shows a reflection at 2θ 56.5° (labeled with a black star).

2.3 SEM Images

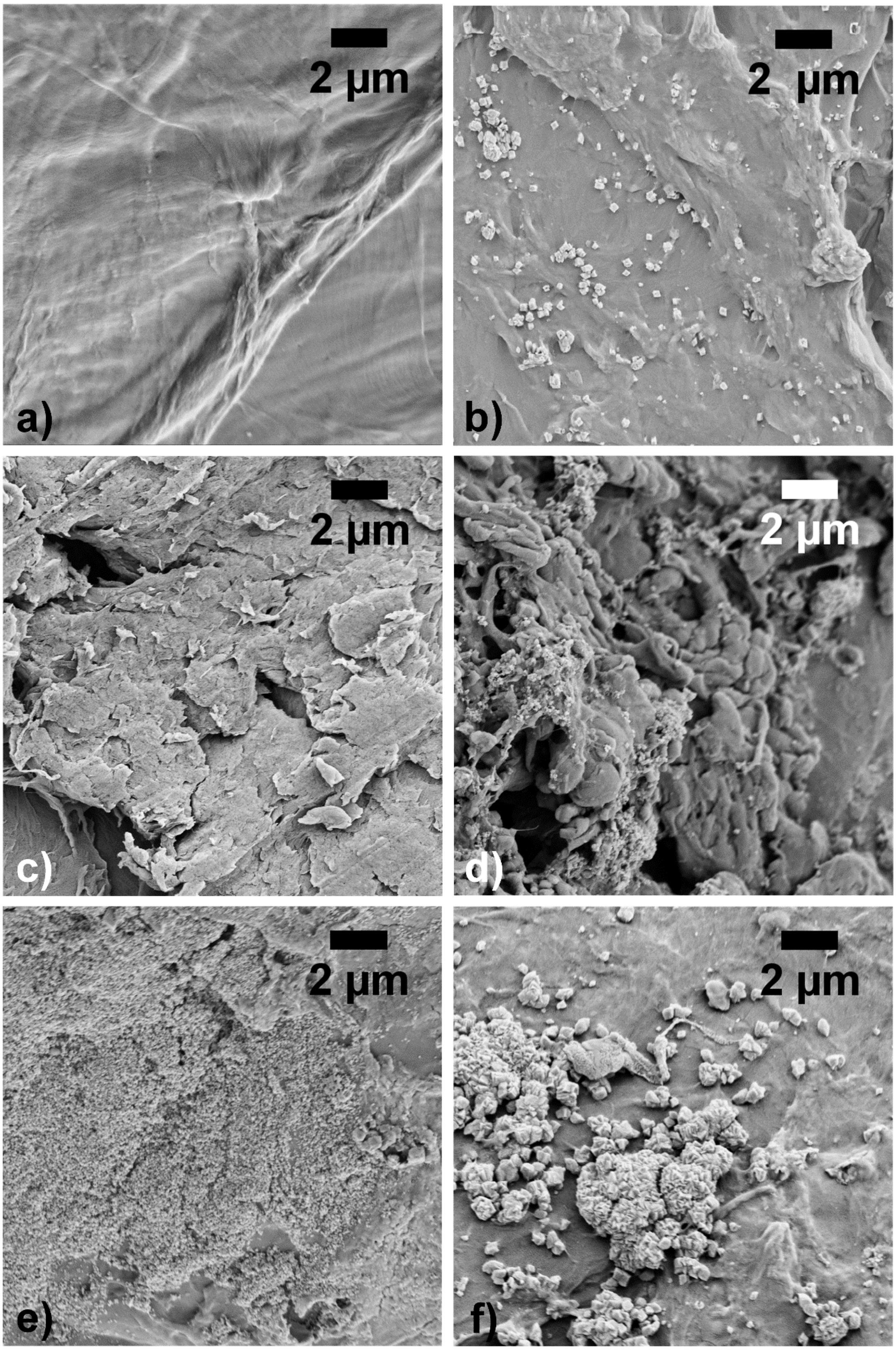

Figure S10: SEM images of manganese chloride a) c_0 = 50 mg L^{-1} and b) c_0 = 4000 mg L^{-1}; manganese nitrate c) c_0 = 60 mg L^{-1} and d) c_0 = 440 mg L^{-1}; manganese sulfate e) c_0 = 70 mg L^{-1} and f) c_0 = 6000 mg L^{-1}.

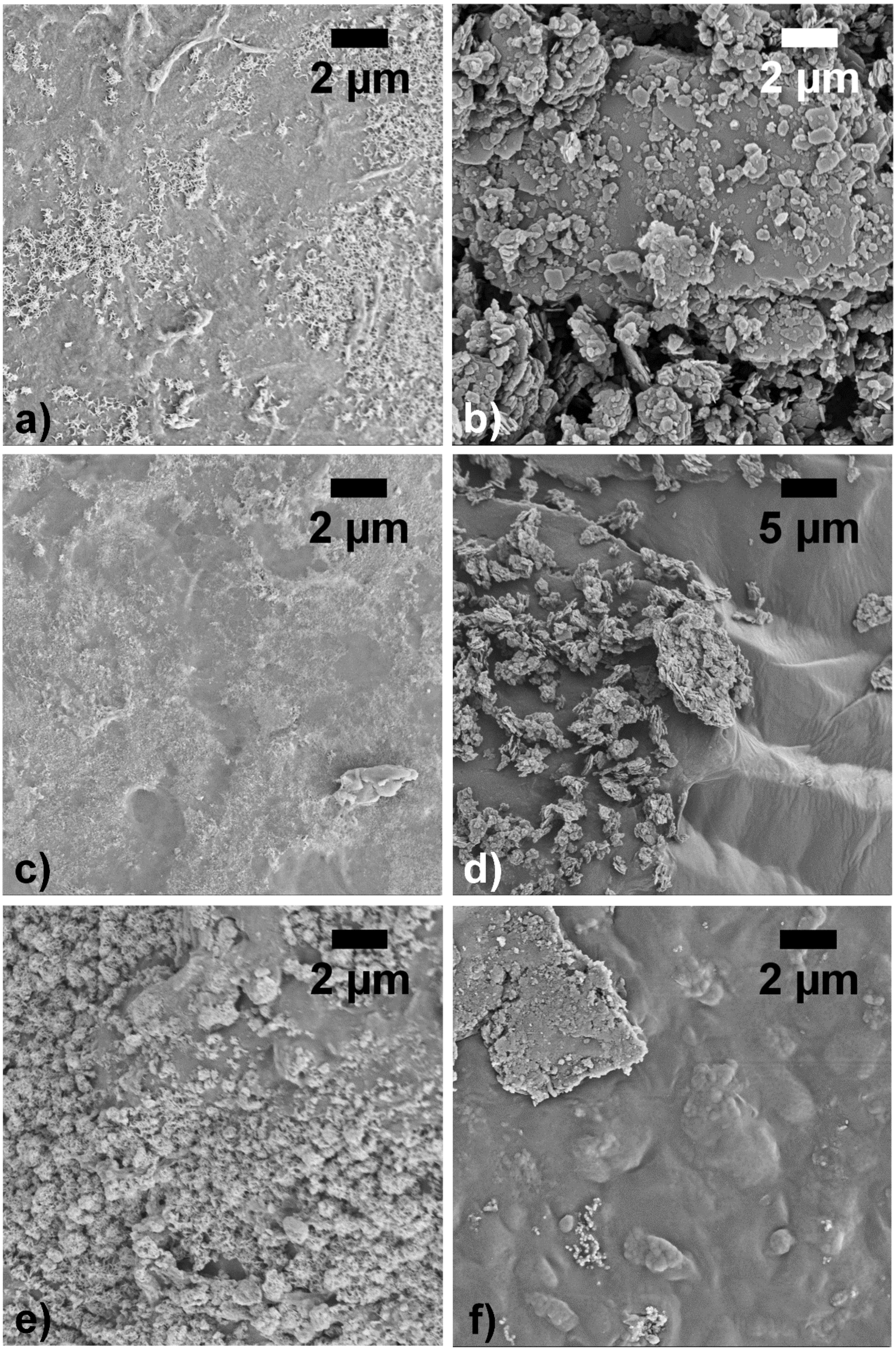

Figure S11: SEM images of iron chloride Iron chloride a) c_0 = 50 mg L^{-1} and b) c_0 = 4600 mg L^{-1}; iron nitrate c) c_0 = 90 mg L^{-1} and d) c_0 = 600 mg L^{-1}; iron sulfate e) c_0 = 80 mg L^{-1} and f) c_0 = 5900 mg L^{-1}.

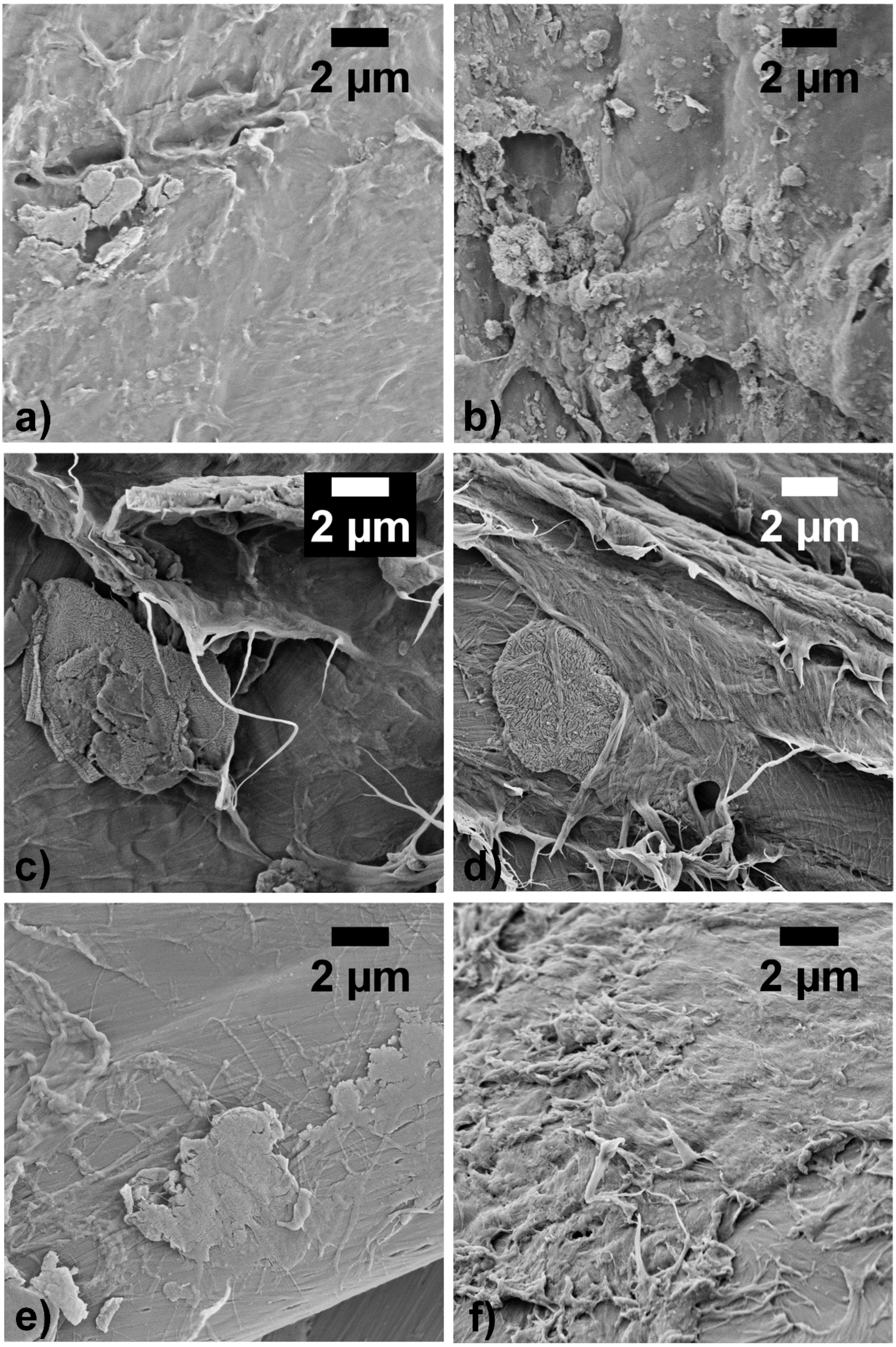

Figure S12: SEM images of cobalt chloride a) c_0 = 50 mg L^{-1} and b) c_0 = 4000 mg L^{-1}; cobalt nitrate c) c_0 = 60 mg L^{-1} and d) c_0 = 480 mg L^{-1}; cobalt sulfate e) c_0 = 50 mg L^{-1} and f) c_0 = 4800 mg L^{-1}.

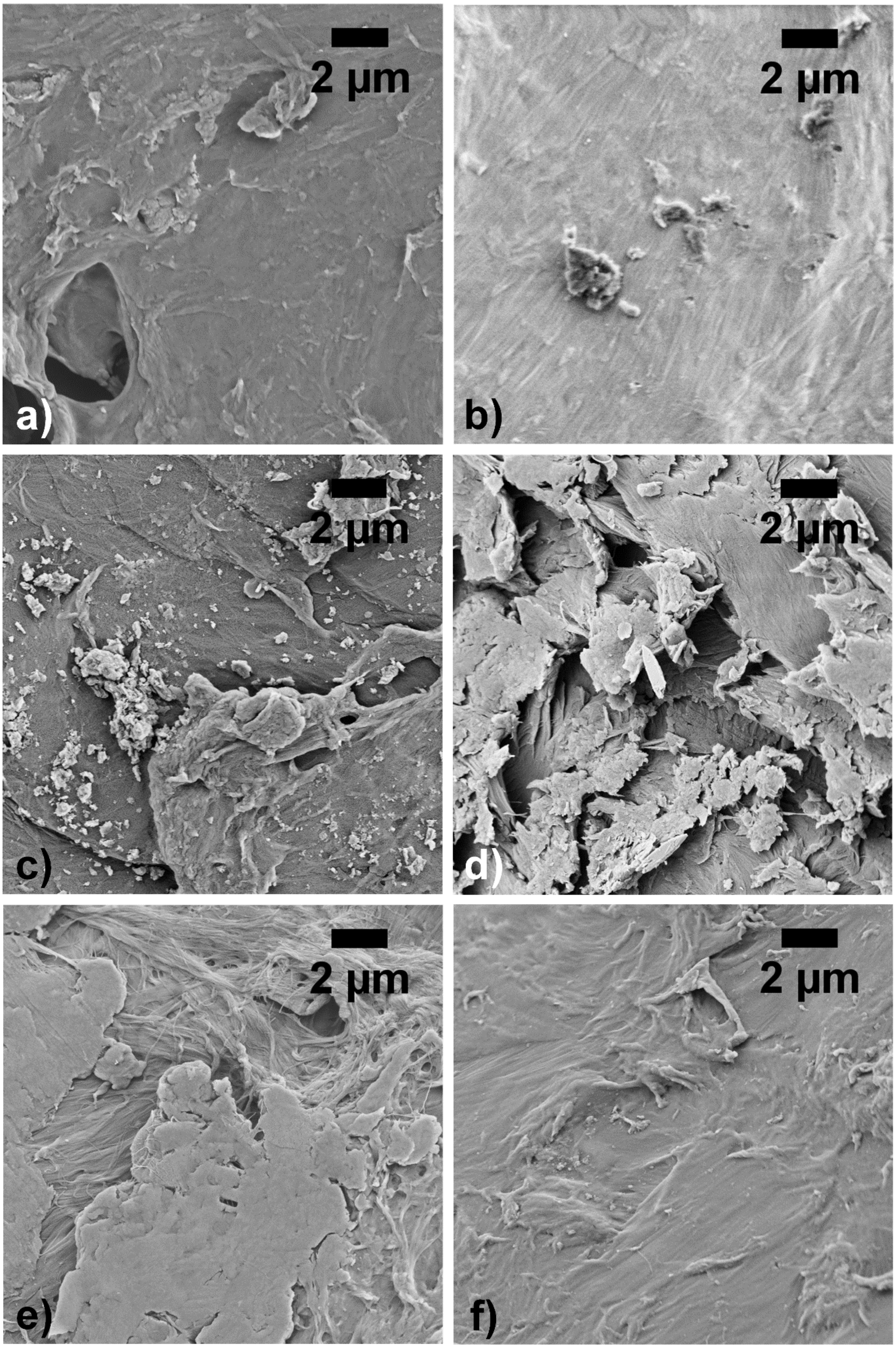

Figure S13: SEM images of nickel chloride a) c_0 = 50 mg L^{-1} and b) c_0 = 4200 mg L^{-1}; nickel nitrate c) c_0 = 60 mg L^{-1} and d) c_0 = 470 mg L^{-1}; nickel sulfate e) c_0 = 60 mg L^{-1} and f) c_0 = 5600 mg L^{-1}.

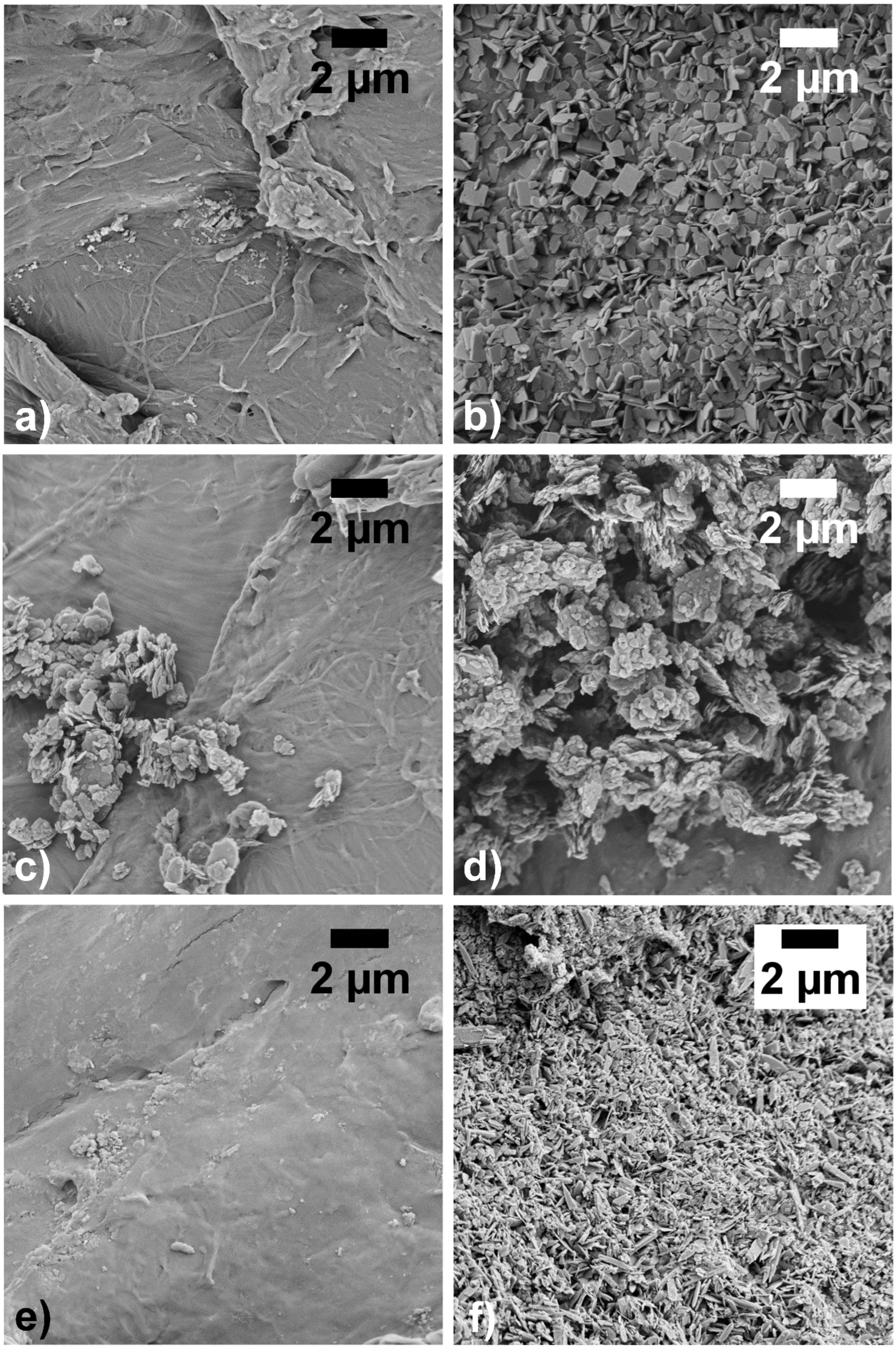

Figure S14: SEM images of copper chloride a) c_0 = 40 mg L^{-1} and b) c_0 = 4000 mg L^{-1}; copper nitrate c) c_0 = 50 mg L^{-1} and d) c_0 = 440 mg L^{-1}; copper sulfate e) c_0 = 50 mg L^{-1} and f) c_0 = 4700 mg L^{-1}.

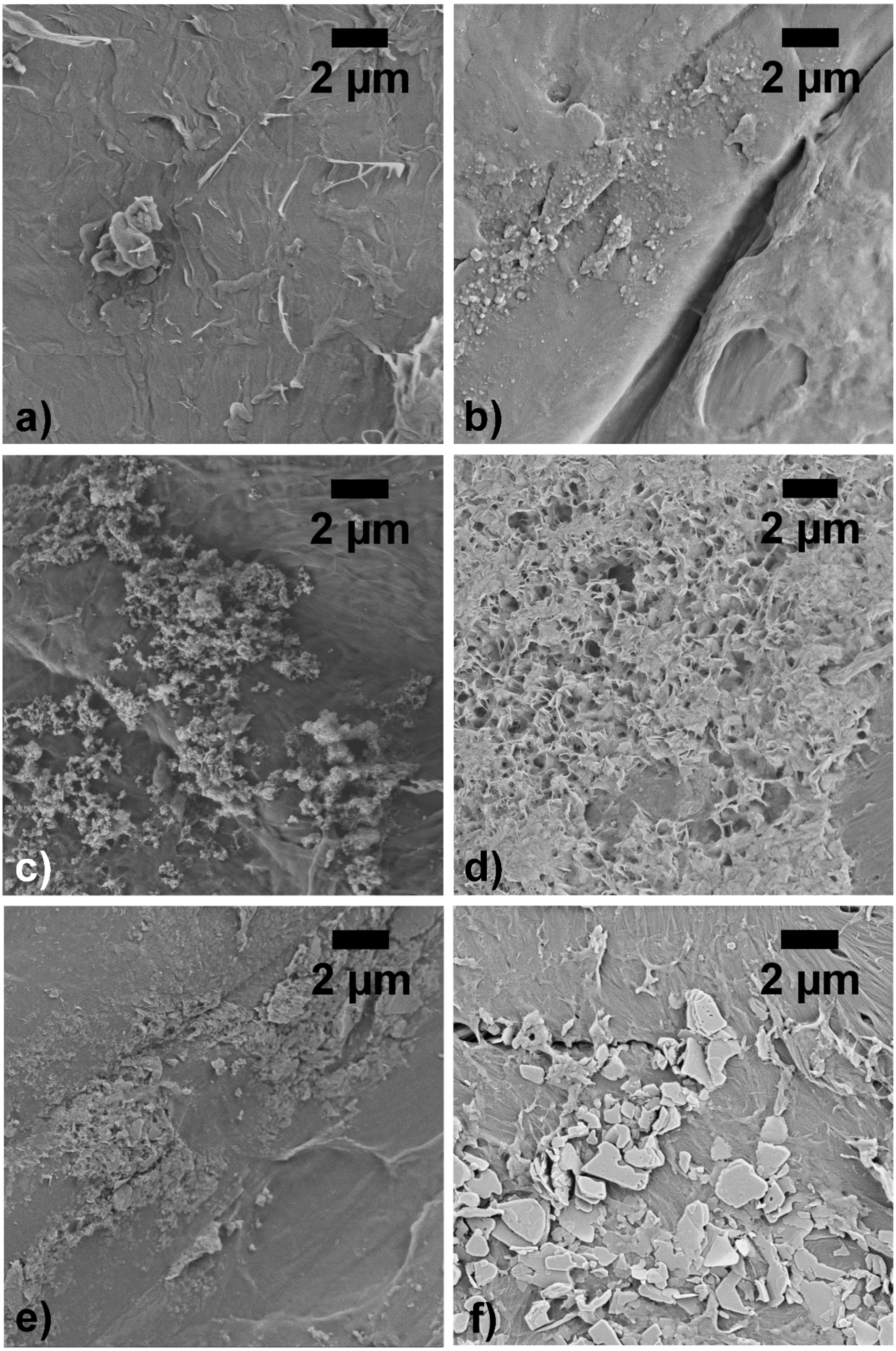

Figure S15: SEM images of zinc chloride a) c_0 = 40 mg L^{-1} and b) c_0 = 4100 mg L^{-1}; zinc nitrate c) c_0 = 30 mg L^{-1} and d) c_0 = 260 mg L^{-1}; zinc sulfate e) c_0 = 60 mg L^{-1} and f) c_0 = 5200 mg L^{-1}.

2.4 EDX-Analysis

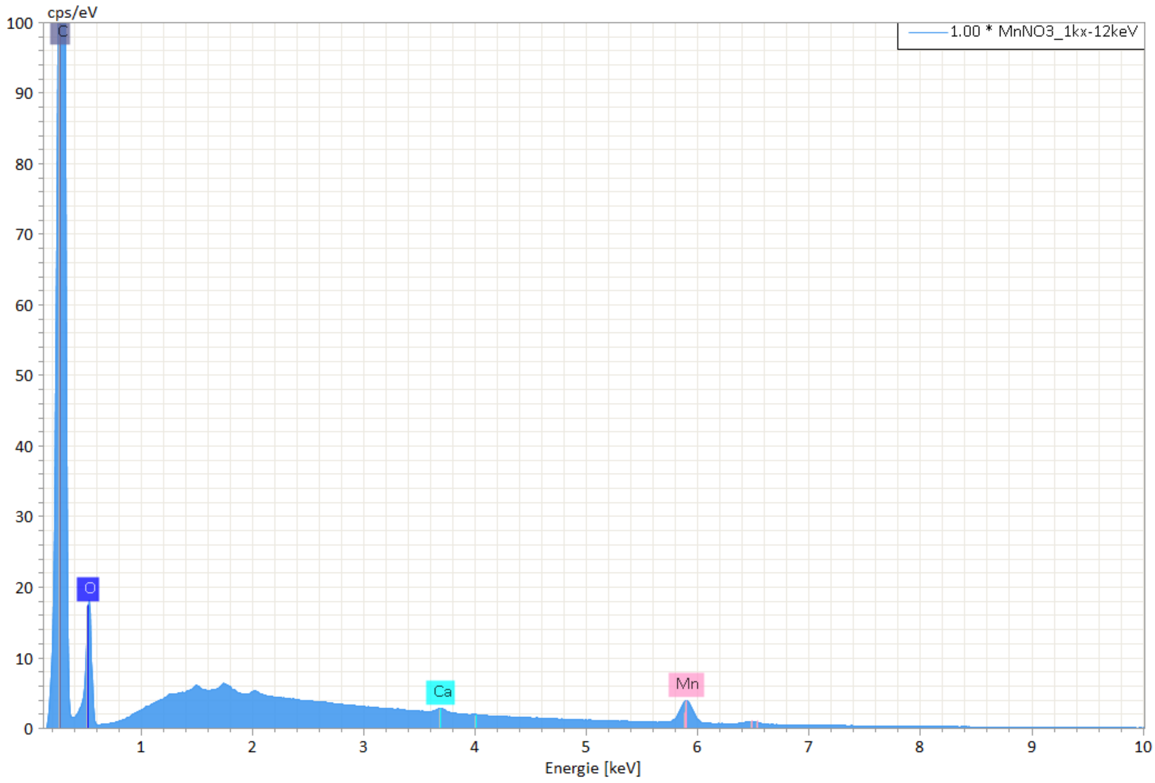

Figure S16: EDX pattern of Ch-Mn(NO_3)$_2$ (c_0 = 5800 mg L^{-1}) after the adsorption process.

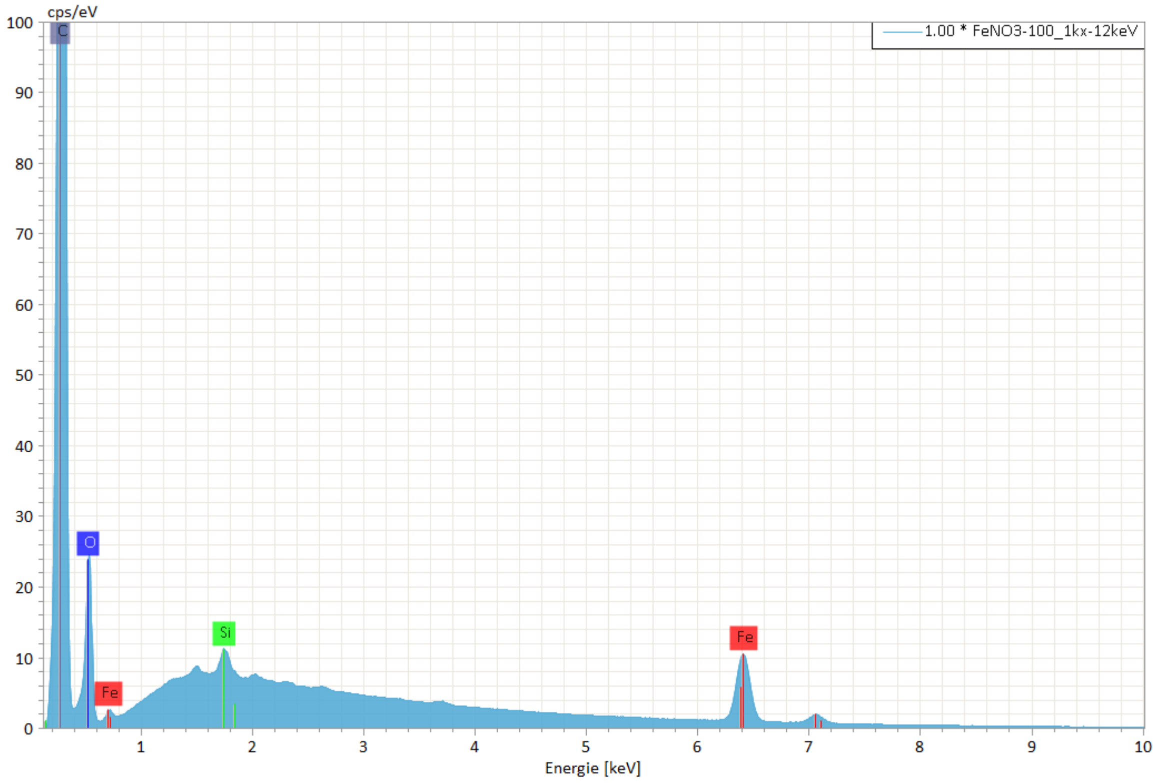

Figure S17: EDX pattern of Ch-Fe(NO_3)$_2$ (c_0 = 410 mg L^{-1}) after the adsorption process.

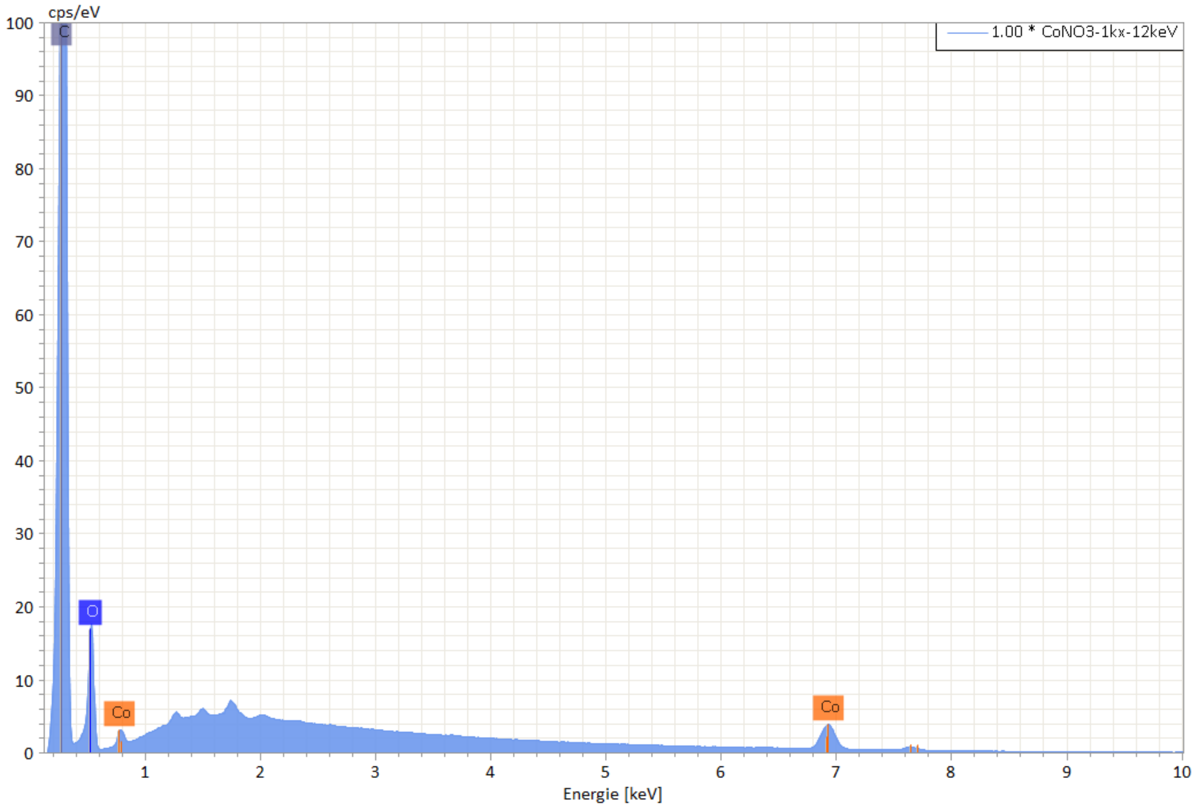

Figure S18: EDX pattern of Ch-Co(NO_3)$_2$ (c_0 = 6200 mg L^{-1}) after the adsorption process.

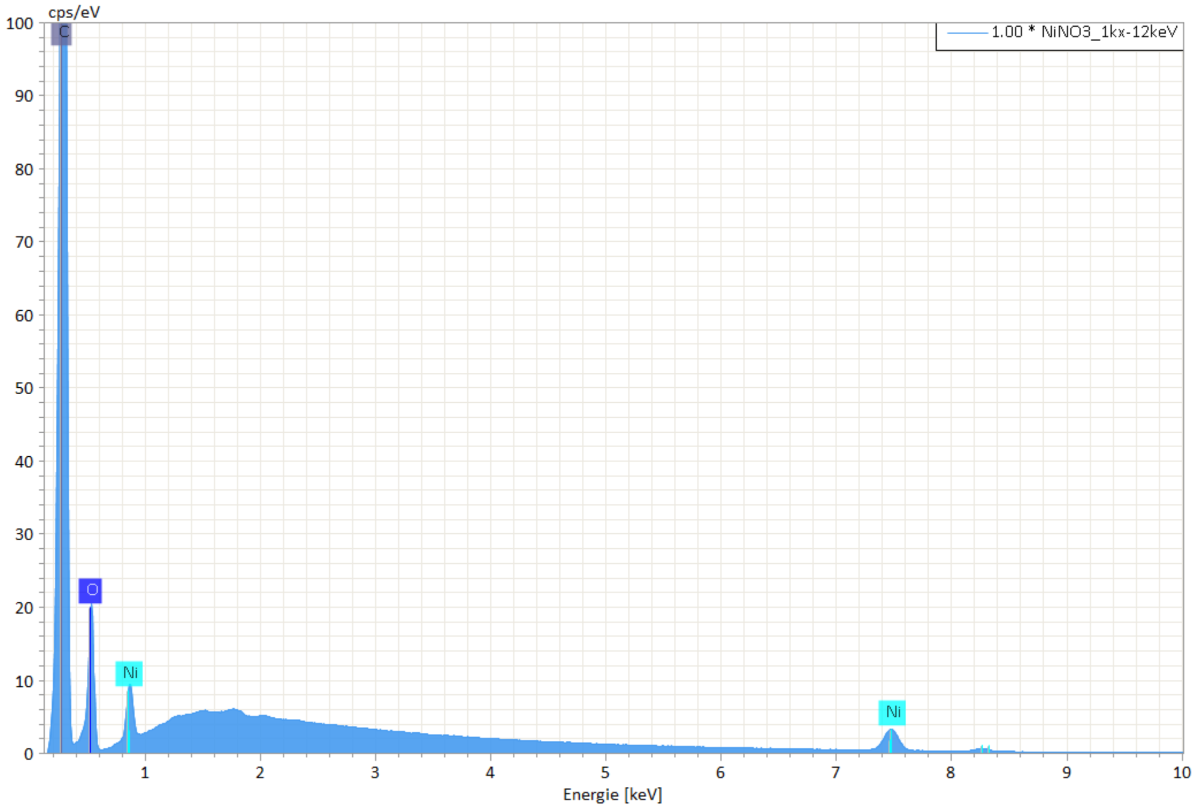

Figure S19: EDX pattern of Ch-Ni(NO_3)$_2$ (c_0 = 6200 mg L^{-1}) after the adsorption process.

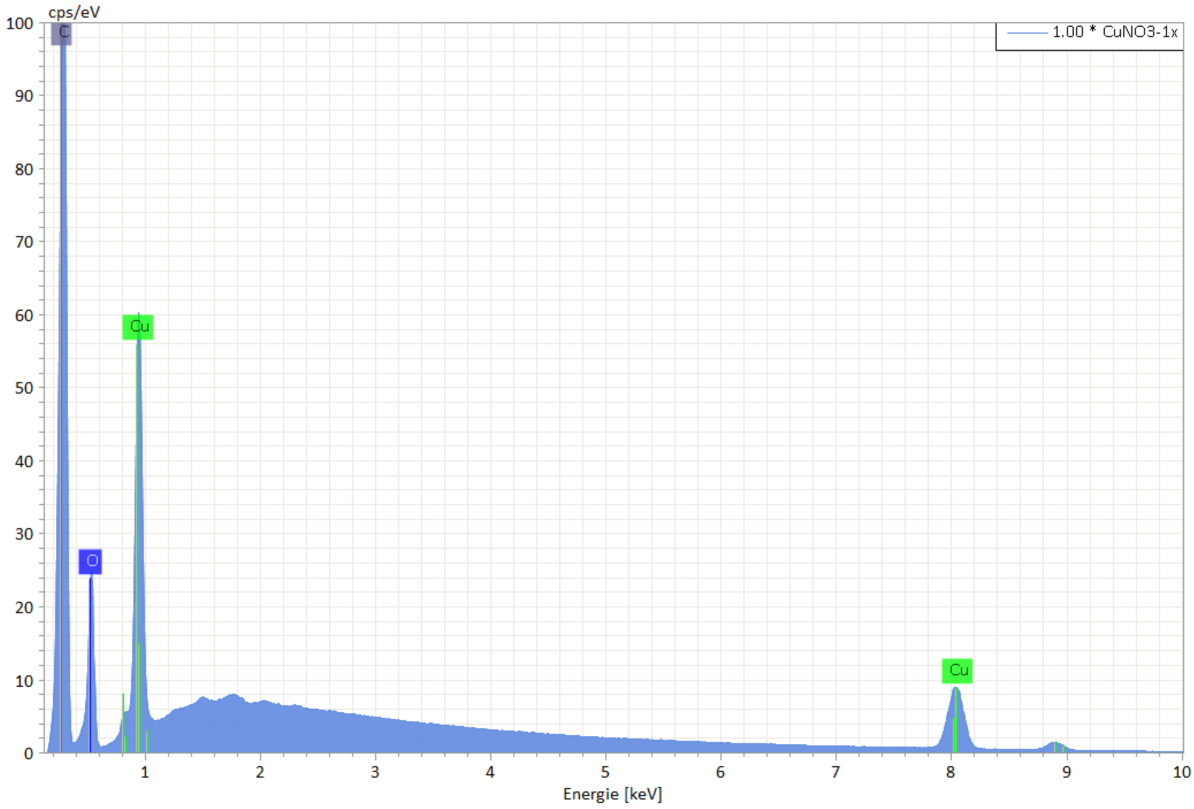

Figure S20: EDX pattern of Ch-$Cu(NO_3)_2$ (c_0 = 5800 mg L^{-1}) after the adsorption process.

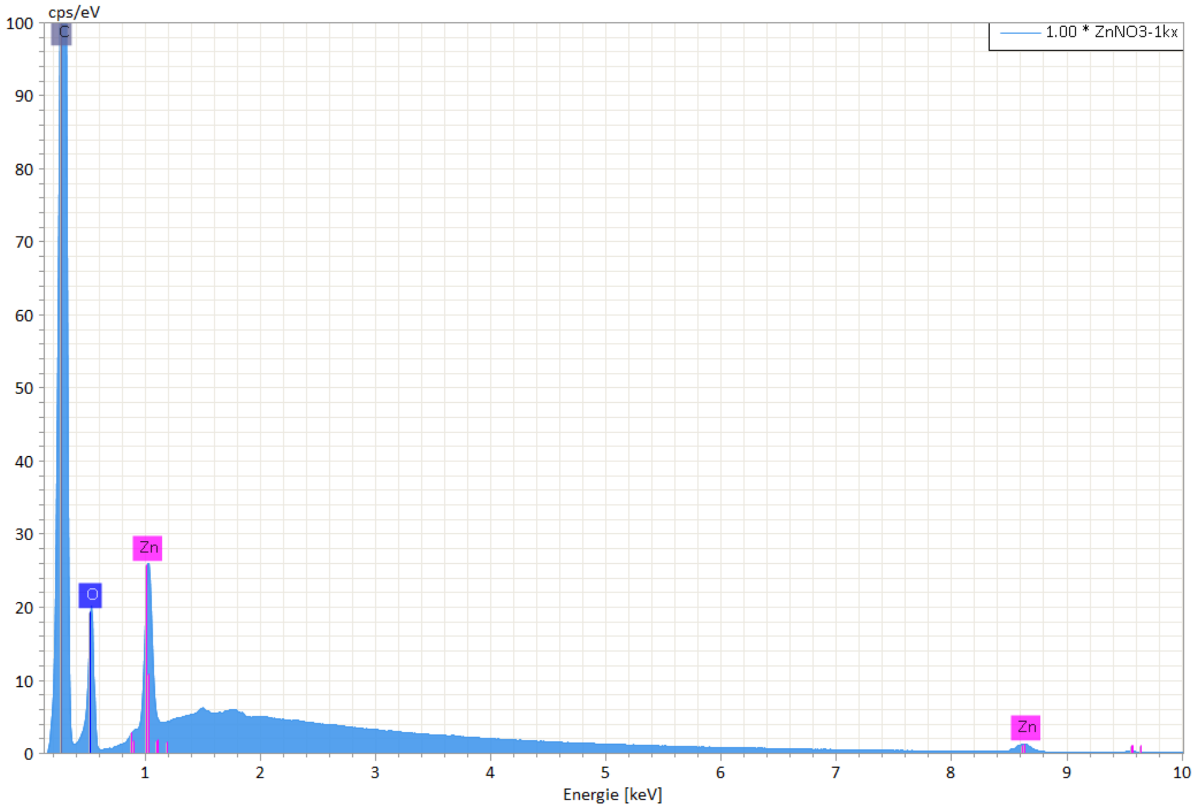

Figure S21: EDX pattern of Ch-$Zn(NO_3)_2$ (c_0 = 3800 mg L^{-1}) after the adsorption process.

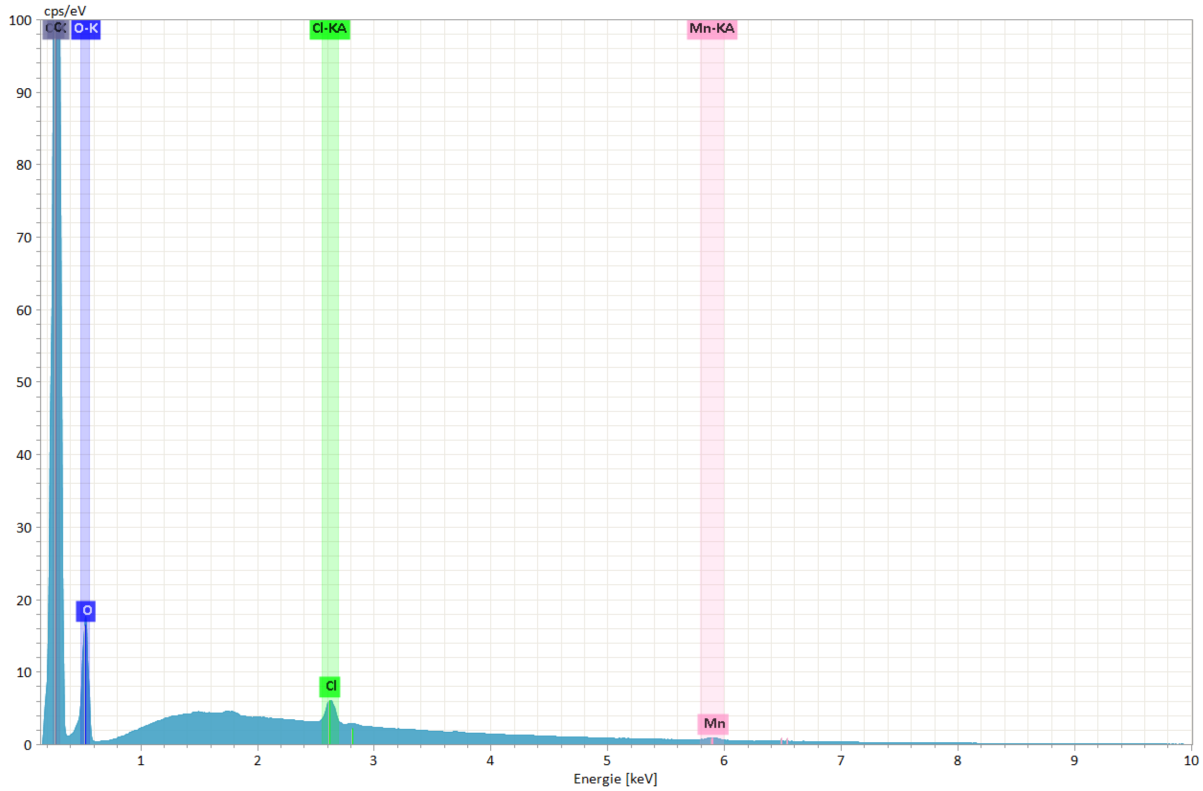

Figure S22: EDX pattern of Ch-$MnCl_2$ (c_0 = 4100 mg L^{-1}) after the adsorption process.

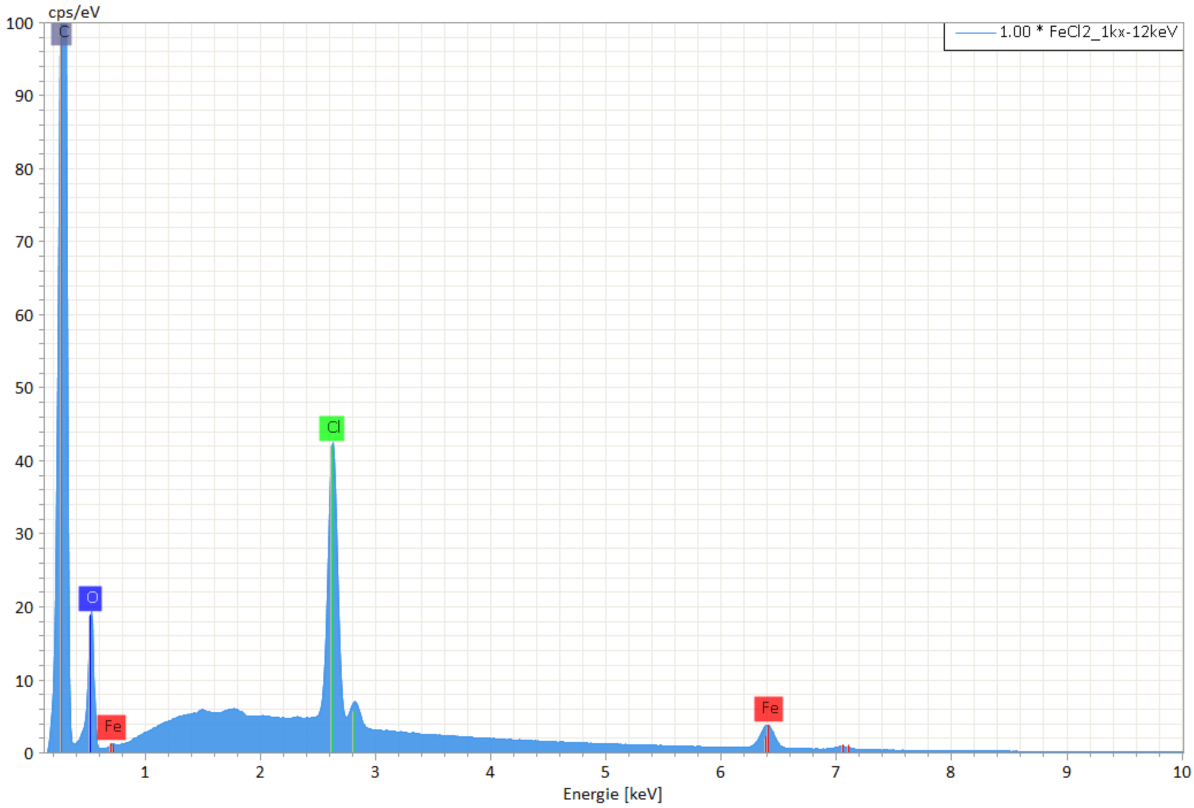

Figure S23: EDX pattern of Ch-$FeCl_2$ (c_0 = 650 mg L^{-1}) after the adsorption process.

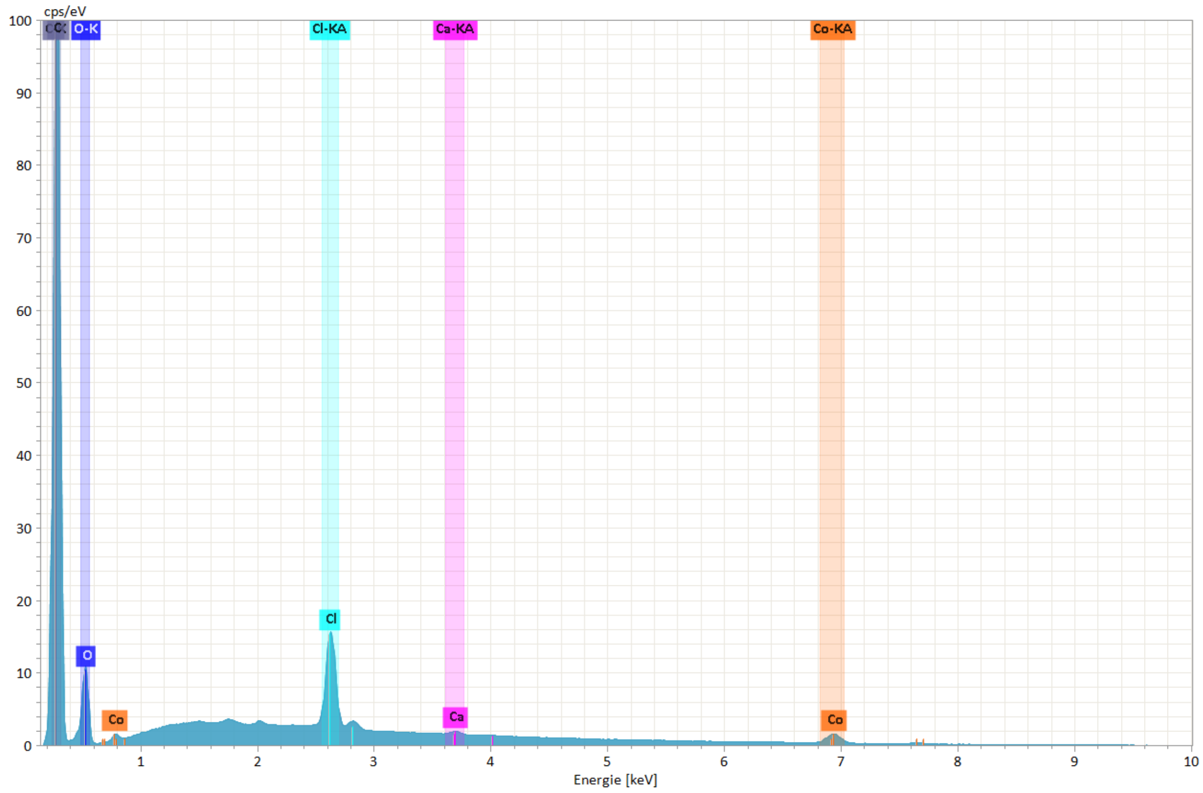

Figure S24: EDX pattern of Ch-$CoCl_2$ (c_0 = 4000 mg L^{-1}) after the adsorption process.

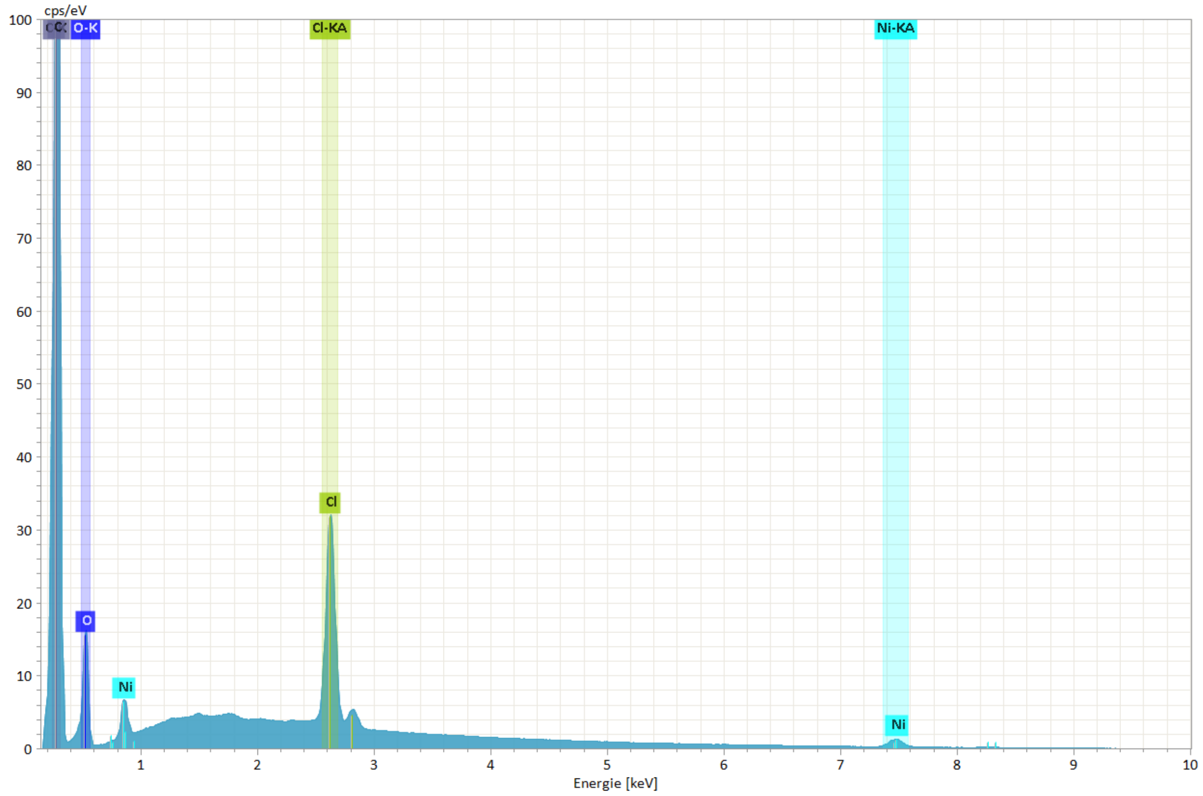

Figure S25: EDX pattern of Ch-$NiCl_2$ (c_0 = 4200 mg L^{-1}) after the adsorption process.

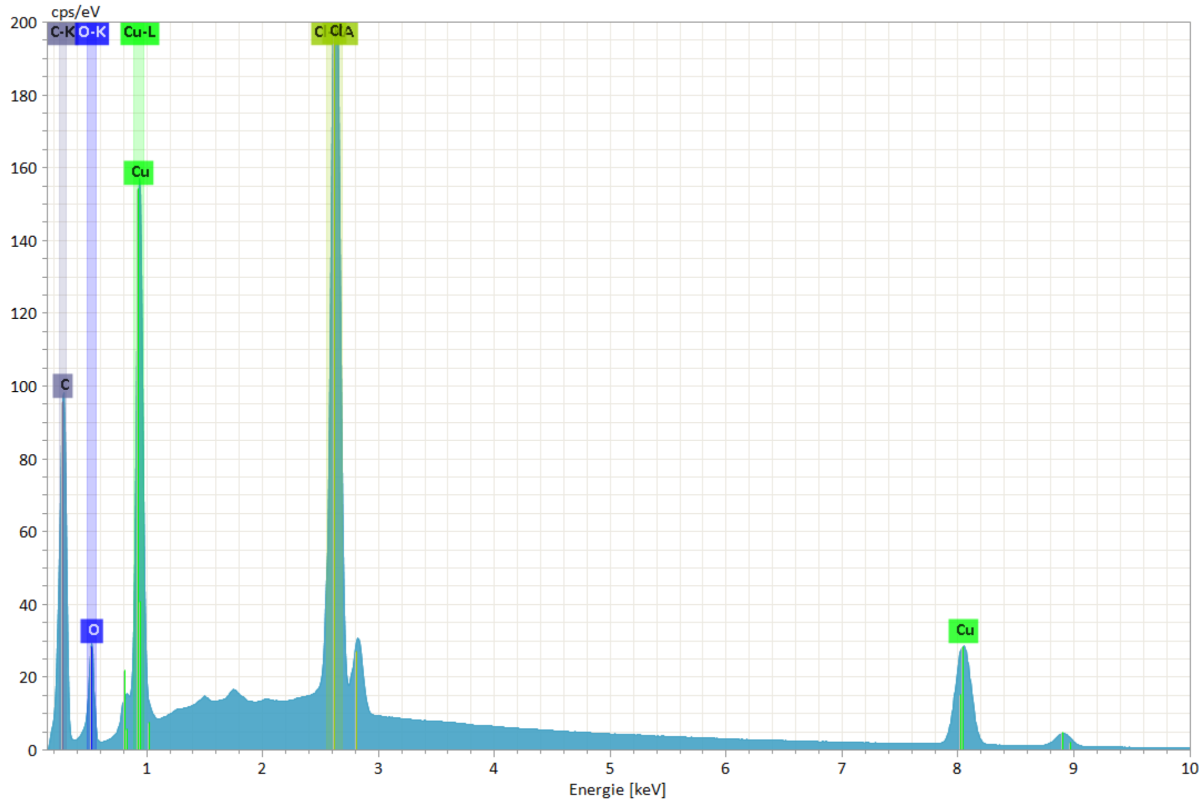

Figure S26: EDX pattern of Ch-$CuCl_2$ (c_0 = 4000 mg L^{-1}) after the adsorption process.

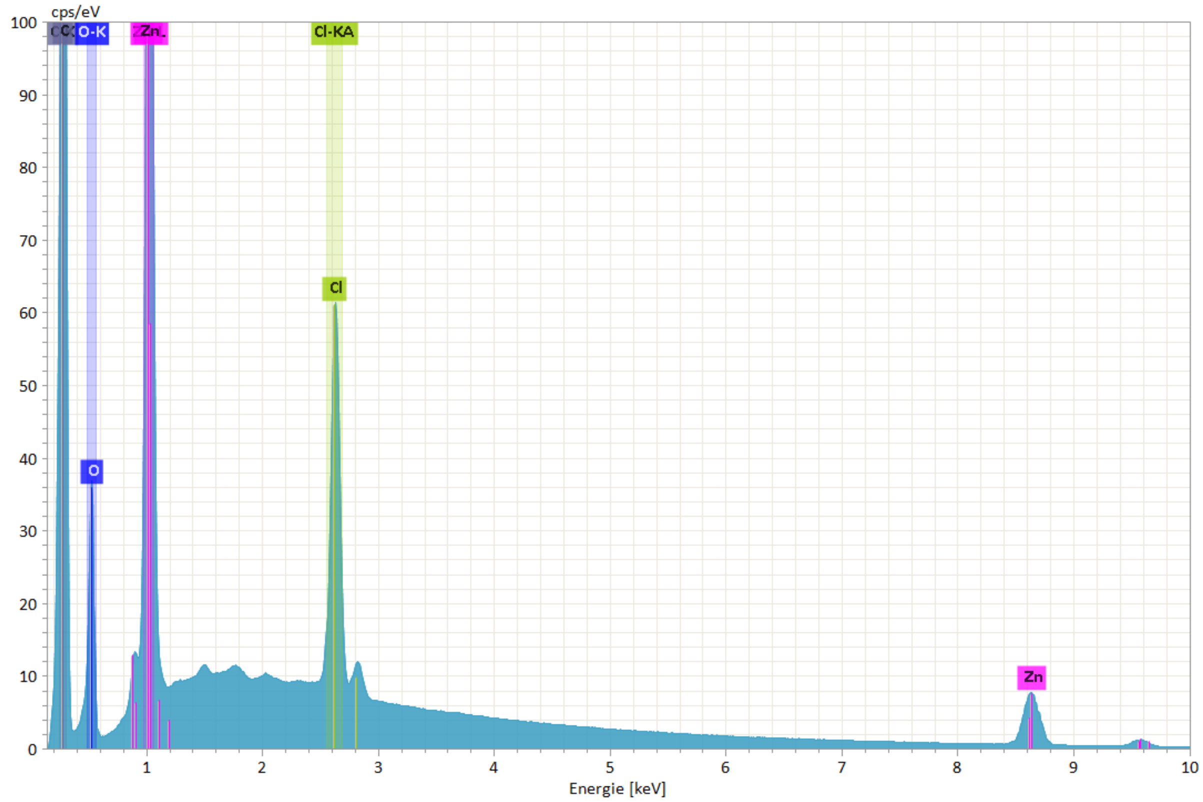

Figure S27: EDX pattern of Ch-$ZnCl_2$ (c_0 = 4100 mg L^{-1}) after the adsorption process.

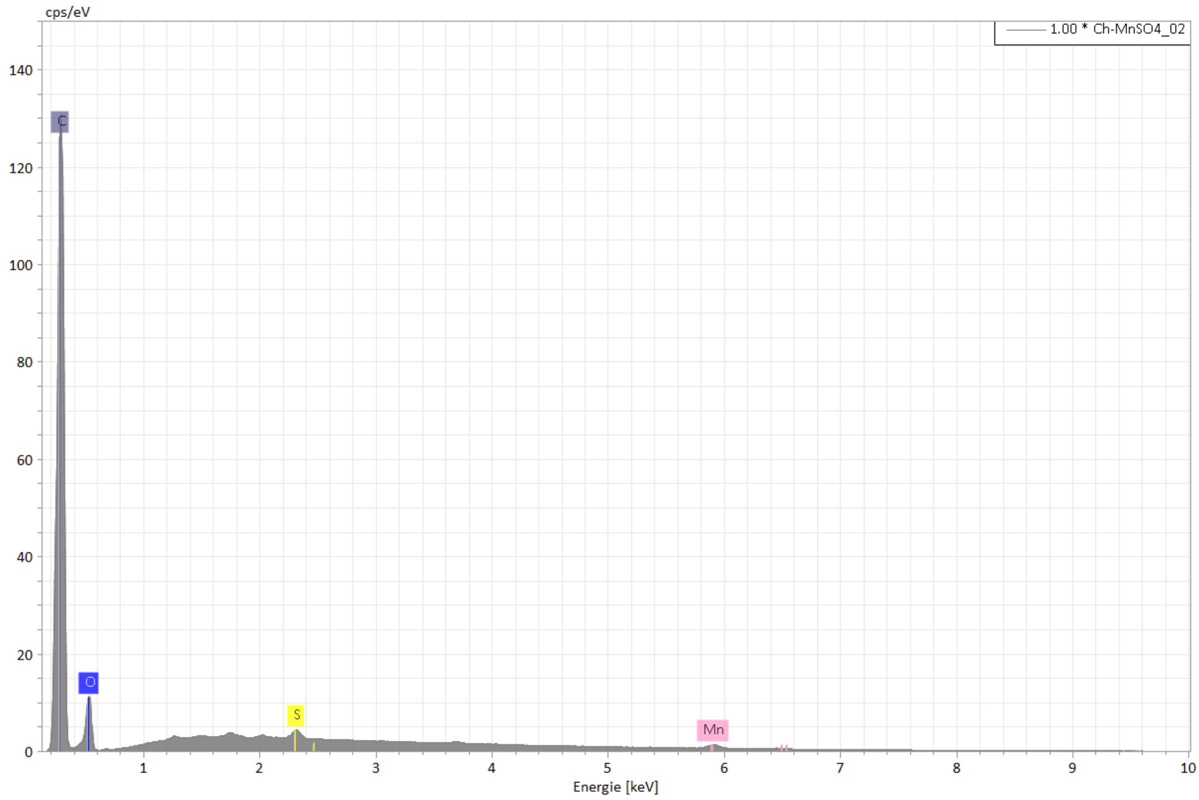

Figure S28: EDX pattern of Ch-$MnSO_4$ (c_0 = 5900 mg L^{-1}) after the adsorption process.

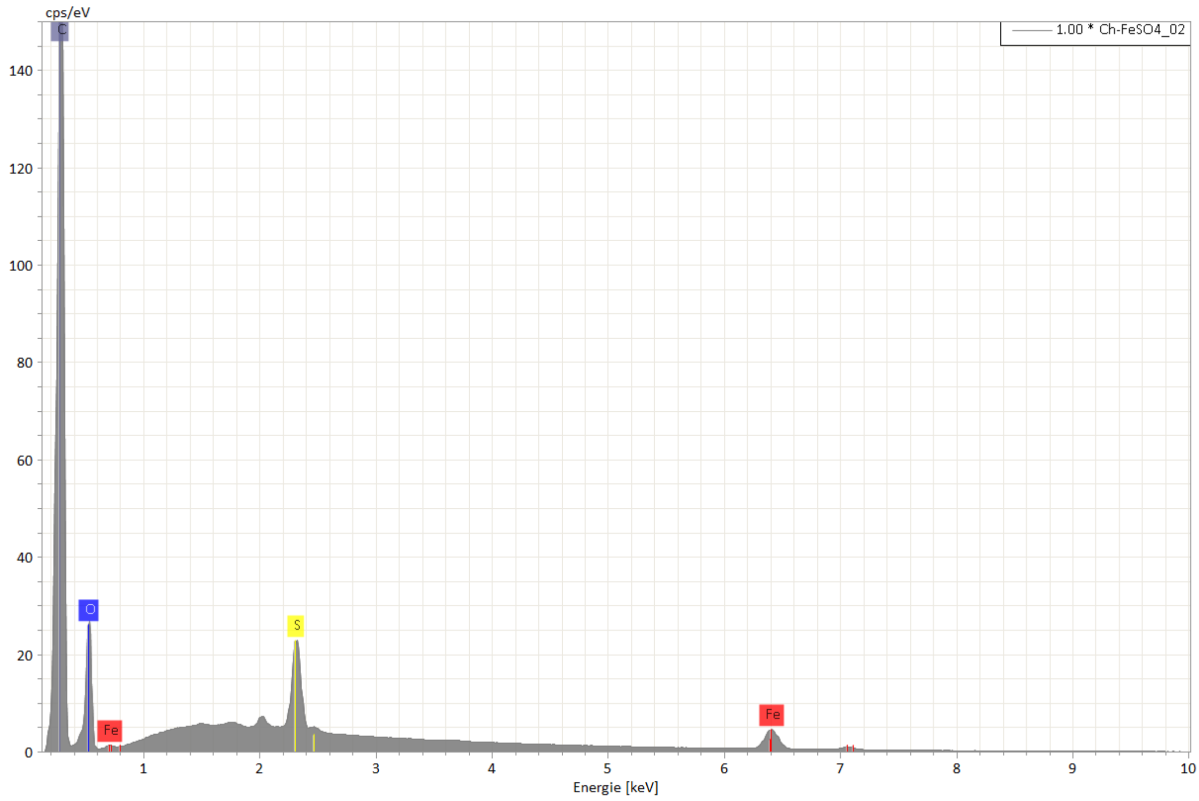

Figure S29: EDX pattern of Ch-$FeSO_4$ (c_0 = 5900 mg L^{-1}) after the adsorption process.

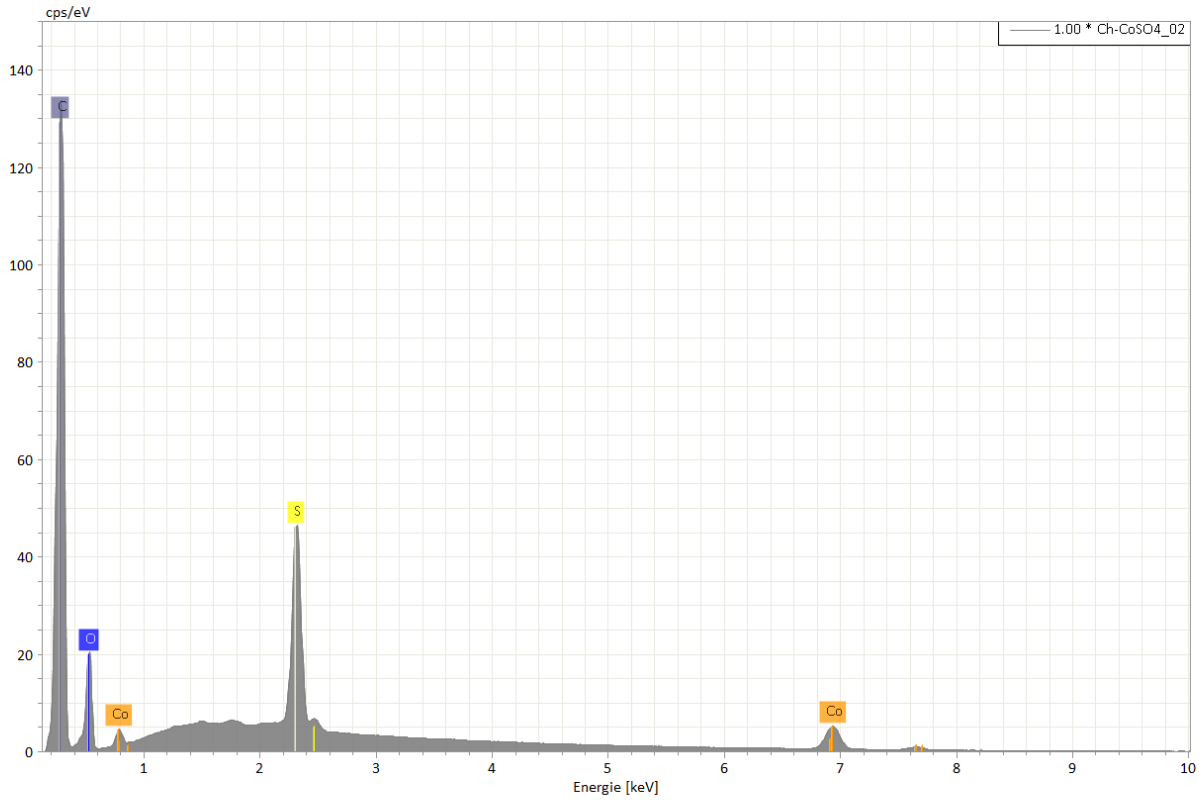

Figure S30: EDX pattern of Ch-$CoSO_4$ (c_0 = 4800 mg L^{-1}) after the adsorption process.

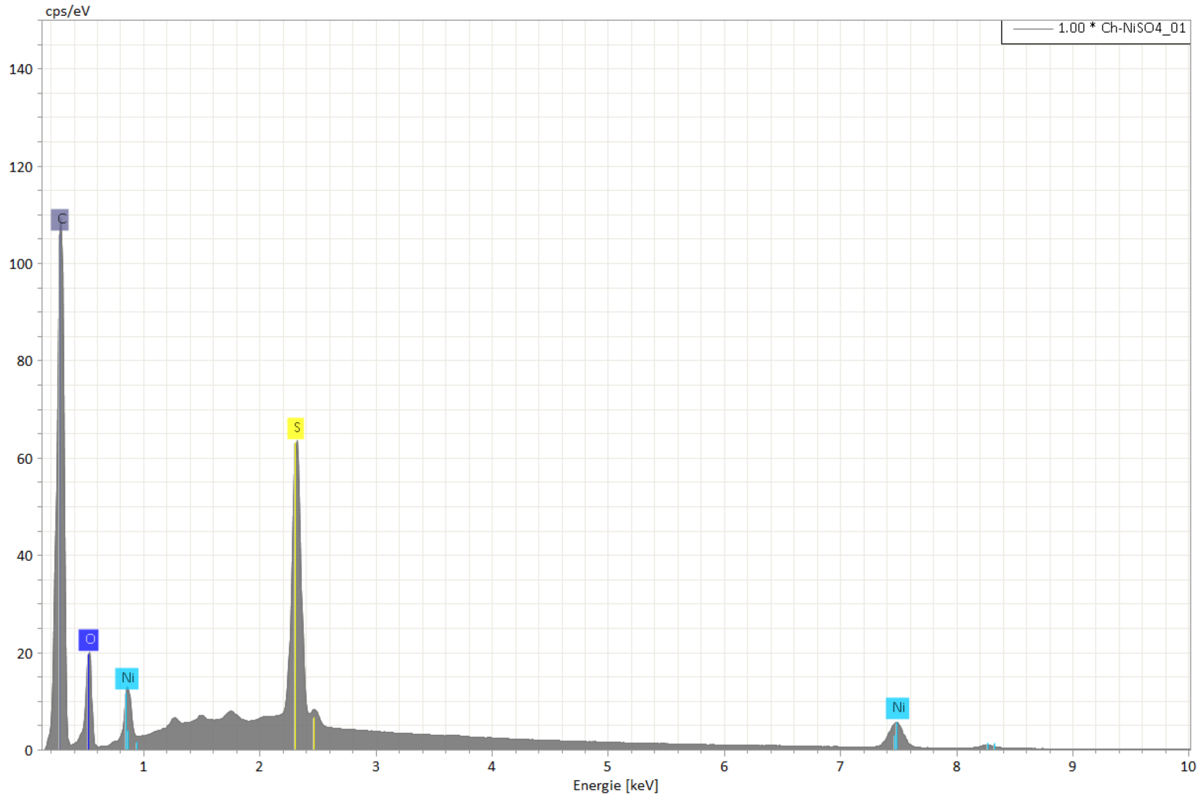

Figure S31: EDX pattern of Ch-$NiSO_4$ (c_0 = 5600 mg L^{-1}) after the adsorption process.

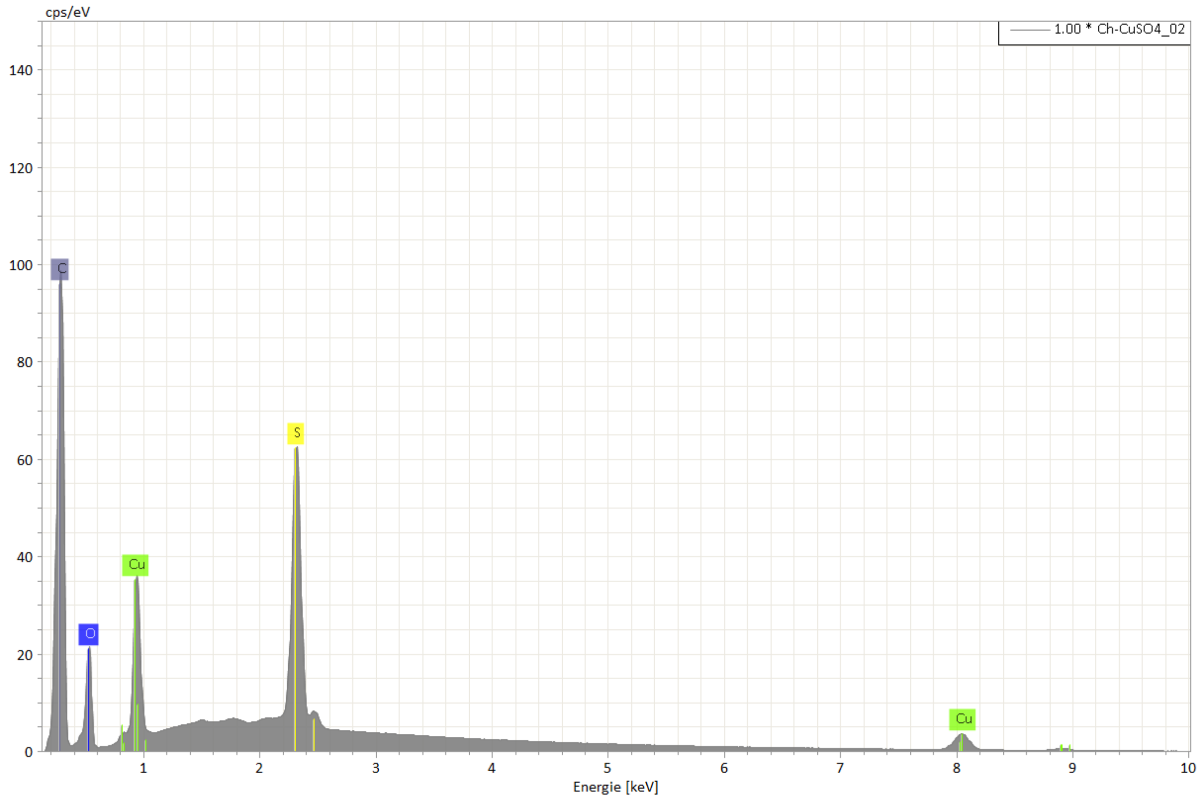

Figure S32: EDX pattern of Ch-$CuSO_4$ (c_0 = 4700 mg L^{-1}) after the adsorption process.

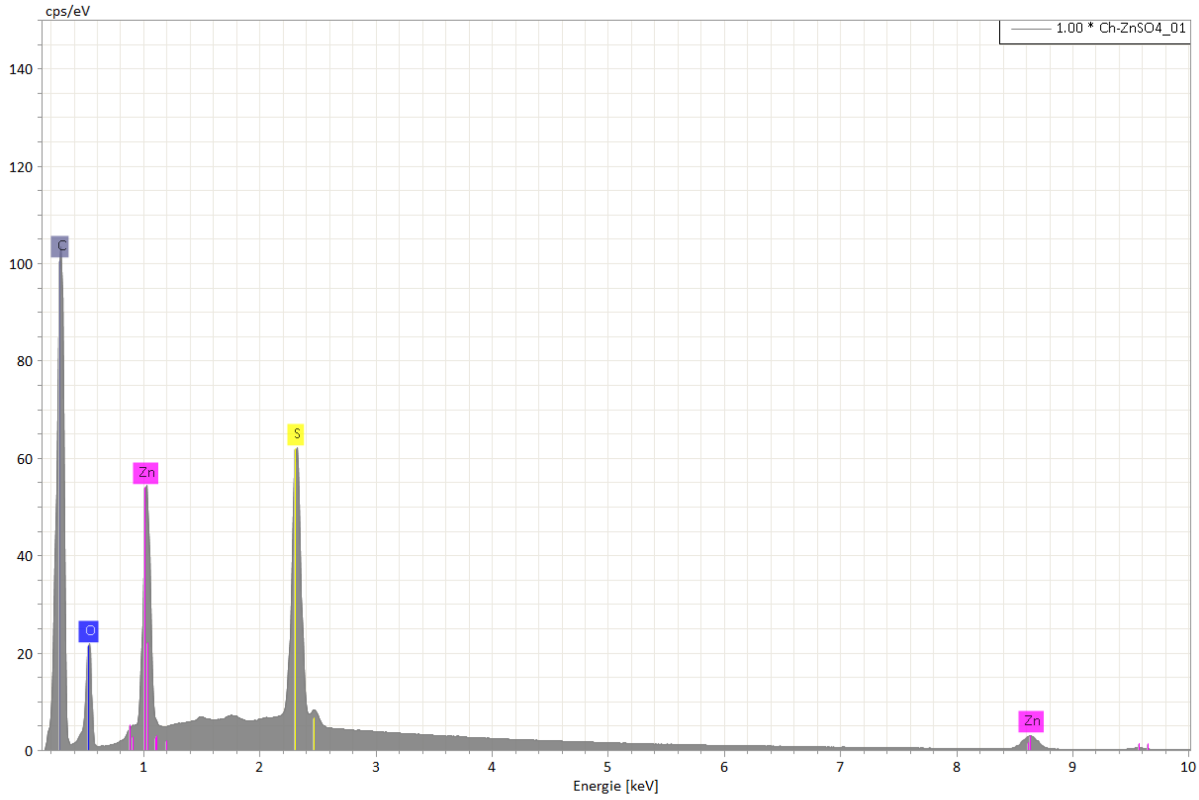

Figure S33: EDX pattern of Ch-$ZnSO_4$ (c_0 = 5200 mg L^{-1}) after the adsorption process.

2.5 SEM-EDX

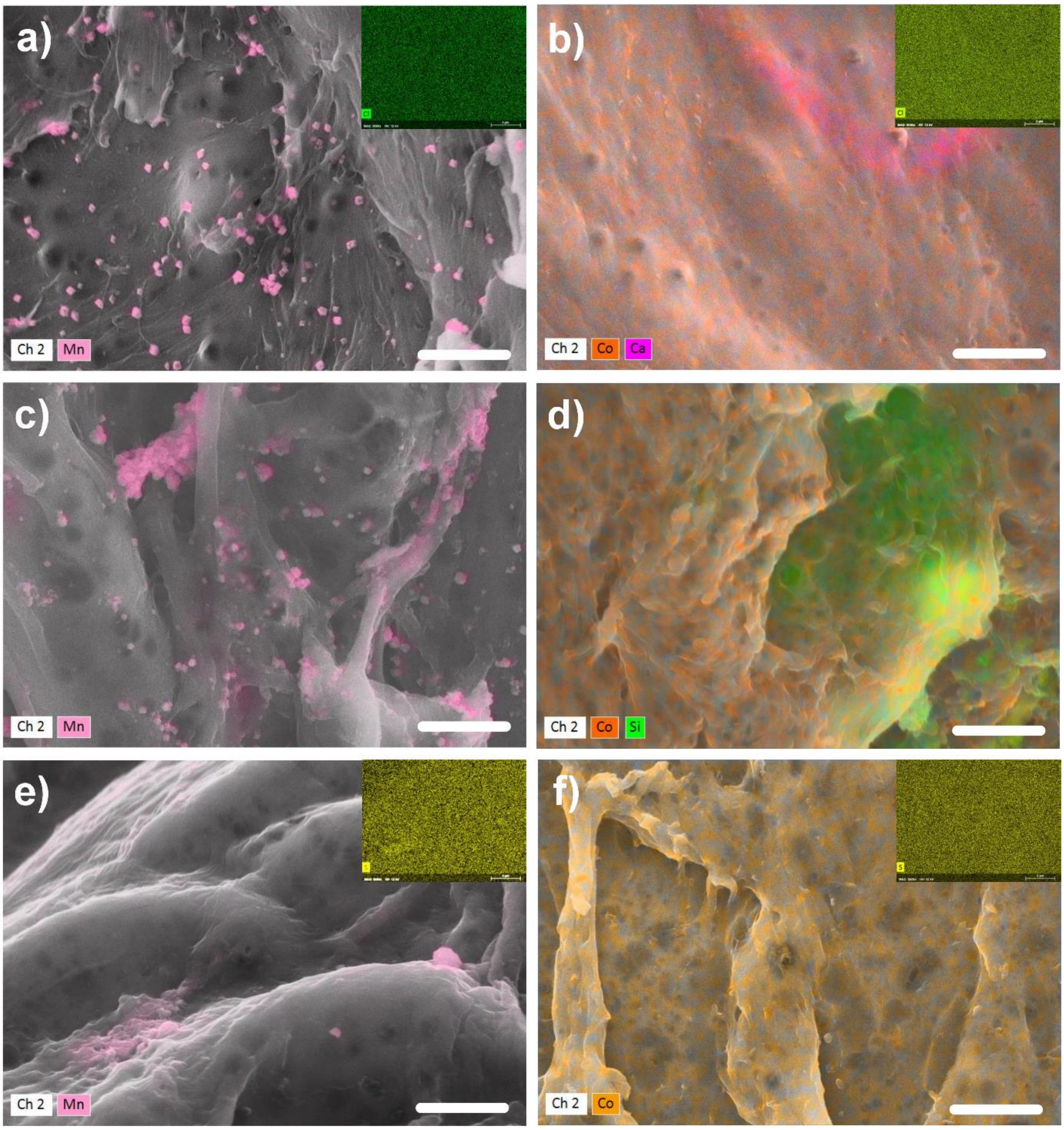

Figure S34 SEM-EDX images of a) Ch-$MnCl_2$ c_0 = 4100 mg L^{-1} (distribution of Cl, top corner right-hand-side), b) Ch-$CoCl_2$ c_0 = 4000 mg L^{-1} (distribution of Cl, top corner right-hand-side), c) Ch-$Mn(NO_3)_2$ c_0 = 5800 mg L^{-1}, d) Ch-$Co(NO_3)_2$ c_0 = 6200 mg L^{-1}, e) Ch-$MnSO_4$ c_0 = 5900 mg L^{-1} (distribution of S, top corner right-hand-side), and f) Ch-$CoSO_4$ c_0 = 4800 mg L^{-1} (distribution of S, top corner right-hand-side) after the adsorption process, scale bar: 3 μm.

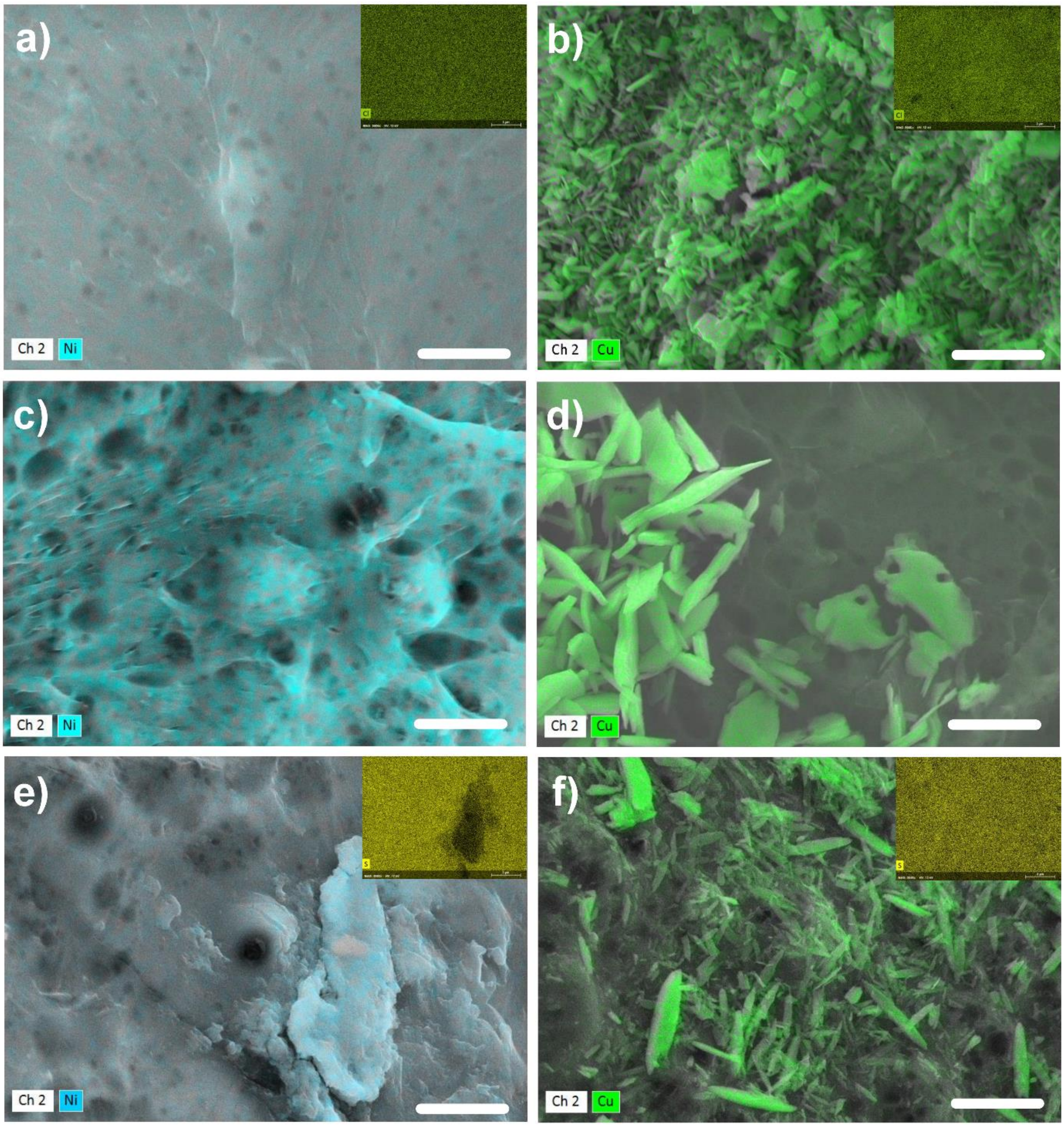

Figure S35: SEM-EDX images of a) Ch-$NiCl_2$ c_0 = 4200 mg L^{-1} (distribution of Cl, top corner right-hand-side), b) Ch-$CuCl_2$ c_0 = 4000 mg L^{-1} (distribution of Cl, top corner right-hand-side), c) Ch-$Ni(NO_3)_2$ c_0 = 6200 mg L^{-1}, d) Ch-$Cu(NO_3)_2$ c_0 = 5800 mg L^{-1}, e) Ch-$NiSO_4$ c_0 = 5600 mg L^{-1} (distribution of S, top corner right-hand-side), and f) Ch-$CuSO_4$ c_0 = 4700 mg L^{-1} (distribution of S, top corner right-hand-side) after the adsorption process, scale bar: 3 μm.

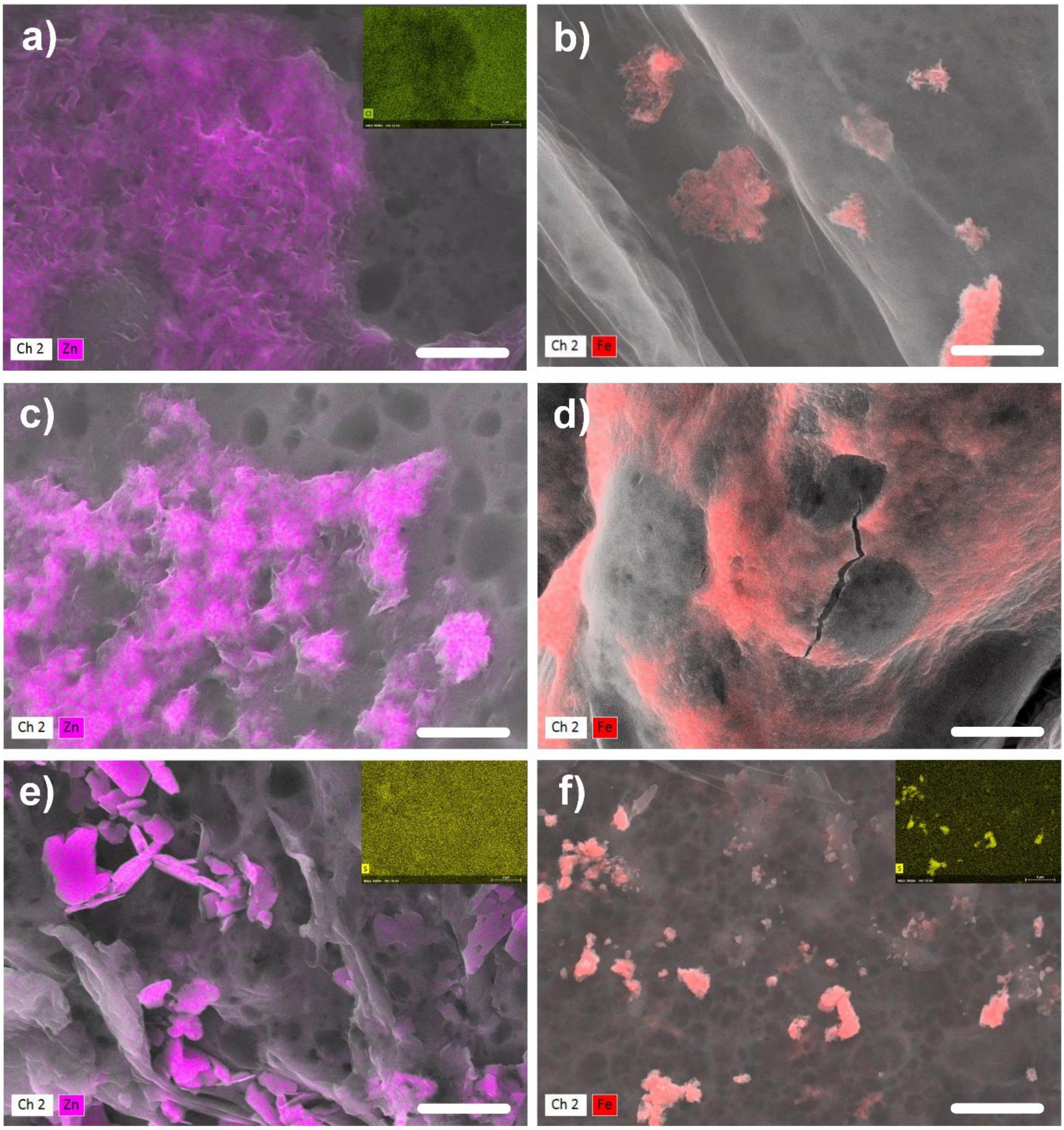

Figure S36: SEM-EDX images of a) Ch-$ZnCl_2$ c_0 = 4100 mg L^{-1} (distribution of Cl, top corner right-hand-side), b) Ch-$FeCl_2$ c_0 = 650 mg L^{-1}c) Ch-$Zn(NO_3)_2$ c_0 = 3800 mg L^{-1}, d) Ch-$Fe(NO_3)_2$ c_0 = 410 mg L^{-1}, e) Ch-$ZnSO_4$ c_0 = 5200 mg L^{-1} (distribution of S, top corner right-hand-side), and f) Ch-$FeSO_4$ c_0 = 5900 mg L^{-1} (distribution of S, top corner right-hand-side) after the adsorption process, scale bar: 3 µm.

2.6 Zetapotential pH Measurement

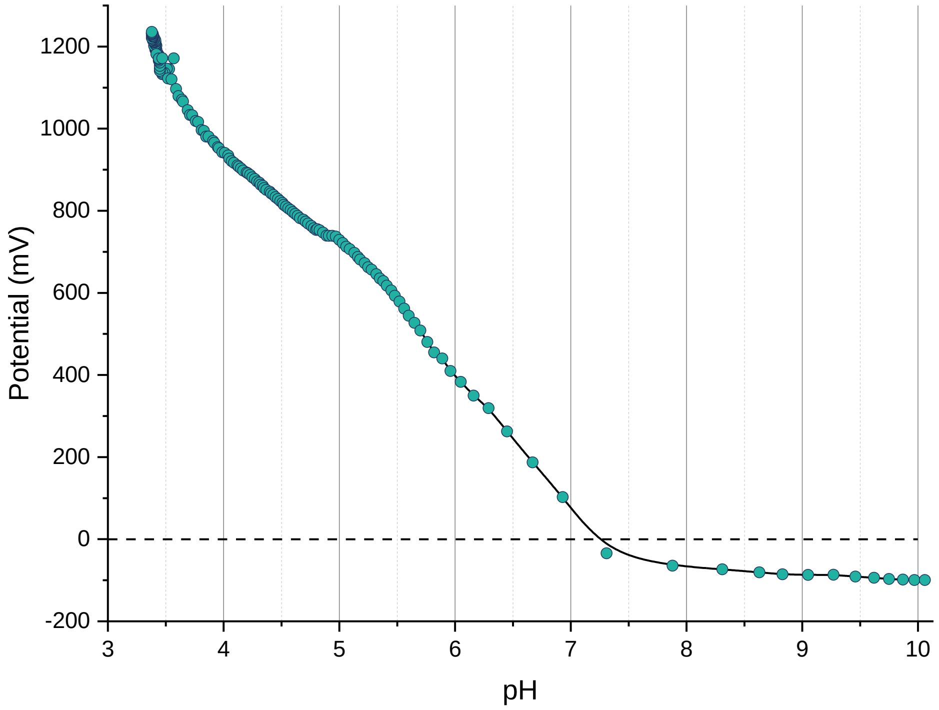

Figure S37: Zetapotential measurement as a function of pH for Ch85/400/A2.

2.7 Column Experiment

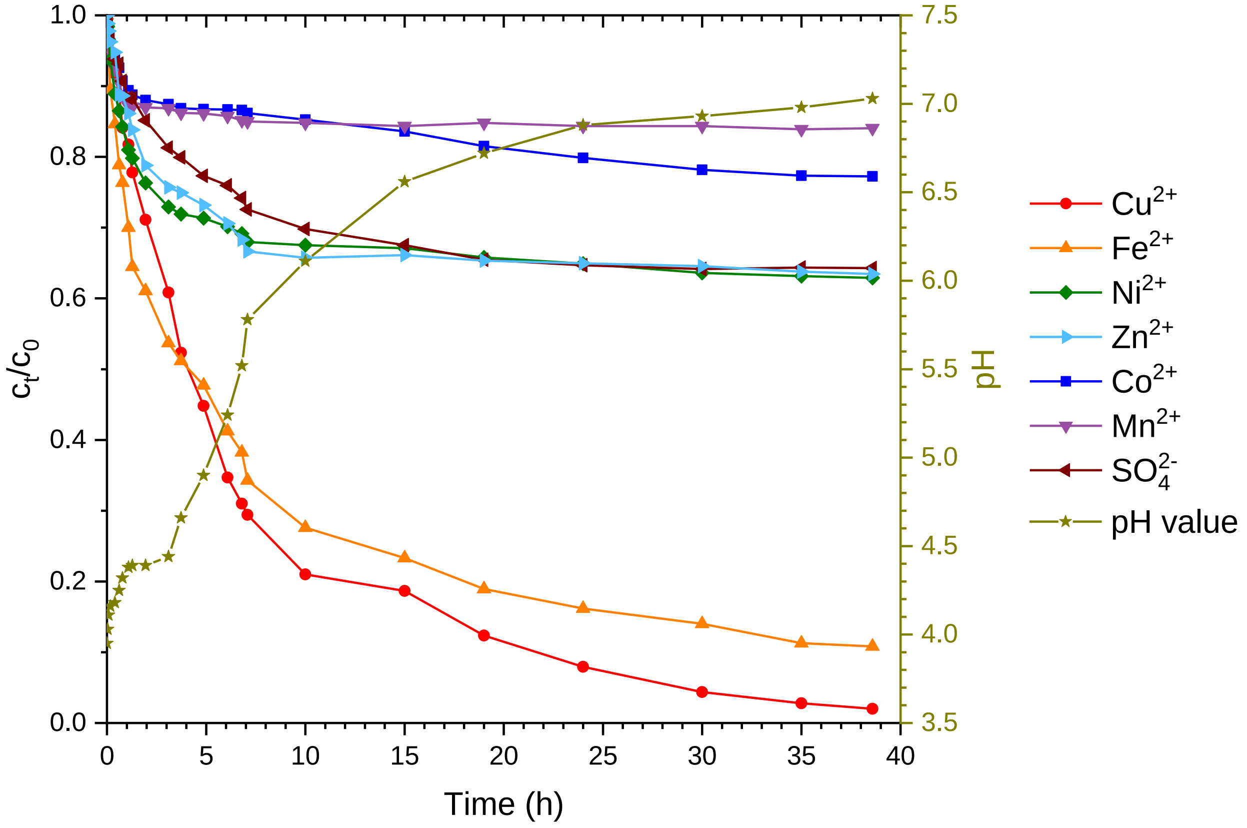

Figure S38: Column experiment as c_t/c_0 vs. time plot with all six heavy metal sulfate salts. The column was filled with the chitosan flakes and the concentration of the heavy metal ions and sulfate was measured as a function of time, including the change in pH. The initial concentrations of each heavy metal ion in the solution was $c_0(Mn^{2+})$ = 4.06 mmol L^{-1}; $c_0(Fe^{2+})$ = 4.05 mmol L^{-1}; $c_0(Co^{2+})$ = 4.08 mmol L^{-1}; $c_0(Ni^{2+})$ = 3.91 mmol L^{-1}; $c_0(Cu^{2+})$ = 3.97 mmol L^{-1}; $c_0(Zn^{2+})$ = 3.93 mmol L^{-1}; $c_0(SO_4^{2-})$ = 28.64 mmol L^{-1}.

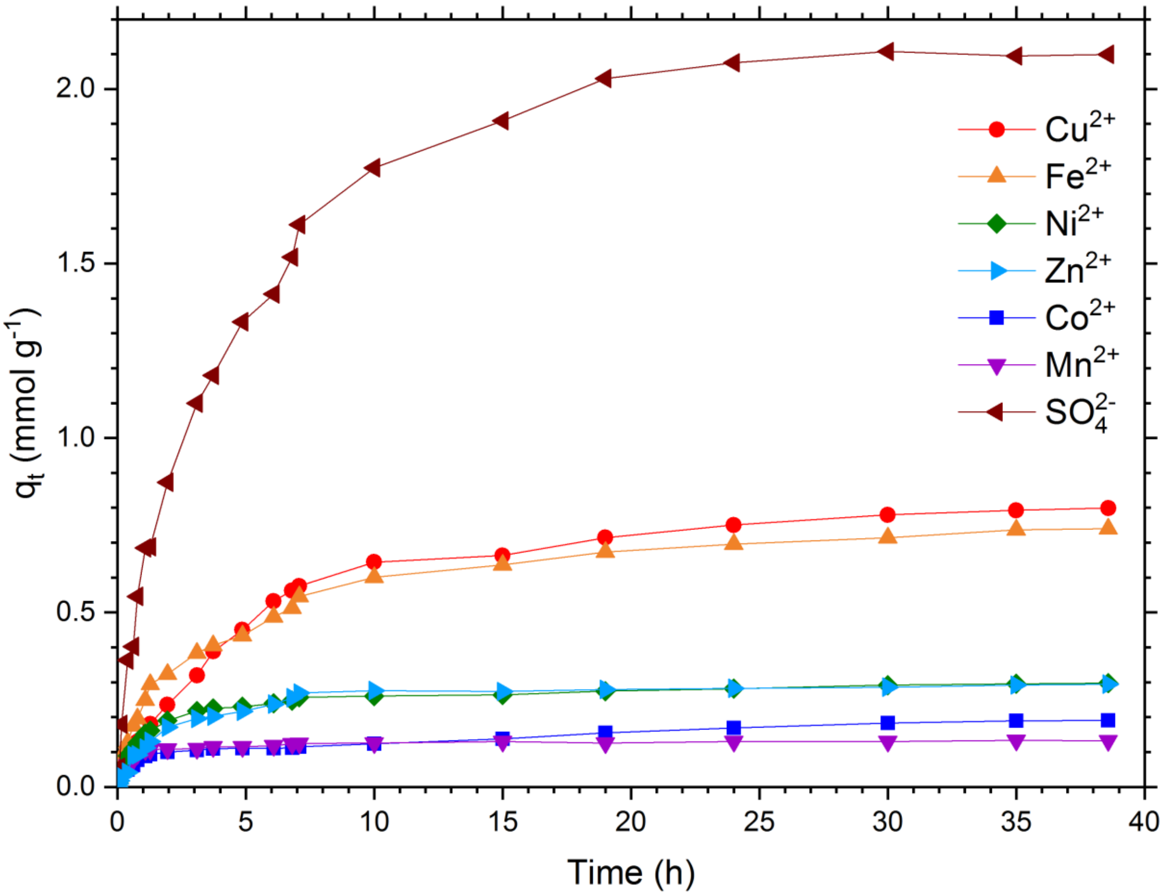

Figure 39. Column experiment as q(t) vs. time plot with all six heavy metal sulfate salts. The column was filled with the chitosan flakes and the concentration of the heavy metal ions and sulfate was measured as a function of time, including the change in pH. The initial concentrations of each heavy metal ion in the solution was $c_0(Mn^{2+})$ = 4.06 mmol L^{-1}; $c_0(Fe^{2+})$ = 4.05 mmol L^{-1}; $c_0(Co^{2+})$ = 4.08 mmol L^{-1}; $c_0(Ni^{2+})$ = 3.91 mmol L^{-1}; $c_0(Cu^{2+})$ = 3.97 mmol L^{-1}; $c_0(Zn^{2+})$ = 3.93 mmol L^{-1}; $c_0(SO_4^{2-})$ = 28.64 mmol L^{-1}.

3 Classification of the obtained adsorption capacities with other materials

Table S2: Adsorption capacities for removal of manganese compounds from aqueous solutions with different adsorber materials.

Material	Adsorption capacity q (mg g^{-1})	Salt	Experimental conditions			Ref.
			pH$_0$	c$_0$ (mg L^{-1})	t, T	
**chitosan**	8.98	$MnSO_4$	4.7	2000	24 h, RT	this work
**volcanic ash geopolymer**	192	$MnSO_4$	6.3	1100	30 min, RT	[1]
**metakaolin based geopolymer**	72.34	$MnSO_4$	6	800	25 min, 30 °C	[2]
**activated carbon from coconut shell**	51.23	$Mn(NO_3)_2$	5.8	100	2 h, 25 °C	[3]
**cow bone charcoal**	29.59	$Mn(NO_3)_2$	5.1	100	110 min, 25 °C	[4]
**sodium alginate/graphene oxide double network hydrogel**	56.49	$MnCl_2$	6	200	9 h, 25 °C	[5]

Table S3: Adsorption capacities for removal of iron compounds from aqueous solutions with different adsorber materials.

Material	Adsorption capacity q (mg g^{-1})	Salt	Experimental conditions			Ref.
			pH$_0$	c$_0$ (mg L^{-1})	t, T	
**chitosan**	128.8	$FeSO_4$	4	2000	24 h, RT	this work
**activated carbon from coconut shell**	58.76	$FeSO_4$	5.8	100	2 h, 25 °C	[3]
**cow bone charcoal**	31.43	$FeSO_4$	5.1	100	110 min, 25 °C	[4]

Table S4: Adsorption capacities for removal of cobalt compounds from aqueous solutions with different adsorber materials.

Material	Adsorption capacity q (mg g^{-1})	Salt	Experimental conditions			Ref.
			pH$_0$	c$_0$ (mg L^{-1})	t, T	
**chitosan**	79.8	$CoSO_4$	4.6	2000	24 h, RT	this work
**activated carbon prepared from hazelnut shells**	13.88	$CoCl_2$	6	45.55	30 °C	[6]
**amination graphene oxide**	116.35	$CoNO_3$	6	1000	12 h, 25 °C	[7]
**metakaolin based geopolymer**	69.23	$Co(NO_3)_2$	6	800	15 min 30 °C	[2]
**nanosponge biopolymer composite**	7.812	$Co(NO_3)_2$	7.5	10	1,5 h, 25 °C	[8]

Table S5: Adsorption capacities for removal of nickel compounds from aqueous solutions with different adsorber materials.

Material	Adsorption capacity q (mg g^{-1})	Salt	Experimental conditions			Ref.
			pH_0	c_0 (mg L^{-1})	t, T	
**chitosan**	105.6	$NiSO_4$	5.4	2000	24 h, RT	this work
**poly(vinylpyrrolidone) hydrogel**	13.43	$NiCl_2$	8	5	6 h, 25 °C	[9]
**poly(vinylpyrrolidone-comethylacrylate) hydrogel**	71.52	$NiCl_2$	8	5	6 h, 25 °C	[9]
**activated carbon from coconut shell**	67.56	$Ni(NO_3)_2$	5.8	100	2 h, 25 °C	[3]
**Cow bone charcoal**	32.54	$Ni(NO_3)_2$	5.1	100	110 min, 25 °C	[4]
**chitosan**	66.3	$Ni(NO_3)_2$	-	318	5d 30 °C	[10]
**chitosan**	49.89	$NiSO_4$	-	318	5d, 30 °C	[10]

Table S6: Adsorption capacities for removal of copper compounds from aqueous solutions with different adsorber materials.

Material	Adsorption capacity q (mg g^{-1})	Salt	Experimental conditions			Ref.
			pH_0	c_0 (mg L^{-1})	t, T	
**chitosan**	149.4	$CuSO_4$	4.5	2000	24 h, RT	this work
**chitosan**	84.2	$CuCl_2$	4.5	2000	24 h, RT	this work
**chitosan**	99.6	$Cu(NO_3)_2$	4.5	2000	24 h, RT	this work
**chitosan-gum arabic nanoparticles**	303.03-344.83	$CuSO_4$	6	635.5	24 h	[11]
**chitosan**	103	$CuCl_2$	4.7-5.7	500	1.5 h, RT	[12]
**chitosan-glutaraldehyde**	58.1	$CuCl_2$	4.7-5.7	500	3 h, RT	[12]
**chitosan-epichlorohydrin**	52.6	$CuCl_2$	4.7-5.7	500	3 h, RT	[12]
**chitosan-ethylene glycol diglycidyl ether**	89.3	$CuCl_2$	4.7-5.7	500	3 h, RT	[12]
**poly(vinylpyrrolidone) hydrogel**	86.66	$CuCl_2$	8	5	6 h, 25 °C	[9]
**poly(vinylpyrrolidone-comethylacrylate) hydrogel**	98.53	$CuCl_2$	8	5	6 h, 25 °C	[9]
**activated carbon from coconut shell**	76.66	$Cu(NO_3)_2$	5.8	100	2 h, 25 °C	[3]
**cow bone charcoal**	35.44	$Cu(NO_3)_2$	5.1	100	110 min, 25 °C	[4]
**chitosan**	198.9	$CuSO_4$	-	318	5 d, 30 °C	[10]
**chitosan**	136	$Cu(NO_3)_2$	-	318	5 d, 30 °C	[10]
**polyethylenimine cellulose/sodium alginate**	177.1	$Cu(NO_3)_2$	5.5	1000	8 h, RT	[13]

Table 7. Adsorption capacities for removal of zinc compounds from aqueous solutions with different adsorber materials.

Material	Adsorption capacity q (mg g^{-1})	Salt	Experimental conditions			Ref.
			pH$_0$	c$_0$ (mg L^{-1})	t, T	
**chitosan**	104.6	$ZnSO_4$	5.2	2000	24 h, RT	this work
**chitosan**	61.4	$ZnCl_2$	4.7-5.7	500	1.5 h, RT	[12]
**chitosan-glutaraldehyde**	37.7	$ZnCl_2$	4.7-5.7	500	3 h, RT	[12]
**chitosan-epichlorohydrin**	16.4	$ZnCl_2$	4.7-5.7	500	3 h, RT	[12]
**chitosan-ethylene glycol diglycidyl ether**	51.0	$ZnCl_2$	4.7-5.7	500	3 h, RT	[12]
**poly(vinylpyrrolidone) hydrogel**	32.20	$ZnCl_2$	8	5	6 h, 25 °C	[9]
**poly(vinylpyrrolidone-comethylacrylate) hydrogel**	61.941	$ZnCl_2$	8	5	6 h, 25 °C	[9]
**chitosan**	289	$ZnSO_4$	-	318	5 d, 30 °C	[10]
**chitosan**	79.1	$Zn(NO_3)_2$	-	318	5 d, 30 °C	[10]
**polyethylenimine cellulose/sodium alginate**	110.2	$Zn(NO_3)_2$	5.5	1000	8 h, RT	[13]

4 References

1. Anguile, J.J.; Ona-Mbega, M.; Makani, T.; Ketcha-Mbadcam, J. Adsorption of manganese (II) ions from aqueous solution on to volcanic ash and geopolymer based volcanic ash. *International Journal of Basic and Applied Chemical Sciences* **2013**, *3*, 7-18.
2. Kara, I.; Tunc, D.; Sayin, F.; Akar, S.T. Study on the performance of metakaolin based geopolymer for Mn(II) and Co(II) removal. *Applied Clay Science* **2018**, *161*, 184-193, doi:10.1016/j.clay.2018.04.027.
3. Moreno-Piraján, J.C.; Garcia-Cuello, V.S.; Giraldo, L. The removal and kinetic study of Mn, Fe, Ni and Cu ions from wastewater onto activated carbon from coconut shells. *Adsorption* **2010**, *17*, 505-514, doi:10.1007/s10450-010-9311-5.
4. Moreno, J.C.; Gómez, R.; Giraldo, L. Removal of Mn, Fe, Ni and Cu Ions from Wastewater Using Cow Bone Charcoal. *Materials* **2010**, *3*, 452-466, doi:10.3390/ma3010452.
5. Yang, X.; Zhou, T.; Ren, B.; Hursthouse, A.; Zhang, Y. Removal of Mn (II) by Sodium Alginate/Graphene Oxide Composite Double-Network Hydrogel Beads from Aqueous Solutions. *Sci Rep* **2018**, *8*, 10717, doi:10.1038/s41598-018-29133-y.
6. Demirbaş, E. Adsorption of Cobalt(II) Ions from Aqueous Solution onto Activated Carbon Prepared from Hazelnut Shells. *Adsorption Science & Technology* **2003**, *21*, 951-963, doi:10.1260/02636170360744380.
7. Fang, F.; Kong, L.; Huang, J.; Wu, S.; Zhang, K.; Wang, X.; Sun, B.; Jin, Z.; Wang, J.; Huang, X.-J. Removal of cobalt ions from aqueous solution by an amination graphene oxide nanocomposite. *Journal of hazardous materials* **2014**, *270*, 1-10.
8. Taka, A.L.; Fosso-Kankeu, E.; Pillay, K.; Mbianda, X.Y. Removal of cobalt and lead ions from wastewater samples using an insoluble nanosponge biopolymer composite: adsorption isotherm, kinetic, thermodynamic, and regeneration studies. *Environ Sci Pollut Res Int* **2018**, *25*, 21752-21767, doi:10.1007/s11356-018-2055-6.
9. Kemik, Ö.F.; Ngwabebhoh, F.A.; Yildiz, U. A response surface modelling study for sorption of Cu2+, Ni2+, Zn2+ and Cd2+ using chemically modified poly(vinylpyrrolidone) and poly(vinylpyrrolidone-co-methylacrylate) hydrogels. *Adsorption Science & Technology* **2016**, *35*, 263-283, doi:10.1177/0263617416674950.
10. Wu, F.-C.; Tseng, R.-L.; Juang, R.-S. A review and experimental verification of using chitosan and its derivatives as adsorbents for selected heavy metals. *Journal of Environmental Management* **2010**, *91*, 798-806.
11. Abreu, F.O.M.d.S.; Silva, N.A.d.; Sipauba, M.d.S.; Pires, T.F.M.; Bomfim, T.A.; Monteiro Junior, O.A.d.C.; Forte, M.M.d.C. Chitosan and gum arabic nanoparticles for heavy metal adsorption. *Polímeros* **2018**, *28*, 231-238, doi:10.1590/0104-1428.02317.
12. Kamari, A.; Pulford, I.; Hargreaves, J. Binding of heavy metal contaminants onto chitosans–an evaluation for remediation of metal contaminated soil and water. *Journal of environmental management* **2011**, *92*, 2675-2682.
13. Zhan, W.; Xu, C.; Qian, G.; Huang, G.; Tang, X.; Lin, B. Adsorption of Cu(ii), Zn(ii), and Pb(ii) from aqueous single and binary metal solutions by regenerated cellulose and sodium alginate chemically modified with polyethyleneimine. *RSC Advances* **2018**, *8*, 18723-18733, doi:10.1039/c8ra02055h.
